# Duration of cardiopulmonary resuscitation and outcomes for adults with in-hospital cardiac arrest: retrospective cohort study

**DOI:** 10.1136/bmj-2023-076019

**Published:** 2024-02-07

**Authors:** Masashi Okubo, Sho Komukai, Lars W Andersen, Robert A Berg, Michael C Kurz, Laurie J Morrison, Clifton W Callaway

**Affiliations:** 1 Department of Emergency Medicine, University of Pittsburgh School of Medicine, Pittsburgh, PA 15261, USA; 2 Division of Biomedical Statistics, Department of Integrated Medicine, Osaka University Graduate School of Medicine, Osaka, Japan; 3 Department of Clinical Medicine, Aarhus University, Aarhus, Denmark; 4 Department of Anesthesiology and Intensive Care, Aarhus University Hospital, Aarhus, Denmark; 5 Prehospital Emergency Medical Services, Central Denmark Region, Denmark; 6 Department of Anesthesiology and Critical Care Medicine, Children’s Hospital of Philadelphia, University of Pennsylvania Perelman School of Medicine, Philadelphia, PA, USA; 7 Section of Emergency Medicine, Department of Medicine, University of Chicago School of Medicine, Chicago, IL, USA; 8 Division of Emergency Medicine, Department of Medicine, University of Toronto, Sunnybrook Health Sciences Centre, Toronto, ON, Canada

## Abstract

**Objective:**

To quantify time dependent probabilities of outcomes in patients after in-hospital cardiac arrest as a function of duration of cardiopulmonary resuscitation, defined as the interval between start of chest compression and the first return of spontaneous circulation or termination of resuscitation.

**Design:**

Retrospective cohort study.

**Setting:**

Multicenter prospective in-hospital cardiac arrest registry in the United States.

**Participants:**

348 996 adult patients (≥18 years) with an index in-hospital cardiac arrest who received cardiopulmonary resuscitation from 2000 through 2021.

**Main outcome measures:**

Survival to hospital discharge and favorable functional outcome at hospital discharge, defined as a cerebral performance category score of 1 (good cerebral performance) or 2 (moderate cerebral disability). Time dependent probabilities of subsequently surviving to hospital discharge or having favorable functional outcome if patients pending the first return of spontaneous circulation at each minute received further cardiopulmonary resuscitation beyond the time point were estimated, assuming that all decisions on termination of resuscitation were accurate (that is, all patients with termination of resuscitation would have invariably failed to survive if cardiopulmonary resuscitation had continued for a longer period of time).

**Results:**

Among 348 996 included patients, 233 551 (66.9%) achieved return of spontaneous circulation with a median interval of 7 (interquartile range 3-13) minutes between start of chest compressions and first return of spontaneous circulation, whereas 115 445 (33.1%) patients did not achieve return of spontaneous circulation with a median interval of 20 (14-30) minutes between start of chest compressions and termination of resuscitation. 78 799 (22.6%) patients survived to hospital discharge. The time dependent probabilities of survival and favorable functional outcome among patients pending return of spontaneous circulation at one minute’s duration of cardiopulmonary resuscitation were 22.0% (75 645/343 866) and 15.1% (49 769/328 771), respectively. The probabilities decreased over time and were <1% for survival at 39 minutes and <1% for favorable functional outcome at 32 minutes’ duration of cardiopulmonary resuscitation.

**Conclusions:**

This analysis of a large multicenter registry of in-hospital cardiac arrest quantified the time dependent probabilities of patients’ outcomes in each minute of duration of cardiopulmonary resuscitation. The findings provide resuscitation teams, patients, and their surrogates with insights into the likelihood of favorable outcomes if patients pending the first return of spontaneous circulation continue to receive further cardiopulmonary resuscitation.

**Figure:**
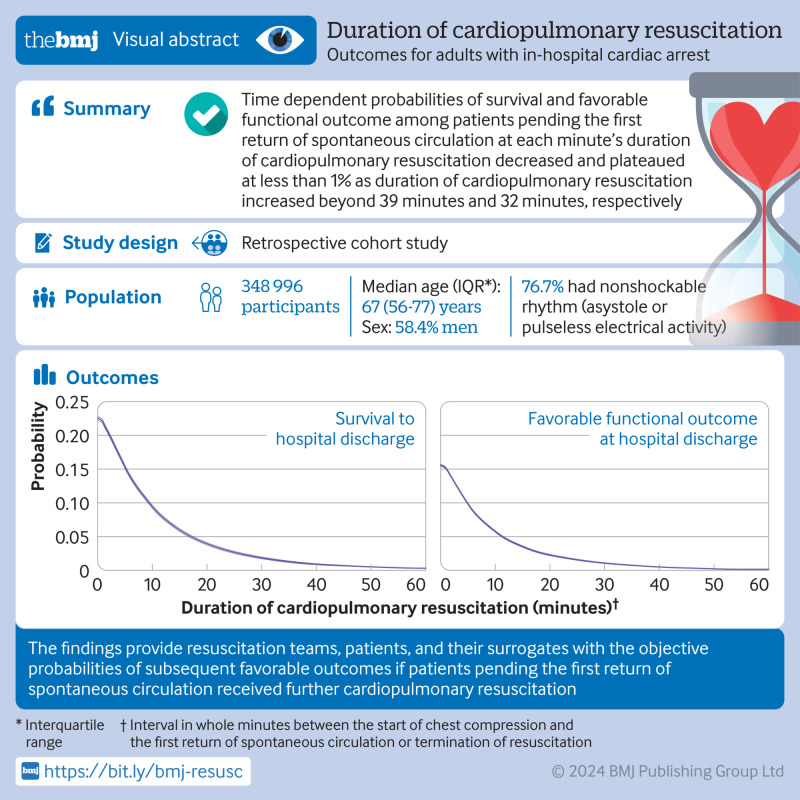


## Introduction

In-hospital cardiac arrest is an important public health problem, affecting approximately 300 000 adults annually in the United States, with a high mortality rate.^1^ ^2^ The survival rate after in-hospital cardiac arrest in the US improved from 2000 to 2010 and has remained plateaued after 2010, with approximately 25% of patients surviving to hospital discharge.^3^ ^4^

Achieving return of spontaneous circulation is the first step toward long term survival and favorable functional recovery. However, for nearly half of patients with in-hospital cardiac arrest, resuscitative efforts are terminated without achievement of return of spontaneous circulation.^5^ When patients do not achieve return of spontaneous circulation despite cardiopulmonary resuscitation, clinical providers face challenges in deciding how long to continue cardiopulmonary resuscitation. For patients with out-of-hospital cardiac arrest, previous studies showed that longer duration of pre-hospital cardiopulmonary resuscitation before return of spontaneous circulation was associated with poor outcomes for patients.^6^ ^7^ ^8^ ^9^ ^10^ However, the association of duration of cardiopulmonary resuscitation with patients’ outcomes has not been fully investigated for in-hospital cardiac arrest. The 2020 International Consensus on Cardiopulmonary Resuscitation and Emergency Cardiovascular Care Science With Treatment Recommendations of the Education, Implementation, and Teams Task Force was unable to make recommendations on when to terminate cardiopulmonary resuscitation for in-hospital cardiac arrest.^11^ This highlights existing gaps in knowledge and the importance of further evaluation of the effect of duration of cardiopulmonary resuscitation on patients’ outcomes after in-hospital cardiac arrest.

Our primary objective was to quantify the time dependent probabilities of favorable outcomes as a function of duration of cardiopulmonary resuscitation among patients pending the first return of spontaneous circulation at each minute’s duration of cardiopulmonary resuscitation who later attained return of spontaneous circulation or had termination of resuscitation. Our secondary objective was to quantify the time dependent probabilities of favorable outcomes as a function of duration of cardiopulmonary resuscitation among patients who had the first return of spontaneous circulation before or at each time point. We also did stratified analyses to investigate whether clinical features and patients’ phenotypes modified the association between duration of cardiopulmonary resuscitation and favorable outcomes.

## Methods

### Study design and setting

This is an analysis of the Get With The Guidelines—Resuscitation (GWTG-R) registry, a multicenter prospective quality improvement registry of in-hospital cardiac arrest in the US. The GWTG programs are provided by the American Heart Association. Details of the registry were previously reported elsewhere.^12^ Data are collected on all patients with in-hospital cardiac arrest in the participating hospitals who did not have existing do not resuscitate orders and who received cardiopulmonary resuscitation.^4^ ^13^ Cardiac arrest was defined as pulselessness requiring chest compression, defibrillation, or both.^4^ ^13^ Several case finding methods were used to consecutively collect cases of in-hospital cardiac arrest, including centralized collection of cardiac arrest flow sheets, review of hospital paging systems, and regular checks of cardiopulmonary resuscitation code carts, pharmacy tracer medication records, and hospital billing charges for use of resuscitation medications.^4^ Research or quality assurance staff collect information on in-hospital cardiac arrest events from hospital medical records and cardiac arrest documentation forms.^14^ The registry used the standardized Utstein template for the definitions of clinical variables and outcomes.^15^ ^16^ Data integrity is ensured by rigorous training and certification of hospital staff, use of standardized software with internal data checks, and a periodic re-abstraction process.^4^ ^12^ ^13^

### Study participants

We included adult patients (≥18 years) with an index in-hospital cardiac arrest who received cardiopulmonary resuscitation between 2000 and 2021. We excluded patients with extracorporeal membrane oxygenation in place before the start of cardiopulmonary resuscitation, missing data to classify duration of cardiopulmonary resuscitation, do not resuscitate orders before chest compression, missing data on survival to hospital discharge, duration of cardiopulmonary resuscitation greater than 120 minutes,^17^ termination of resuscitation because of do not resuscitate orders, or extracorporeal membrane oxygenation after the start of cardiopulmonary resuscitation. In an analysis of favorable functional outcome, we further excluded patients with missing functional outcome at hospital discharge.

### Exposure

The main exposure was duration of cardiopulmonary resuscitation in minutes, defined as an interval in whole minutes between the start of chest compression and the first return of spontaneous circulation or termination of resuscitation. We defined return of spontaneous circulation as return of adequate pulse by palpation, Doppler, or arterial blood pressure waveform. Data on the start of cardiopulmonary resuscitation, the first return of spontaneous circulation, and termination of resuscitation were initially recoded at the time of the in-hospital cardiac arrest events by the clinical team and subsequently entered onto the GWTG-R database by research or quality assurance staff.^14^

### Outcome measures

Our outcome measures were survival to hospital discharge and favorable functional outcome at hospital discharge, defined as cerebral performance category (CPC) score 1 or 2.^16^ The CPC is a 5 point functional scale; a CPC score of 1 represents good cerebral performance, 2 represents moderate cerebral disability, 3 represents severe cerebral disability, 4 represents coma or vegetative state, and 5 represents brain death.^16^ ^18^ ^19^

### Statistical analysis

We stratified patients by presence or absence of return of spontaneous circulation and reported characteristics of patients and cardiac arrests. We also reported differences in these characteristics with standardized mean differences between patients with and without missing duration of cardiopulmonary resuscitation, survival to hospital discharge, or functional outcome at hospital discharge. We considered an absolute standardized mean difference within 0.25 to be a small difference.^20^

#### Cumulative proportion of patients achieving first return of spontaneous circulation over time stratified by patients with outcomes

Using the Kaplan-Meier estimate, we constructed simple curves of the cumulative proportion of patients achieving the first return of spontaneous circulation over time, stratified by survival (among patients who survived to hospital discharge or among patients who had return of spontaneous circulation and subsequently died before hospital discharge) and functional outcome (among patients with favorable functional outcome (CPC score 1 or 2) at hospital discharge, among patients with unfavorable functional outcome (CPC score 3 or 4) at hospital discharge, or among patients who had return of spontaneous circulation and subsequently died before hospital discharge). Using the Greenwood formula for the estimated standard error of the Kaplan-Meier estimate, we also estimated the 95th and 99th centiles of duration of cardiopulmonary resuscitation for each stratified curve with 95% confidence intervals.

#### Time dependent probabilities of outcomes among patients pending first return of spontaneous circulation at each minute’s duration of cardiopulmonary resuscitation

We calculated time dependent probabilities for the outcomes as a function of duration of cardiopulmonary resuscitation. Firstly, we calculated time dependent probabilities of survival and favorable functional outcome among patients pending the first return of spontaneous circulation at each minute’s duration of cardiopulmonary resuscitation. The numerator was the number of patients who were pending the first return of spontaneous circulation at each minute and subsequently had each outcome. The denominator was the number of patients pending the first return of spontaneous circulation at each minute. This time dependent probability represented the probability of subsequently surviving to hospital discharge or having favorable functional outcome if the patients pending the first return of spontaneous circulation at that time point received further cardiopulmonary resuscitation beyond the time point (supplementary methods and figure A).

We calculated two time dependent probabilities of each outcome among patients pending the first return of spontaneous circulation at each minute, as we defined two denominators including and excluding patients with termination of resuscitation before or at each time point (supplementary methods and figure A). As a primary analysis, we included patients who had termination of resuscitation before or at each minute’s duration of cardiopulmonary resuscitation in the denominator (supplementary methods and figure A). Use of this denominator provides probabilities of having outcomes among the overall study population if patients pending the first return of spontaneous circulation had further cardiopulmonary resuscitation beyond that time point, assuming that all decisions on termination of resuscitation were accurate and that the patients who had termination of resuscitation never had outcomes, even if the patients would have had longer duration of cardiopulmonary resuscitation beyond the time point of termination of resuscitation. We reported duration of cardiopulmonary resuscitation when this probability became less than 1%, using traditional medical futility, a likelihood of survival of less than 1%.^21^ ^22^

As a sensitivity analysis, another denominator included only patients who were undergoing cardiopulmonary resuscitation at each minute pending the first return of spontaneous circulation, and excluding patients who had termination of resuscitation before or at each minute (supplementary methods and figure A). This denominator treated termination of resuscitation as a censoring event that is not informative on subsequent time dependent probabilities. Therefore, as duration of cardiopulmonary resuscitation increased, this denominator included only patients who were undergoing cardiopulmonary resuscitation and represented a selected population for whom the resuscitation team chose to provide prolonged cardiopulmonary resuscitation.

#### Time dependent probabilities of outcomes among patients who had first return of spontaneous circulation before or at each minute’s duration of cardiopulmonary resuscitation

Secondly, we calculated the time dependent probability for each outcome among patients who had the first return of spontaneous circulation before or at each time point (supplementary methods and figure A). This time dependent probability quantified the probability of surviving to hospital discharge or having favorable functional outcome once patients achieved the first return of spontaneous circulation before or at each time point. We carried out pointwise estimation of 95% confidence interval of each time dependent probability on the basis of the variance of binomial distribution.

#### Stratified analyses of time dependent probabilities

Additionally, we defined clinical features as age group, witness status, and initial rhythm. To evaluate whether the time dependent probabilities differed across clinical features of cardiac arrest, we stratified the time dependent probability curves on the basis of age group (<60 years, 60-79 years, or ≥80 years), witness status (witnessed or unwitnessed), and initial rhythm (shockable or non-shockable rhythm).^23^

We defined patients’ phenotype as each combination of age group, witness status, and initial rhythm. To investigate whether phenotypes of patients affect the relation between duration of cardiopulmonary resuscitation and outcomes, we plotted the time dependent probabilities for each phenotype.

#### Subgroup analysis

As the dataset included in-hospital cardiac arrests from 2000 through 2021, we did subgroup analyses including only patients from 2011 through 2021 to evaluate the recent in-hospital cardiac arrest data. We used Stata 16.1 and R software, version 4.0.2, for all statistical analyses

### Patient and public involvement

No patients or members of the public were involved in setting the research question or the outcome measures, nor were they involved in developing plans for the design or implementation of the study or asked to advise on interpretation or writing up of results.

## Results

**Fig 1:**
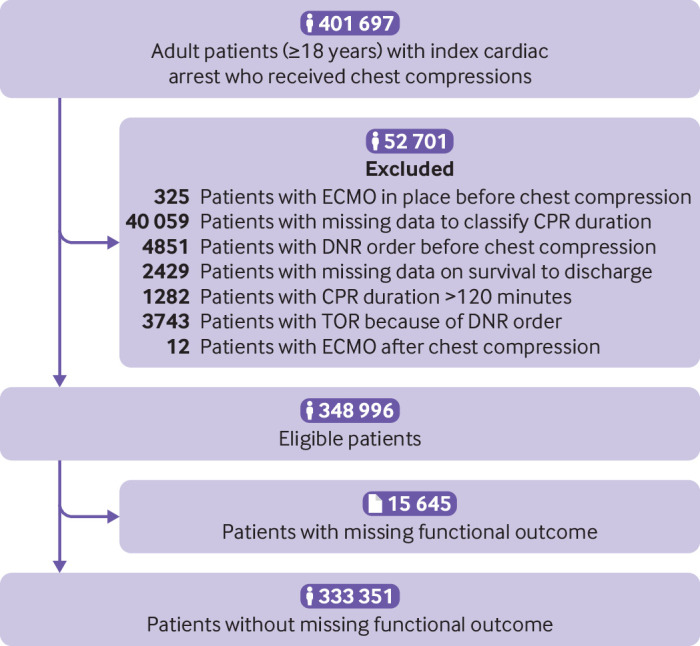
Patient flow. CPR=cardiopulmonary resuscitation; DNR=do not resuscitate; ECMO=extracorporeal membrane oxygenation; TOR=termination of resuscitation

We identified 401 697 patients with an index in-hospital cardiac arrest who received cardiopulmonary resuscitation (fig 1). After excluding those who met the exclusion criteria, we included 348 996 patients in our study. We further excluded 15 645 patients who were missing functional outcome at hospital discharge from the analysis of functional outcome.

Table 1 and table 2 show characteristics of patients and cardiac arrests. Among 348 996 included patients, 233 551 (66.9%) achieved return of spontaneous circulation and 78 799 (22.6%) survived to hospital discharge. The median interval between the start of chest compressions and the first return of spontaneous circulation was 7 (interquartile range 3-13) minutes among patients who had return of spontaneous circulation. The median interval between start of chest compressions and termination of resuscitation was 20 (14-30) minutes among patients who did not have return of spontaneous circulation. Among 333 351 patients without missing functional outcome at hospital discharge, 52 104 (15.6%) had favorable functional outcome. See supplementary table A for characteristics of patients and cardiac arrests in patients with and without missing duration of cardiopulmonary resuscitation, survival to hospital discharge, or functional outcome at hospital discharge. For all of characteristics of patients and most characteristics of cardiac arrests, the standardized differences were within 0.25, and the characteristics were similar.

**Table 1:** Patients’ characteristics. Values are numbers (percentages) unless stated otherwise

Characteristics	All patients (n=348 996)	Patients without ROSC (n=115 445)	Patients with ROSC (n=233 551)
Median (IQR) age, years	67 (56-77)	68 (56-79)	67 (56-76)
Sex:			
Male	203 883 (58.4)	69 495 (60.2)	134 388 (57.5)
Female	145 083 (41.6)	45 935 (39.8)	99 148 (42.5)
Unknown	24 (0.0)	10 (0.0)	14 (0.0)
Race:			
White	239 185 (68.5)	78 403 (67.9)	160 782 (68.8)
Black	76 329 (21.9)	25 518 (22.1)	50 811 (21.8)
Other*	11 316 (3.2)	3711 (3.2)	7605 (3.3)
Unknown	22 166 (6.4)	7813 (6.8)	14 353 (6.1)
Illness category:			
Medical			
Cardiac	123 297 (35.3)	40 251 (34.9)	83 046 (35.6)
Non-cardiac	153 957 (44.1)	52 934 (45.9)	101 023 (43.3)
Surgical			
Cardiac	21 351 (6.1)	5637 (4.9)	15 714 (6.7)
Non-cardiac	36 747 (10.5)	11 785 (10.2)	24 962 (10.7)
Trauma	12 288 (3.5)	4440 (3.8)	7848 (3.4)
Other†	1078 (0.3)	309 (0.3)	769 (0.3)
Unknown	278 (0.08)	89 (0.08)	189 (0.08)
Pre-existing condition:			
Cardiac			
History of myocardial infarction	48 428 (13.9)	15 635 (13.5)	32 793 (14.0)
Myocardial infarction, this admission	49 148 (14.1)	15 383 (13.3)	33 765 (14.5)
History of heart failure	71 367 (20.4)	22 369 (19.4)	48 998 (21.0)
Heart failure, this admission	51 101 (14.6)	15 914 (13.8)	35 187 (15.1)
Non-cardiac			
Respiratory insufficiency	147 083 (42.1)	46 149 (40.0)	100 934 (43.2)
Diabetes mellitus	107 010 (30.7)	31 775 (27.5)	75 235 (32.2)
Renal insufficiency	114 027 (32.7)	35 361 (30.6)	78 666 (33.7)
Metastatic or hematologic malignancy	37 304 (10.7)	13 625 (11.8)	23 679 (10.1)
Hypotension or hypoperfusion	89 250 (25.6)	29 544 (25.6)	59 706 (25.6)
Pneumonia	46 234 (13.2)	14 501 (12.6)	31 733 (13.6)
Baseline depression in CNS function	32 032 (9.2)	11 172 (9.7)	20 860 (8.9)
Metabolic or electrolyte abnormality	68 270 (19.6)	20 557 (17.8)	47 713 (20.4)
Sepsis	45 677 (13.1)	15 016 (13.0)	30 661 (13.1)
Acute CNS non-stroke event	28 326 (8.1)	8417 (7.3)	19 909 (8.5)
Hepatic insufficiency	26 617 (7.6)	8417 (7.3)	18 200 (7.8)
Acute stroke	12 675 (3.6)	4082 (3.5)	8593 (3.7)
Major trauma	15 107 (4.3)	5255 (4.6)	9852 (4.2)

CNS=central nervous system; IQR=interquartile range; ROSC=return of spontaneous circulation.

* Asian, Native Americans/Alaska Natives, “others,” and Native Hawaiians/Pacific Islanders.

† Obstetrics and “other.”

**Table 2:** Characteristics of cardiac arrests. Values are numbers (percentages) unless stated otherwise

Characteristics	All patients (n=348 996)	Patients without ROSC (n=115 445)	Patients with ROSC (n=233 551)
Year of cardiac arrest:			
2000-05	57 554 (16.5)	26 979 (23.4)	30 575 (13.1)
2006-10	81 615 (23.4)	30 228 (26.2)	51 387 (22.0)
2011-15	92 523 (26.5)	27 415 (23.7)	65 108 (27.9)
2016-21	117 304 (33.6)	30 823 (26.7)	86 481 (37.0)
Interventions in place at time of cardiac arrest:			
Non-invasive assisted ventilation	1610 (0.5)	413 (0.4)	1197 (0.5)
Mechanical ventilation	74 845 (21.4)	21 742 (18.8)	53 103 (22.7)
Dialysis	11 241 (3.2)	3716 (3.2)	7525 (3.2)
Implantable cardiac defibrillator	5978 (1.7)	1968 (1.7)	4010 (1.7)
Intra-arterial catheter	35 770 (10.2)	11 061 (9.6)	24 709 (10.6)
Electrocardiogram monitor	283 494 (81.2)	89 291 (77.3)	194 203 (83.2)
Pulse oximeter	245 878 (70.5)	76 983 (66.7)	168 895 (72.3)
Vasoactive agents	86 646 (24.8)	31 213 (27.0)	55 433 (23.7)
Antiarrhythmic agents	8571 (2.5)	2672 (2.3)	5899 (2.5)
Location of cardiac arrest:			
Emergency department	42 507 (12.2)	14 092 (12.2)	28 415 (12.2)
Floor with telemetry or step-down unit	54 914 (15.7)	17 472 (15.1)	37 442 (16.0)
Floor without telemetry	55 643 (15.9)	21 609 (18.7)	34 034 (14.6)
Intensive care unit or coronary care unit	163 799 (46.9)	53 014 (45.9)	110 785 (47.4)
Operating room, post-anesthesia care unit, cardiac catheterization laboratory, or diagnostic/interventional unit	24 781 (7.1)	6733 (5.8)	18 048 (7.7)
Other*	7198 (2.1)	2469 (2.1)	4729 (2.0)
Unknown	154 (0.04)	56 (0.05)	98 (0.04)
Witness status:			
Witnessed	297 826 (85.3)	93 613 (81.1)	204 213 (87.4)
Unwitnessed	51 170 (14.7)	21 832 (18.9)	29 338 (12.6)
First documented rhythm,:			
Asystole	99 933 (28.6)	40 971 (35.5)	58 962 (25.2)
Pulseless electrical activity	167 779 (48.1)	54 178 (46.9)	113 601 (48.6)
Ventricular fibrillation	33 100 (9.5)	9097 (7.9)	24 003 (10.3)
Pulseless ventricular tachycardia	23 337 (6.7)	5164 (4.5)	18 173 (7.8)
Unknown	24 847 (7.1)	6035 (5.2)	18 812 (8.1)
Epinephrine administration	310 166 (88.9)	111 804 (96.8)	198 362 (84.9)
Median (IQR) interval between start of chest compression and first epinephrine administration, minutes	1 (0-3)	2 (0-4)	1 (0-3)
Endotracheal intubation	174 504 (50.0)	61 100 (52.9)	113 404 (48.6)
Median (IQR) interval between start of chest compression and endotracheal intubation, , minutes	5 (2-9)	5 (2-9)	5 (2-9)

IQR=interquartile range; ROSC=return of spontaneous circulation.

* Ambulatory and outpatient areas; delivery suite; rehabilitation, skilled nursing, and mental health facilities; same day surgical areas; and “other.”

### Cumulative proportion of patients achieving first return of spontaneous circulation over time stratified by outcomes

**Fig 2:**
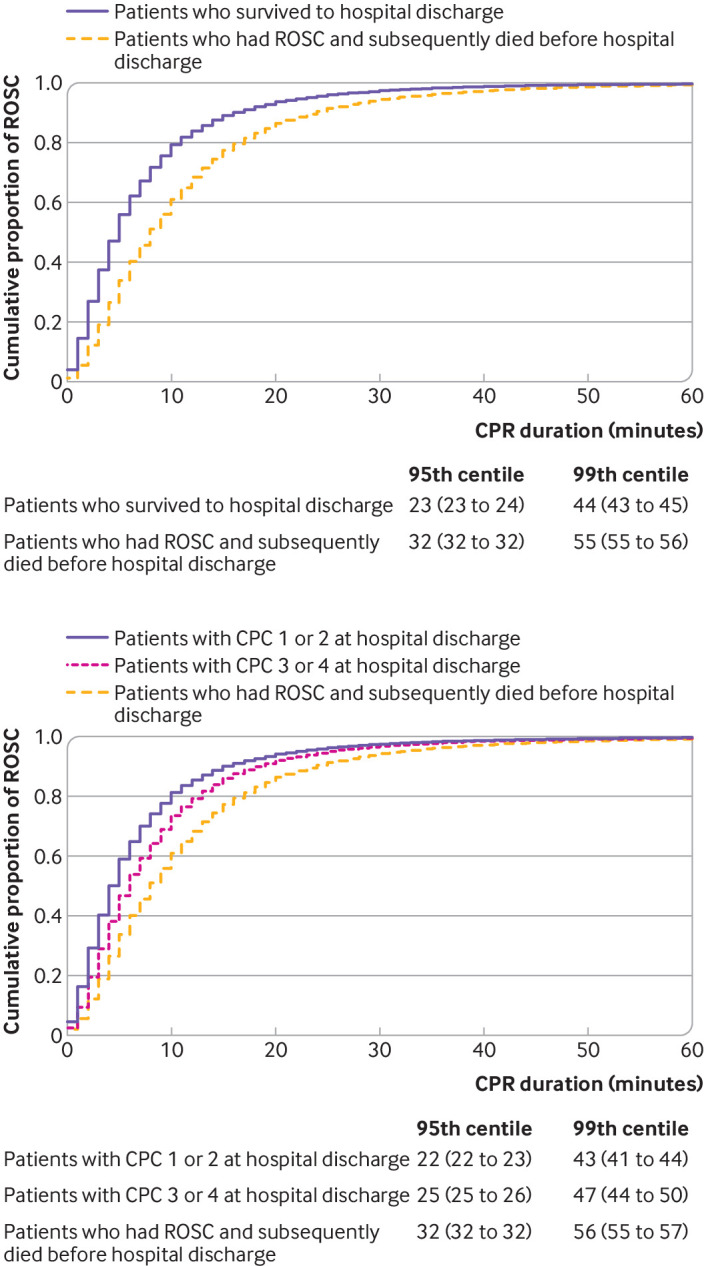
Cumulative proportion of patients achieving first return of spontaneous circulation (ROSC), stratified by patients with outcomes: survival to hospital discharge (top) and functional outcome at hospital discharge (bottom). CI=confidence interval; CPC=cerebral performance category; CPR=cardiopulmonary resuscitation

Figure 2 shows the cumulative proportion of patients achieving the first return of spontaneous circulation, stratified by patients’ outcomes. Almost all (99%) patients who survived to hospital discharge had the first return of spontaneous circulation within 44 (95% confidence interval 43 to 45) minutes’ duration of CPR (fig 2, top). Almost all (99%) patients who had favorable functional outcome at hospital discharge had the first return of spontaneous circulation within 43 (41 to 44) minutes (fig 2, bottom).

### Time dependent probabilities of outcomes among patients pending first return of spontaneous circulation at each minute’s duration of CPR

**Fig 3:**
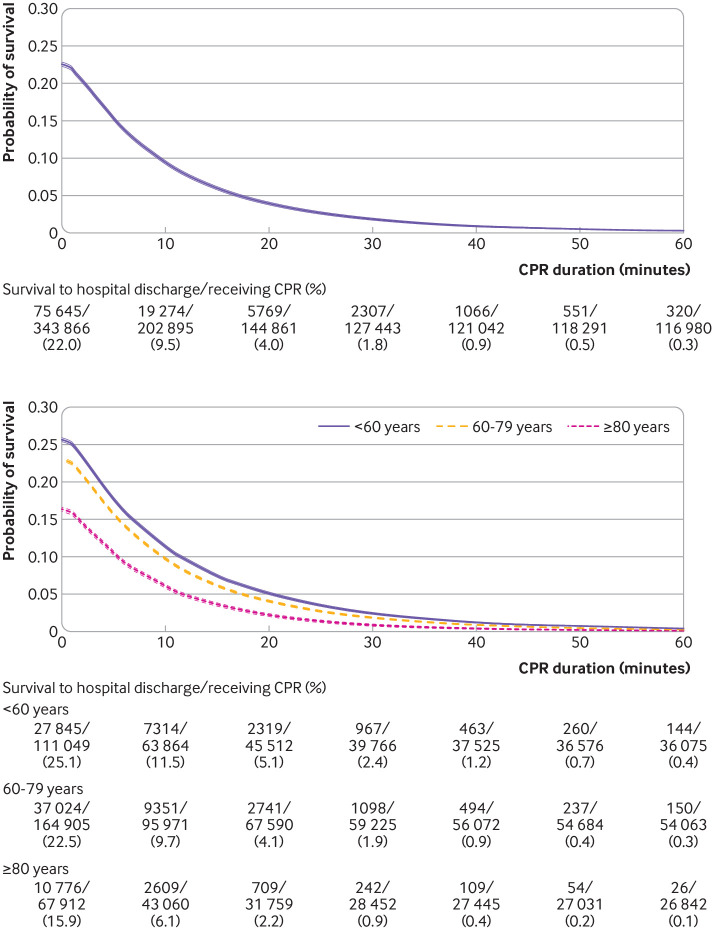
Time dependent probability of survival to hospital discharge with 95% confidence intervals among patients pending first return of spontaneous circulation at each time point for all patients (top) and stratified by age group (bottom). Denominators included patients who were undergoing cardiopulmonary resuscitation (CPR) pending first return of spontaneous circulation and patients who had termination of resuscitation before or at each time point

**Fig 4:**
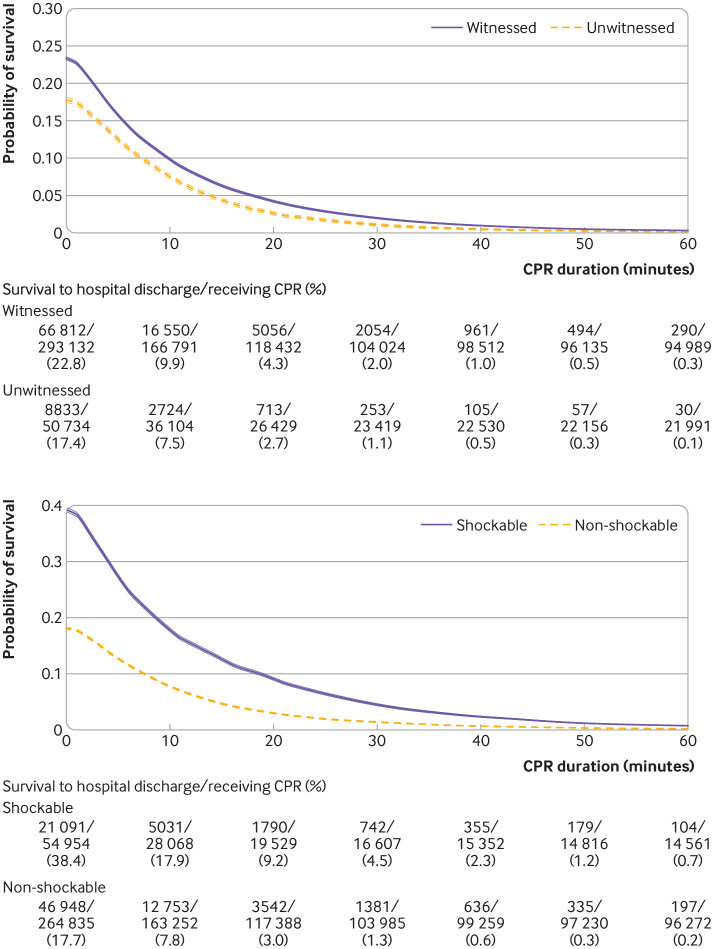
Time dependent probability of survival to hospital discharge with 95% confidence intervals among patients pending first return of spontaneous circulation at each time point, stratified by witness status (top) and initial rhythm (bottom). Denominators included patients who were undergoing cardiopulmonary resuscitation (CPR) pending first return of spontaneous circulation and patients who had termination of resuscitation before or at each time point

**Fig 5:**
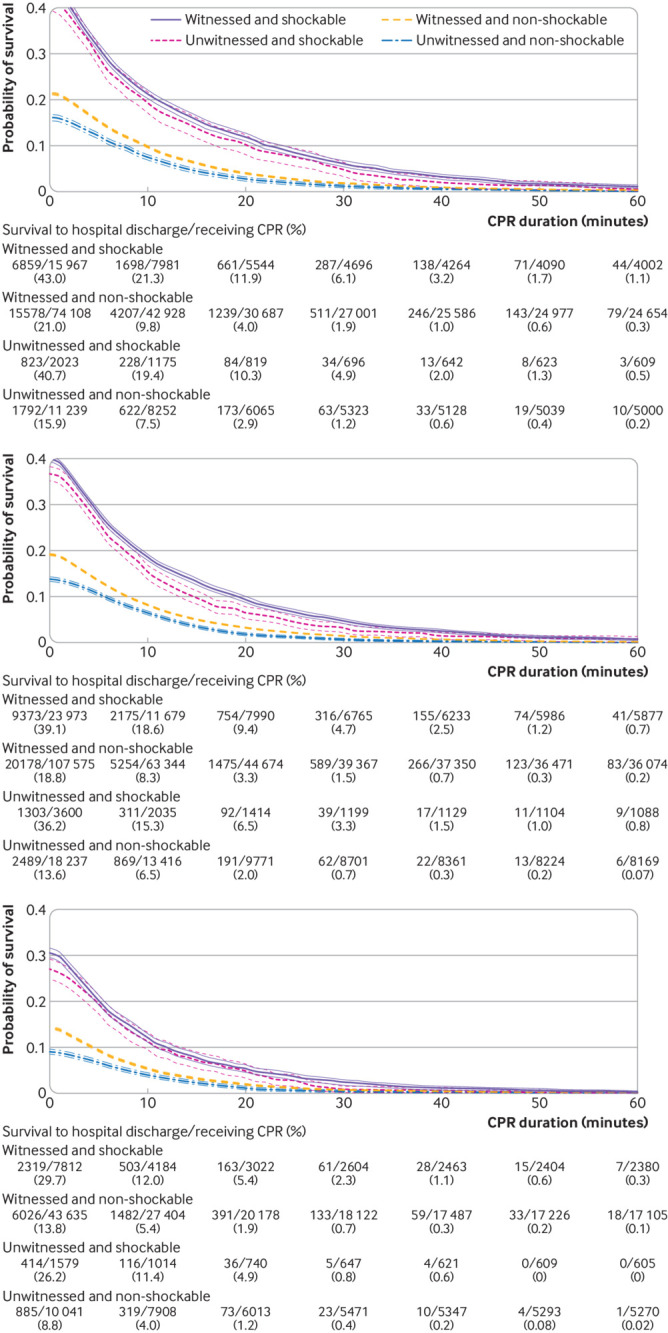
Time dependent probability of survival to hospital discharge with 95% confidence intervals among patients pending first return of spontaneous circulation at each time point. Combination of witness status and initial rhythm among age <60 years (top), age 60-79 years (middle), and age ≥80 years (bottom). Denominators included patients who were undergoing cardiopulmonary resuscitation (CPR) pending first return of spontaneous circulation and patients who had termination of resuscitation before or at each time point

**Fig 6:**
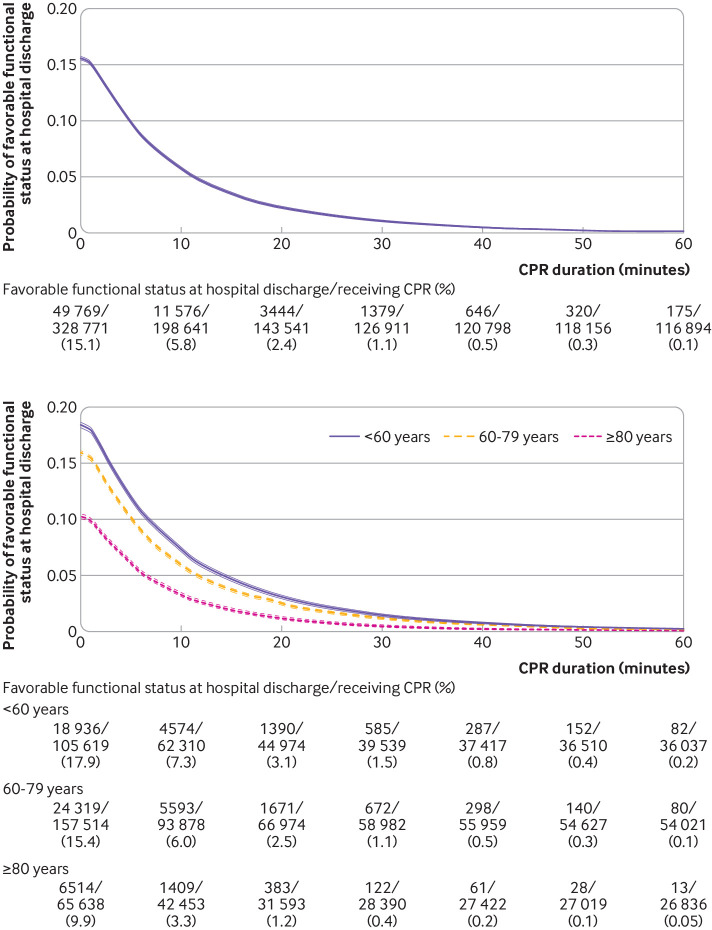
Time dependent probability of favorable functional outcome at hospital discharge with 95% confidence intervals among patients pending first return of spontaneous circulation at each time point for all patients (top) and stratified by age group (bottom). Denominators included patients who were undergoing cardiopulmonary resuscitation (CPR) pending first return of spontaneous circulation and patients who had termination of resuscitation before or at each time point

**Fig 7:**
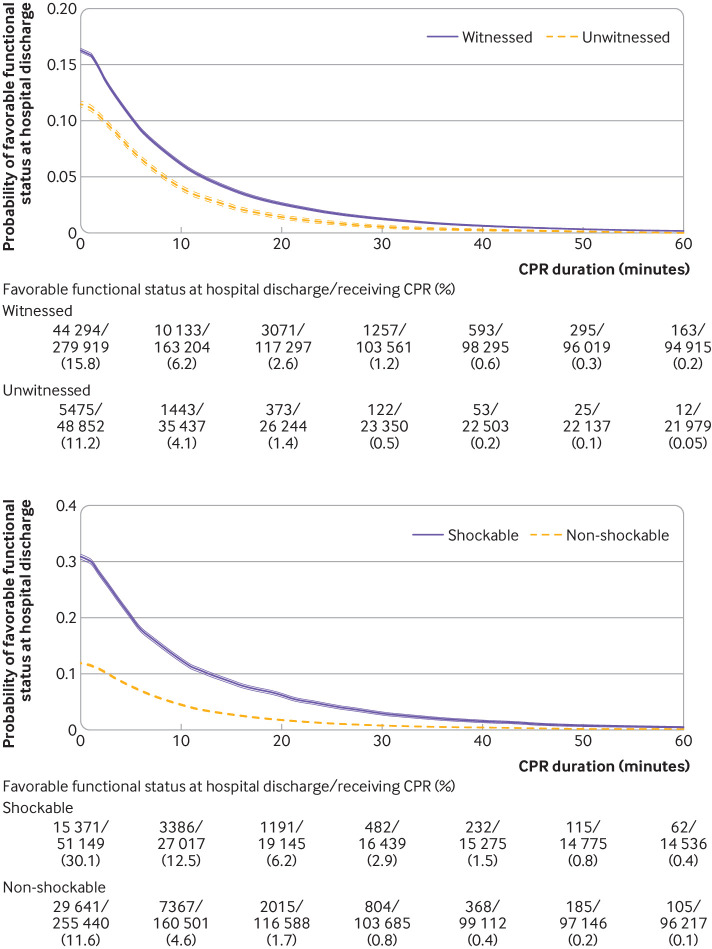
Time dependent probability of favorable functional outcome at hospital discharge with 95% confidence intervals among patients pending first return of spontaneous circulation at each time point, stratified by witness status (top) and initial rhythm (bottom). Denominators included patients who were undergoing cardiopulmonary resuscitation (CPR) pending first return of spontaneous circulation and patients who had termination of resuscitation before or at each time point

**Fig 8:**
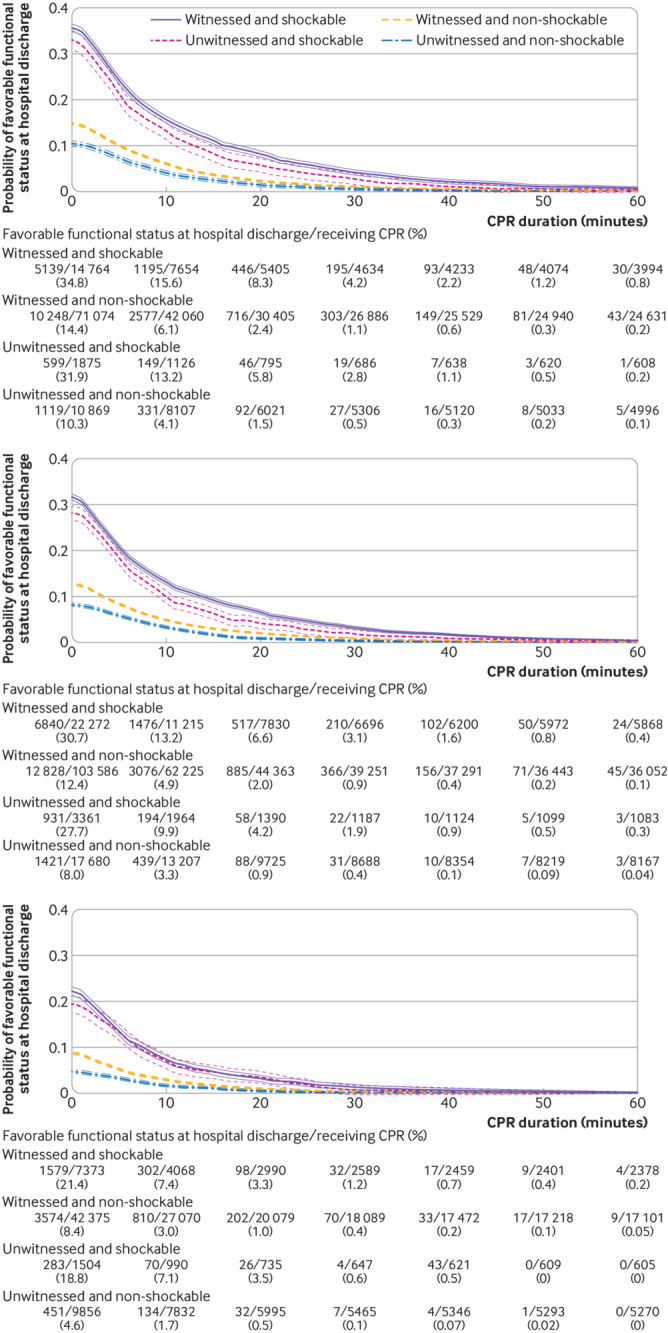
Time dependent probability of favorable functional outcome at hospital discharge with 95% confidence intervals among patients pending first return of spontaneous circulation at each time point. Combination of witness status and initial rhythm among age <60 years (top), age 60-79 years (middle), and age ≥80 years (bottom). Denominators included patients who were undergoing cardiopulmonary resuscitation (CPR) pending first return of spontaneous circulation and patients who had termination of resuscitation before or at each time point

We present time dependent probabilities of survival to hospital discharge (fig 3, fig 4, and fig 5) and favorable functional outcome at hospital discharge (fig 6, fig 7, and fig 8) among patients pending the first return of spontaneous circulation at each minute’s duration of cardiopulmonary resuscitation for overall patients and each clinical feature and patient phenotype, using the denominator including patients who had termination of resuscitation before or at each time point (primary analysis). Among overall patients with in-hospital cardiac arrest, the probabilities of survival and favorable functional outcome among those pending the first return of spontaneous circulation at 1 minute’s duration of cardiopulmonary resuscitation were 22.0% and 15.1%, respectively (fig 3, top; fig 6, top). As duration of cardiopulmonary resuscitation increased, the probabilities of survival and favorable functional outcome decreased and plateaued at 0.3-0.9% and 0.1-0.5% between 40 minutes and 60 minutes, respectively. The probabilities of survival at 39 minutes’ and favorable functional status at 32 minutes’ duration of cardiopulmonary resuscitation were less than 1% (supplementary table B). In terms of clinical features, age younger than 60 years, witnessed arrest, and initial shockable rhythm showed the higher estimates of the time dependent probabilities of survival (fig 3, bottom; fig 4) and favorable functional outcome (fig 6, bottom; fig 7). Across clinical features, stratification by initial rhythm showed the largest change in the time dependent probabilities for each outcome (fig 4, bottomt; fig 7, bottom), and shockable rhythm had the longest duration of cardiopulmonary resuscitation before the time dependent probabilities of survival and functional outcome became less than 1% (supplementary table B). Figure 5 shows time dependent probabilities of survival and figure 8 shows time dependent probabilities of favorable functional outcome for patient phenotypes. Across patient phenotypes, patients who were younger than 60 years, had witnessed arrest, and had initial shockable rhythm showed the highest point estimates of the time dependent probabilities of survival (fig 5) and favorable functional outcome (fig 8) and the longest duration of cardiopulmonary resuscitation before the time dependent probabilities became less than 1% (supplementary table B).

Supplementary figures B and C show the time dependent probabilities of survival and favorable functional outcome among patients pending the first return of spontaneous circulation at each minute’s duration of cardiopulmonary resuscitation, using the denominator excluding patients who had termination of resuscitation before or at each time point (sensitivity analysis). Among overall patients with in-hospital cardiac arrest, the time dependent probabilities of survival and favorable functional outcome among patients pending the first return of spontaneous circulation at 1 minute’s duration of cardiopulmonary resuscitation were 22.0% and 15.2%, respectively. As duration of cardiopulmonary resuscitation increased from 1 to 20 minutes, the probabilities decreased but then plateaued at 5.4-6.3% for survival and 3.2-3.8% for favorable functional outcome between 20 minutes and 60 minutes.

### Time dependent probabilities of outcomes among patients who had first return of spontaneous circulation before or at each minute’s duration of cardiopulmonary resuscitation

In the supplement, we present time dependent probabilities of survival (figure D) and favorable functional outcome (figure E) among patients who had the first return of spontaneous circulation before or at each time point for overall patients and for each clinical feature and patient phenotype. Among clinical features, younger age group and initial shockable rhythm consistently showed higher time dependent probability of survival (supplementary figures D2 and D4) and favorable functional outcome (supplementary figures E2 and E4), and witness status showed crossover of the probability for each outcome (supplementary figures D3 and E3).

### Subgroup analysis

The subgroup analysis including the subset of patients with in-hospital cardiac arrest between 2011 and 2021 is shown in supplementary figures F-L and table B. Across most of the clinical features and patient phenotypes, the durations of cardiopulmonary resuscitation before the time dependent probabilities of survival and functional outcome among patients pending the first return of spontaneous circulation before or at each time point became less than 1% were longer in the subgroup of patients between 2011 and 2021 than in the overall study population between 2000 and 2021 (supplementary table B).

## Discussion

In this analysis of a large multicenter prospective registry of in-hospital cardiac arrest in the United States between 2000 and 2021, we quantified the time dependent probabilities of survival to hospital discharge and favorable functional outcome at hospital discharge as a function of duration of cardiopulmonary resuscitation. We found that 99% of patients who eventually survived to hospital discharge and who had favorable functional outcome at hospital discharge achieved the first return of spontaneous circulation within 44 minutes’ and 43 minutes’ duration of cardiopulmonary resuscitation, respectively. The time dependent probabilities of survival and favorable functional outcome among patients pending the first return of spontaneous circulation at each minute’s duration of cardiopulmonary resuscitation using the denominator that included patients who had termination of resuscitation before or at each time point decreased and plateaued at less than 1% as duration of cardiopulmonary resuscitation increased beyond 39 minutes and 32 minutes, respectively. By contrast, the time dependent probabilities using the denominator that excluded patients who had termination of resuscitation before or at each time point plateaued above 5% for survival and 3% for favorable functional outcome as duration of cardiopulmonary resuscitation increased.

### Comparison with other studies

A previous observational study using the GWTG-R registry showed that duration of resuscitative efforts before termination of resuscitation varied across hospitals, and resuscitation at hospitals with longer resuscitative efforts before termination of resuscitation was associated with an increased chance of survival to hospital discharge.^24^ Another study using the GWTG-R registry reported that patients with higher predicted survival had longer duration of resuscitative efforts before termination of resuscitation.^25^ However, scarce previous work has evaluated the association between duration of cardiopulmonary resuscitation at the patient level and patients’ outcomes after in-hospital cardiac arrest. A systematic review and meta-analysis in 2019 evaluating pre-arrest and intra-arrest prognostic factors for in-hospital cardiac arrest included only one published study from 1995 and one unpublished study between 2011 and 2018 from the United Kingdom National Cardiac Arrest Audit (UK NCAA) to evaluate the association between duration of cardiopulmonary resuscitation and survival.^26^ ^27^ The systematic review and meta-analysis reported that duration of cardiopulmonary resuscitation of more than 15 minutes was associated with decreased odds of survival (adjusted odds ratio 0.06 (95% confidence interval 0.02 to 0.21); adjusted odds ratio 0.13 (0.12 to 0.14) for the UK NCAA data). A Swedish observational study published in 2018 showed the association between longer quarters of duration of cardiopulmonary resuscitation and decreased chance of 30 day survival (adjusted odds ratio 0.69 (0.37 to 1.29) for quarter 2 (duration 3-5 minutes); 0.35 (0.19 to 0.65) for quarter 3 (duration 6-12 minutes); 0.10 (0.05 to 0.20) for quarter 4 (duration ≥13 minutes)), compared with quarter 1 (duration <2 minutes).^28^ A recent observational study of 8727 patients with in-hospital cardiac arrest in 2022, using a national in-hospital cardiac arrest registry in Denmark, reported the association between duration of cardiopulmonary resuscitation and 30 day survival rate (62.0% for quarter 1 (duration <5 minutes), 32.7% for quarter 2 (duration 5-11 minutes), 14.4% for quarter 3 (duration 12-20 minutes), and 8.1% for quarter 4 (duration ≥21 minutes)).^29^ These previous studies have several methodological limitations. The duration of cardiopulmonary resuscitation was treated as a categorical variable, and odds ratios were reported assuming a linear relation between duration of cardiopulmonary resuscitation and outcomes. Avoiding categorizing a continuous variable is recommended, as this can result in loss of information and is rarely justifiable, compared with analyzing the continuous variable on its continuous scale.^30^ ^31^ In addition, our results indicate that duration of cardiopulmonary resuscitation and probabilities of favorable outcomes do not show a linear relation. Our findings extend our understanding of the relation between duration of cardiopulmonary resuscitation and outcomes by quantifying time dependent probabilities of subsequent outcomes when patients did not have the first return of spontaneous circulation and when patients achieved the first return of spontaneous circulation in each minute’s duration of cardiopulmonary resuscitation.

In comparison with out-of-hospital cardiac arrest, a retrospective analysis of a multicenter, cluster randomized clinical trial (ROC-PRIMED; Resuscitation Outcomes Consortium Prehospital Resuscitation Using an Impedance Valve and Early Versus Delayed) in the US and Canada showed that 99% of patients who had favorable functional outcome at hospital discharge had return of spontaneous circulation within 37 minutes’ duration of cardiopulmonary resuscitation.^10^ This is shorter than 43 minutes in our results, which is likely explained by the difference in time to start of cardiopulmonary resuscitation in out-of-hospital and in-hospital settings.

### Implications of findings

Firstly, we quantified the time dependent changes in the probabilities of patients’ outcomes as a function of duration of cardiopulmonary resuscitation. The time dependent probabilities of survival and favorable functional outcome among patients pending the first return of spontaneous circulation at each minute’s duration of cardiopulmonary resuscitation provide resuscitation teams, patients, and their surrogates with insights into the likelihood of favorable outcomes if the patient continues to receive cardiopulmonary resuscitation beyond that time point, which is clinically informative for shared decision making to determine whether further cardiopulmonary resuscitation would be beneficial. For example, a pamphlet describing this time dependent probabilities would be helpful for patients’ surrogates to provide objective guidance and make decisions on continuing or discontinuing cardiopulmonary resuscitation. On the other hand, the probabilities of survival and favorable functional outcome among patients who had the first return of spontaneous circulation before or at each time point inform resuscitation teams, patients, and their surrogates of the likelihood of favorable outcomes once the patient has achieved return of spontaneous circulation at or by the time point, which is clinically relevant to estimate subsequent outcomes after return of spontaneous circulation.

Secondly, we observed two distinct features of time dependent probabilities of survival and favorable functional outcome among patients pending the first return of spontaneous circulation at each minute’s duration of cardiopulmonary resuscitation, depending on the denominators used. Notably, these two probabilities are different in two different populations. When the denominator included patients who had termination of resuscitation before or at each time point, the time dependent probabilities of survival and favorable functional outcome decreased and plateaued below traditional medical futility, the likelihood of survival of less than 1% as patients received longer duration of cardiopulmonary resuscitation.^21^ ^22^ By contrast, using the denominator that excluded patients who had termination of resuscitation before or at each time point, the probabilities of survival and favorable functional outcome plateaued above 5% and above 3% respectively after 20 minutes’ cardiopulmonary resuscitation. This difference could be probably explained by two factors: self-fulfilling prophecy—the treating team used duration of cardiopulmonary resuscitation for decisions to terminate resuscitative efforts—and confounding by indication—only a subset of patients for whom the treating providers believed that prolonged cardiopulmonary resuscitation could be beneficial had prolonged cardiopulmonary resuscitation. Therefore, only highly selected patients were included in the denominator when patients who had termination of resuscitation were excluded. These findings would imply that the decision to terminate resuscitation should not be solely dependent on duration of cardiopulmonary resuscitation (for example, time points of <1% of survival in supplementary table B) but should be based on clinical judgment of treating providers.

Thirdly, given the time dependent probabilities of outcomes among patients pending the first return of spontaneous circulation at each minute’s duration of cardiopulmonary resuscitation across the clinical features and patient phenotypes, in-hospital cardiac arrest with younger age, witnessed, and with initial shockable rhythm would benefit from longer duration of cardiopulmonary resuscitation than those with older age, unwitnessed, and with initially non-shockable rhythm.

Fourthly, in the subgroup analysis that included patients with in-hospital cardiac arrest between 2011 and 2021, we found that, for most of the clinical features and patient phenotypes, the durations of cardiopulmonary resuscitation before the time dependent probabilities of survival and favorable functional outcome among patients pending the first return of spontaneous circulation at each time point with the denominators that included termination of resuscitation became less than 1% were longer than those of the overall study population between 2000 and 2021 (supplementary table B). This may be due to improved post-resuscitation care in the 2011-21 time period.^4^ ^32^ This suggests that outcome rates in the study population affect the time dependent probability. As variations in outcomes across participating hospitals in the GWTG-R are known,^33^ the time dependent probability of survival and favorable functional outcome among patients pending the first return of spontaneous circulation at each time point may vary across the hospitals. Our results should be interpreted as the average time dependent probability of outcomes in the dataset, and the probabilities may not be the same at each hospital.

### Unanswered questions and future research

The stratified time dependent probabilities of survival and favorable functional outcome among patients pending the first return of spontaneous circulation at each time point by clinical features and patient phenotypes provided information about who could benefit from prolonged cardiopulmonary resuscitation, as the probabilities of favorable outcomes differed across features and phenotypes. However, other factors in addition to clinical features and patient phenotypes may also be determinants of the outcomes, and further work is warranted to understand such factors that could justify prolonged cardiopulmonary resuscitation.

In our study, the median interval between start of chest compression and termination of resuscitation was 20 (interquartile range 14-30) minutes among patients without return of spontaneous circulation, whereas we found that the probabilities of survival to hospital discharge and favorable functional outcome become less than 1% at 39 minutes’ and 32 minutes’ duration of cardiopulmonary resuscitation, respectively. Most termination of resuscitation occurred before the time point of traditional medical futility. Further research is needed to evaluate whether patients’ outcomes would improve with prolonged cardiopulmonary resuscitation before termination of resuscitation. Our results might generate a clinical equipoise that justifies a future clinical trial to compare a resuscitation strategy with duration of cardiopulmonary resuscitation at providers’ discretion before termination of resuscitation (a usual care group) versus a resuscitation strategy with prespecified duration of cardiopulmonary resuscitation before termination of resuscitation (an intervention group) for patients with in-hospital cardiac arrest.

Extracorporeal cardiopulmonary resuscitation is an advanced rescue therapy to support circulation in selected patients with refractory cardiac arrest, by an implantation of venoarterial extracorporeal membrane oxygenation.^34^ A recent meta-analysis showed that extracorporeal cardiopulmonary resuscitation for patients with in-hospital cardiac arrest was associated with lower in-hospital morality.^35^ As our study results showed that the probabilities of favorable outcomes decreased as duration of cardiopulmonary resuscitation increased, future research is needed to assess the optimal timing and patient selection to start extracorporeal cardiopulmonary resuscitation when cardiac arrest is refractory to conventional cardiopulmonary resuscitation in hospitals where extracorporeal cardiopulmonary resuscitation is available.

### Strengths and limitations of study

Using the largest in-hospital cardiac arrest dataset in the world, we explicitly examined and quantified the changes in the probabilities of favorable outcomes for both patients undergoing cardiopulmonary resuscitation pending the first return of spontaneous circulation at a given moment and those who have achieved the first return of spontaneous circulation before or at the time point.

Our study has several limitations. Firstly, we used two definitions for the denominator of time dependent probability of survival and favorable functional outcome among patients pending the first return of spontaneous circulation at each time point. The denominator that included patients who were undergoing cardiopulmonary resuscitation and patients who had termination of resuscitation relied on the assumption that all termination of resuscitation was appropriate. However, proving this assumption is not possible. In the denominator excluding those with termination of resuscitation, outcomes of patients who had termination of resuscitation without return of spontaneous circulation were censored. As duration of cardiopulmonary resuscitation is associated with patients’ outcomes, the decision to terminate resuscitative efforts were likely affected by duration of cardiopulmonary resuscitation (self-fulfilling prophecy). Secondly, collecting time variables during cardiopulmonary resuscitation is difficult, and the precision of the collected time variables is an important limitation. However, the use of a large in-hospital cardiac arrest registry with standardized data definitions and data collection systems was intended to minimize this limitation. Thirdly, we were unable to account for the severity of underlying pre-arrest comorbidities, which could have been one of factors when termination of resuscitation was decided. However, we did multiple stratified analyses by clinical features and patient phenotypes to account for the potential confounders. Fourthly, unmeasured quality of cardiopulmonary resuscitation (for example, chest compression metrics) or post-resuscitation care might be different across treating teams, and such factors might be correlated with duration of cardiopulmonary resuscitation and patients’ outcomes. Lastly, the generalizability of our findings is an important limitation as the GWTG-R is a voluntary registry and participation in the registry may reflect the interest in quality improvement of resuscitation of each hospital. Conversely, our findings are pertinent for programs intending to improve their resuscitation performance.

### Conclusions

In this analysis of a large multicenter prospective registry of in-hospital cardiac arrest between 2000 and 2021 in the United States, we quantified the time dependent probabilities of survival to hospital discharge and favorable functional outcome at hospital discharge as a function of duration of cardiopulmonary resuscitation. The findings provide resuscitation teams, patients, and their surrogates with the objective probabilities of subsequent favorable outcomes if patients pending the first return of spontaneous circulation received further cardiopulmonary resuscitation.

## What is already known on this topic

Longer duration of resuscitation for patients with in-hospital cardiac arrest is associated with decreased likelihood of survival

## What this study adds

The time dependent probabilities of two outcomes among patients pending the first return of spontaneous circulation were quantified in each minute of cardiopulmonary resuscitation (CPR) duration
The outcomes assessed were survival to hospital discharge and favorable functional outcome at hospital discharge
The time dependent probabilities of survival and favorable functional status rapidly declined and were less than 1% at 39 minutes and at 32 minutes of CPR duration, respectively

## Data Availability

No additional data available.

## Web extra

Extra material supplied by authors

Web appendix: Supplementary materials

## Notes

Contributors: MO and SK contributed to the design and conduct of the study, data collection and management, and analysis of the data. MO drafted the manuscript. All authors contributed to the interpretation of the data and presentation, review, and critical revision and approval of the manuscript. MO is the guarantor. The corresponding author attests that all listed authors meet authorship criteria and that no others meeting the criteria have been omitted.

AHA GWTG-R Adult Research Task Force members: Anne Grossestreuer, Ari Moskowitz, Dana Edelson, Joseph Ornato, Mary Ann Peberdy, Matthew Churpek, Monique Anderson Starks, Paul Chan, Saket Girotra, Sarah Perman, Zachary Goldberger

Transparency statement: The lead author (the manuscript’s guarantor) affirms that this manuscript is an honest, accurate, and transparent account of the study being reported; that no important aspects of the study have been omitted; and that any discrepancies from the study as planned (and, if relevant, registered) have been explained.

Dissemination to participants and related patient and public communities: The research findings will be disseminated through press releases, interviews with local and national media, social media posts on Twitter/X, and academic conferences.

Provenance and peer review: Not commissioned; externally peer reviewed.
