# Supplementary material for: Duration of cardiopulmonary resuscitation and outcomes for adults with in-hospital cardiac arrest: retrospective cohort study
Source: BMJ. 2024 Feb 7;384:e076019. doi: 10.1136/bmj-2023-076019 (PMC10847985; doi:10.1136/bmj-2023-076019)
Supplement: Supplementary file 1 — Web appendix: Supplementary materials [file okum076019.ww1.pdf]

Supplementary Online Content

Supplemental Methods

Supplemental Table A. Patient and cardiac arrest characteristics between patients with and without missing cardiopulmonary resuscitation duration, survival to hospital discharge, or functional outcome at hospital discharge

Supplemental Table B. Resuscitation duration when point estimates of time-dependent probabilities of outcomes among those pending the first return of spontaneous circulation, using the denominator including patients who had termination of resuscitation prior to or each minute, become less than 1%.

Supplemental Figure A. Time course of cardiopulmonary resuscitation and time-dependent probabilities.

All patients who received CPR had either ROSC or TOR. Patients with ROSC survived to hospital discharge or died before hospital discharge. Those who survived to hospital discharge always had ROSC, and ROSC is a mediator of survival to hospital discharge.

- Time-dependent probability of survival among those pending the first ROSC at 21 minutes, using the denominator that included patients who had TOR (primary analysis) is (Patient B) / (Patient A + Patient B + Patient C + Patient D) = 1/4 = 25.0%.

- Time-dependent probability of survival among those pending the first ROSC at 21 minutes, using the denominator that excluded patients who had TOR (sensitivity analysis) is (Patient B) / (Patient B + Patient C + Patient D) = 1/3 = 33.3%. This denominator treated TOR before 21 minutes (Patient A) as a censoring event.
- Time-dependent probability of survival among those who had the first ROSC prior to or at 22 minutes is (Patient B + Patient E) / (Patient B + Patient D + Patient E) = 2/3 = 66.7%.

CPR indicates cardiopulmonary resuscitation; ROSC, return of spontaneous circulation; TOR, termination of resuscitation.

Supplemental Figure B. Time-dependent probability of survival to hospital discharge (solid line) with 95% confidence intervals (dash lines) among those pending the first return of spontaneous circulation at each time point. All patients (1). Stratification by age group (2), witnessed status (3), and initial rhythm (4). Combination of witnessed status and initial rhythm among age<60 years (5), age 60-79 years (6), and age≥80 years (7). The denominators included patients who were undergoing cardiopulmonary resuscitation pending the first return of spontaneous circulation, and patients who had termination of resuscitation prior to or at each time point were censored.

Supplemental Figure C. Time-dependent probability of favorable functional outcome at hospital discharge (solid line) with 95% confidence intervals (dash lines) among those pending the first return of spontaneous circulation at each time point. All patients (1). Stratification by age group (2), witnessed status (3), and initial rhythm (4). Combination of witnessed status and initial rhythm among age<60 years (5), age 60-79 years (6), and age≥80 years (7). The

denominators included patients who were undergoing cardiopulmonary resuscitation pending the first return of spontaneous circulation, and patients who had termination of resuscitation prior to or at each time point were censored.

Supplemental Figure D. Time-dependent probability of survival to hospital discharge (solid line) with 95% confidence interval (dash lines) among those with the first return of spontaneous circulation prior to or at each time point. All patients (1). Stratification by age group (2), witnessed status (3), and initial rhythm (4). Combination of witnessed status and initial rhythm among age<60 years (5), age 60-79 years (6), and age≥80 years (7).

Supplemental Figure E. Time-dependent probability of favorable functional outcome at hospital discharge (solid line) with 95% confidence interval (dash lines) among those with the first return of spontaneous circulation prior to or at each time point. All patients (1). Stratification by age group (2), witnessed status (3), and initial rhythm (4). Combination of witnessed status and initial rhythm among age<60 years (5), age 60-79 years (6), and age≥80 years (7).

Supplemental Figure F. Cumulative proportion of patients achieving the first return of spontaneous circulation between 2011 and 2021, stratified by survival to hospital discharge (1) and functional outcome at hospital discharge (2).

CI indicates confidence interval; CPC, cerebral performance category; CPR, cardiopulmonary resuscitation; ROSC, return of spontaneous circulation.

Supplemental Figure G. Time-dependent probability of survival to hospital discharge (solid line) with 95% confidence intervals (dash lines) among those pending the first return of spontaneous circulation at each time point between 2011 and 2021. The denominators included patients who were undergoing cardiopulmonary resuscitation pending the first return of spontaneous circulation and patients who had termination of resuscitation prior to or at each time point.

Supplemental Figure H. Time-dependent probability of favorable functional outcome at hospital discharge (solid line) with 95% confidence intervals (dash lines) among those pending the first return of spontaneous circulation at each time point between 2011 and 2021. The denominators included patients who were undergoing cardiopulmonary resuscitation pending the first return of spontaneous circulation and patients who had termination of resuscitation prior to or at each time point.

Supplemental Figure I. Time-dependent probability of survival to hospital discharge (solid line) with 95% confidence intervals (dash lines) among those pending the first return of spontaneous circulation at each time point between 2011 and 2021. The denominators included patients who were undergoing cardiopulmonary resuscitation pending the first return of spontaneous circulation, and patients who had termination of resuscitation prior to or at each time point were censored.

Supplemental Figure J. Time-dependent probability of favorable functional outcome at hospital discharge (solid line) with 95% confidence intervals (dash lines) among those pending the first

return of spontaneous circulation at each time point between 2011 and 2021. The denominators included patients who were undergoing cardiopulmonary resuscitation pending the first return of spontaneous circulation, and patients who had termination of resuscitation prior to or at each time point were censored.

Supplemental Figure K. Time-dependent probability of survival to hospital discharge (solid line) with 95% confidence interval (dash lines) among those with the first return of spontaneous circulation prior to or at each time point between 2011 and 2021.

Supplemental Figure L. Time-dependent probability of favorable functional outcome at hospital discharge (solid line) with 95% confidence interval (dash lines) among those with the first return of spontaneous circulation prior to or at each time point between 2011 and 2021.

Supplemental Methods

Time-dependent probabilities notations **(**Supplemental Figure A)

1. *Time-dependent probabilities of outcomes among those pending the first return of spontaneous circulation (ROSC) at each minute of cardiopulmonary resuscitation (CPR) duration*

   1.1. Primary analysis: The denominator included patients who had termination of resuscitation (TOR) prior to or at each minute of CPR duration.

   PS_t is the probability of having the outcome if CPR continued beyond time t and ROSC was not achieved by time point t.

   $PS_t = P(S = 1 \mid T > t) = P(S = 1 \text{ AND } T > t) / P(T>t)$

   Where,

   T = CPR duration; $t_0 < T < t_{max}$; if ROSC, then T = time to ROSC; if TOR, then T = t_{max} since CPR was assumed to be continued till t_{max} for those with TOR.

   S = Survival to discharge (0 = died, 1 = survived)

   t = time since start of CPR

   t_0 = start of CPR

   t_{max} = maximum duration of CPR

   Using frequency to estimate this probability

   Numerator: $P(S = 1 \text{ AND } T > t) = \#\{\text{patients with } T > t \text{ AND } S = 1\}$

   Denominator: $P(T>t) = \#\{\text{patients with } T > t\}$

1.2. Sensitivity analysis: The denominator excluded patients who had termination of resuscitation (TOR) prior to or at each minute of CPR duration.

$PS\_t = P(S = 1 \mid T > t) = P(S = 1 \text{ AND } T > t) / P(T>t)$

Where,

T =CPR duration; $t\_0 < T < t\_max$; if ROSC, then T=time to ROSC; if TOR, then T = time to TOR

S = Survival to discharge (0 = died, 1 = survived)

t = time since start of CPR

t_0 = start of CPR

t_max = maximum duration of CPR

Using frequency to estimate this probability

Numerator: $P(S = 1 \text{ AND } T > t)$ = # {patients with T > t AND S = 1}

Denominator: $P(T>t)$ = # {patients with T > t}

2. *Time-dependent probabilities of outcomes among those who had the first ROSC prior to or at each minute of CPR duration*

PS_t is the probability of having the outcome if the first ROSC was achieved prior to or at time point t.

$PS\_t = P(S = 1 \mid T \leq t) = P(S = 1 \text{ AND } T \leq t) / P(T \leq t)$

Where,

T = time to the first ROSC; $t\_0 < T < t\_max$

S = Survival to discharge (0 = died, 1 = survived)

t = time since start of CPR

t_0 = start of CPR

t_{max} = maximum duration of CPR

Using frequency to estimate this probability

Numerator: $P(S = 1 \text{ AND } T \leq t)$ = # {patients with $T \leq t$ AND $S = 1$}

Denominator: $P(T \leq t)$ = # {patients with $T \leq t$}

Supplemental Table A. Patient and cardiac arrest characteristics between patients with and without missing cardiopulmonary resuscitation duration, survival to hospital discharge, or functional outcome at hospital discharge

	Patients without missing variables	Patients with missing variables	Standardized mean difference
Number of patients, n (%)	333,351	58,133	
CPR duration missing	0	40,059 (68.9)	
Survival to hospital discharge missing	0	2,429 (4.2)	
Functional outcome missing	0	15,645 (26.9)	
**Patient Characteristics**			
Age, median (IQR), y	67 (56-77)	66 (55-76)	0.051
Sex, n (%)			0.018
Male	194,682 (58.4)	33,494 (57.6)	
Female	138,642 (41.6)	24,629 (42.4)	
Unknown	27 (0.0)	10 (0.0)	
Race, n (%)			0.204
White	228,116 (68.4)	36,460 (62.7)	
Black	73,560 (22.1)	13,116 (22.6)	
Other[a]	10,895 (3.3)	1,712 (2.9)	
Unknown	20,780 (6.2)	6,845 (11.8)	
Illness category, n (%)			0.149
Medical			
Cardiac	116,996 (35.1)	21,614 (37.2)	
Non-cardiac	148,356 (44.5)	22,975 (39.5)	
Surgical			
Cardiac	19,907 (6.0)	4,679 (8.0)	
Non-cardiac	35,042 (10.5)	6,479 (11.1)	
Trauma	11,802 (3.5)	1,786 (3.1)	

Other[b]	996 (0.3)	311 (0.5)	
Unknown	252 (0.1)	289 (0.5)	
Preexisting condition, n (%)			
Cardiac			
History of myocardial infarction	46,502 (13.9)	7,281 (12.5)	0.054
Myocardial infarction, this admission	47,006 (14.1)	7,402 (12.7)	0.053
History of heart failure	68,610 (20.6)	10,811 (18.6)	0.059
Heart failure, this admission	49,109 (14.7)	7,603 (13.1)	0.058
Non-cardiac			
Respiratory insufficiency	141,751 (42.5)	20,531 (35.3)	0.148
Diabetes Mellitus	102,151 (30.6)	16,711 (28.7)	0.052
Renal insufficiency	109,584 (32.9)	16,526 (28.4)	0.099
Metastatic or hematologic malignancy	36,389 (10.9)	5,190 (8.9)	0.074
Hypotension or hypoperfusion	87,542 (26.3)	10,718 (18.4)	0.189
Pneumonia	44,553 (13.4)	6,344 (10.9)	0.081
Baseline depression in CNS function	31,308 (9.4)	3,973 (6.8)	0.099
Metabolic or electrolyte abnormality	66,220 (19.9)	8,546 (14.7)	0.138
Sepsis	44,191 (13.3)	6,202 (10.7)	0.085
Acute CNS non-stroke event	27,666 (8.3)	3,337 (5.7)	0.105
Hepatic insufficiency	25,975 (7.8)	3,210 (5.5)	0.097
Acute stroke	12,104 (3.6)	2,050 (3.5)	0.039
Major trauma	14,479 (4.3)	2,193 (3.8)	0.047
**Cardiac Arrest Characteristics**			
Year of cardiac arrest, n (%)			0.222
2000-2005	56,274 (16.9)	12,390 (21.3)	
2006-2010	79,692 (23.9)	11,866 (20.4)	
2011-2015	86,140 (25.8)	17,529 (30.2)	
2016-2021	111,245 (33.4)	16,348 (28.1)	

Interventions in place at time of cardiac arrest, n (%)			
Noninvasive assisted ventilation	1,550 (0.5)	186 (0.3)	0.023
Mechanical ventilation	71,860 (21.6)	10,025 (17.2)	0.113
Dialysis	11,020 (3.3)	1,387 (2.4)	0.065
Implantable cardiac defibrillator	5,809 (1.7)	840 (1.4)	0.042
Intra-arterial catheter	34,655 (10.4)	5,505 (9.5)	0.042
Electrocardiogram monitor	271,294 (81.4)	45,026 (77.5)	0.100
Pulse oximeter	235,372 (70.6)	38,220 (65.7)	0.107
Vasoactive agents	84,676 (25.4)	10,950 (18.8)	0.161
Antiarrhythmic agents	8,250 (2.5)	1,436 (2.5)	0.034
Location of cardiac arrest, n (%)			0.227
Emergency department	40,586 (12.2)	6,497 (11.2)	
Floor with telemetry or step down unit	52,396 (15.7)	9,334 (16.1)	
Floor without telemetry	52,958 (15.9)	9,360 (16.1)	
Intensive care unit or coronary care unit	157,294 (47.2)	24,629 (42.4)	
Operating room, post-anesthesia care unit, cardiac cath lab, or diagnostic/interventional unit	23,304 (7.0)	6,109 (10.5)	
Other[c]	6,674 (2.0)	2,011 (3.5)	
Unknown	139 (0.04)	193 (0.3)	
Witness status, n (%)			0.031
Witnessed	284,117 (85.2)	48,897 (84.1)	
Unwitnessed	49,234 (14.8)	9,236 (15.9)	
First documented rhythm, n (%)			0.475
Asystole	96,494 (28.9)	13,465 (23.2)	
Pulseless electrical activity	161,570 (48.5)	21,210 (36.5)	
Ventricular fibrillation	30,722 (9.2)	6,362 (10.9)	

Pulseless ventricular tachycardia	21,736 (6.5)	4,160 (7.2)	
Unknown	22,829 (6.8)	12,936 (22.3)	
Epinephrine administration, n (%)	298,793 (89.6)	45,170 (77.7)	0.327
Interval between initiation of chest compression and 1st epinephrine administration, median (IQR), minutes	1 (0-3)	1 (0-3)	0.002
Endotracheal intubation, n (%)	166,986 (50.1)	28,780 (49.5)	0.052
Interval between initiation of chest compression and endotracheal intubation, median (IQR), minutes	5 (2-9)	5 (2-10)	0.012

[a] Asian, Native Americans/Alaska Natives, "Others", and Native Hawaiians/Pacific Islanders.

[b] Obstetrics and "other"

[c] Ambulatory and outpatient areas; delivery suite; rehabilitation, skilled nursing, and mental health facilities; same-day surgical areas; and “other.”

Supplemental Table B. Resuscitation duration when point estimates of time-dependent probabilities of outcomes among those pending the first return of spontaneous circulation using the denominator including patients who had termination of resuscitation prior to or at each minute of cardiopulmonary resuscitation becomes less than 1%

	Outcome	
	Survival to hospital discharge	Favorable functional status at hospital discharge
**Patient population**		
All patients	39 minutes	32 minutes
Clinical features		
Age group, <60 years	44 minutes	36 minutes
Age group, 60-79 years	39 minutes	32 minutes
Age group, ≥80 years	29 minutes	22 minutes
Witnessed	40 minutes	33 minutes
Unwitnessed	31 minutes	24 minutes
Shockable	55 minutes	47 minutes
Nonshockable	35 minutes	27 minutes
Phenotypes		
Age group, <60 years; Witnessed; and Shockable	62 minutes	54 minutes
Age group, <60 years; Witnessed; and Nonshockable	40 minutes	32 minutes
Age group, <60 years; Unwitnessed; and Shockable	55 minutes	42 minutes
Age group, <60 years; Unwitnessed; and Nonshockable	33 minutes	25 minutes
Age group, 60-79 years; Witnessed; and Shockable	56 minutes	48 minutes
Age group, 60-79 years; Witnessed; and Nonshockable	36 minutes	30 minutes
Age group, 60-79 years; Unwitnessed; and Shockable	48 minutes	39 minutes
Age group, 60-79 years; Unwitnessed; and Nonshockable	27 minutes	19 minutes
Age group, ≥80 years; Witnessed; and Shockable	43 minutes	34 minutes
Age group, ≥80 years; Witnessed; and Nonshockable	27 minutes	21 minutes
Age group, ≥80 years; Unwitnessed; and Shockable	30 minutes	29 minutes
Age group, ≥80 years; Unwitnessed; and Nonshockable	21 minutes	16 minutes

Subgroup of patients between 2011 and 2021		
All patients	42 minutes	33 minutes
Clinical features		
Age group, <60 years	48 minutes	38 minutes
Age group, 60-79 years	41 minutes	33 minutes
Age group, ≥80 years	30 minutes	21 minutes
Witnessed	43 minutes	35 minutes
Unwitnessed	34 minutes	25 minutes
Shockable	59 minutes	49 minutes
Nonshockable	37 minutes	29 minutes
Phenotypes		
Age group, <60 years; Witnessed; and Shockable	66 minutes	54 minutes
Age group, <60 years; Witnessed; and Nonshockable	43 minutes	35 minutes
Age group, <60 years; Unwitnessed; and Shockable	57 minutes	50 minutes
Age group, <60 years; Unwitnessed; and Nonshockable	36 minutes	26 minutes
Age group, 60-79 years; Witnessed; and Shockable	59 minutes	50 minutes
Age group, 60-79 years; Witnessed; and Nonshockable	39 minutes	30 minutes
Age group, 60-79 years; Unwitnessed; and Shockable	64 minutes	41 minutes
Age group, 60-79 years; Unwitnessed; and Nonshockable	27 minutes	21 minutes
Age group, ≥80 years; Witnessed; and Shockable	48 minutes	35 minutes
Age group, ≥80 years; Witnessed; and Nonshockable	28 minutes	19 minutes
Age group, ≥80 years; Unwitnessed; and Shockable	41 minutes	41 minutes
Age group, ≥80 years; Unwitnessed; and Nonshockable	22 minutes	15 minutes

Supplemental Figure A

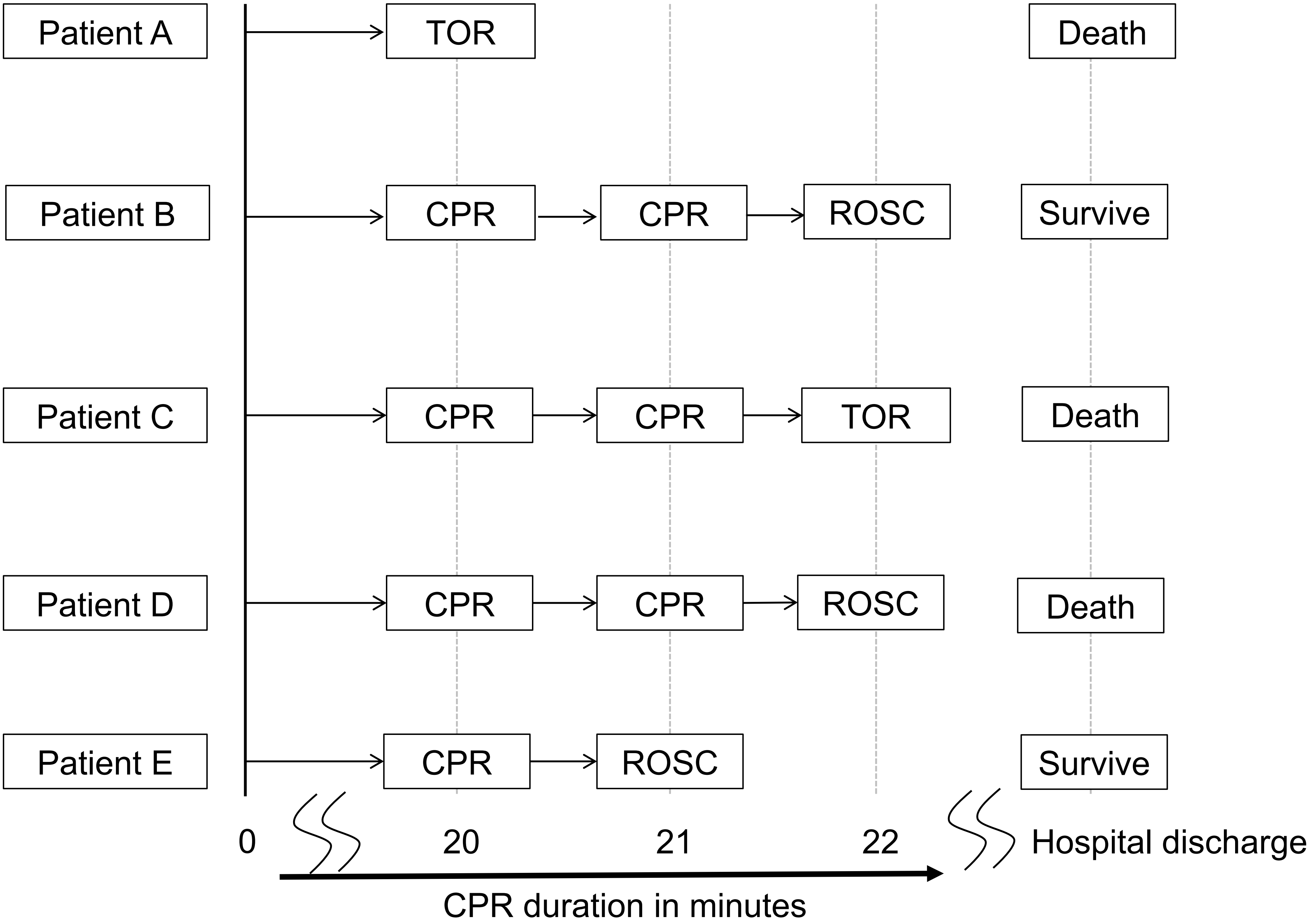

Supplemental Figure B1

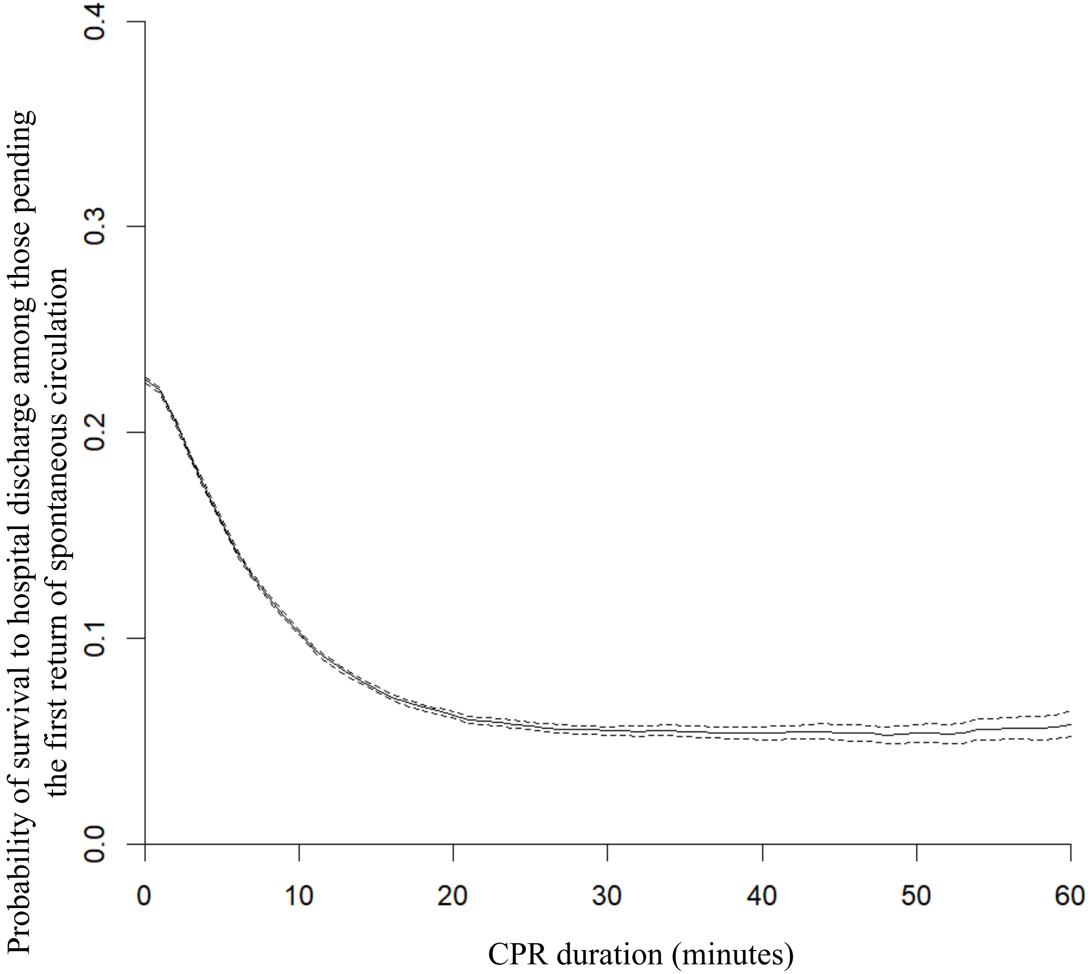

	CPR duration, minutes						
	1	10	20	30	40	50	60
Survival to hospital discharge, n / Receiving CPR, n (%)	75,645/343,581 (22.0%)	19,274/187,565 (10.3%)	5,769/91,949 (6.3%)	2,307/42,115 (5.5%)	1,066/19,889 (5.4%)	551/10,240 (5.4%)	320/5,505 (5.8%)

Supplemental Figure B2

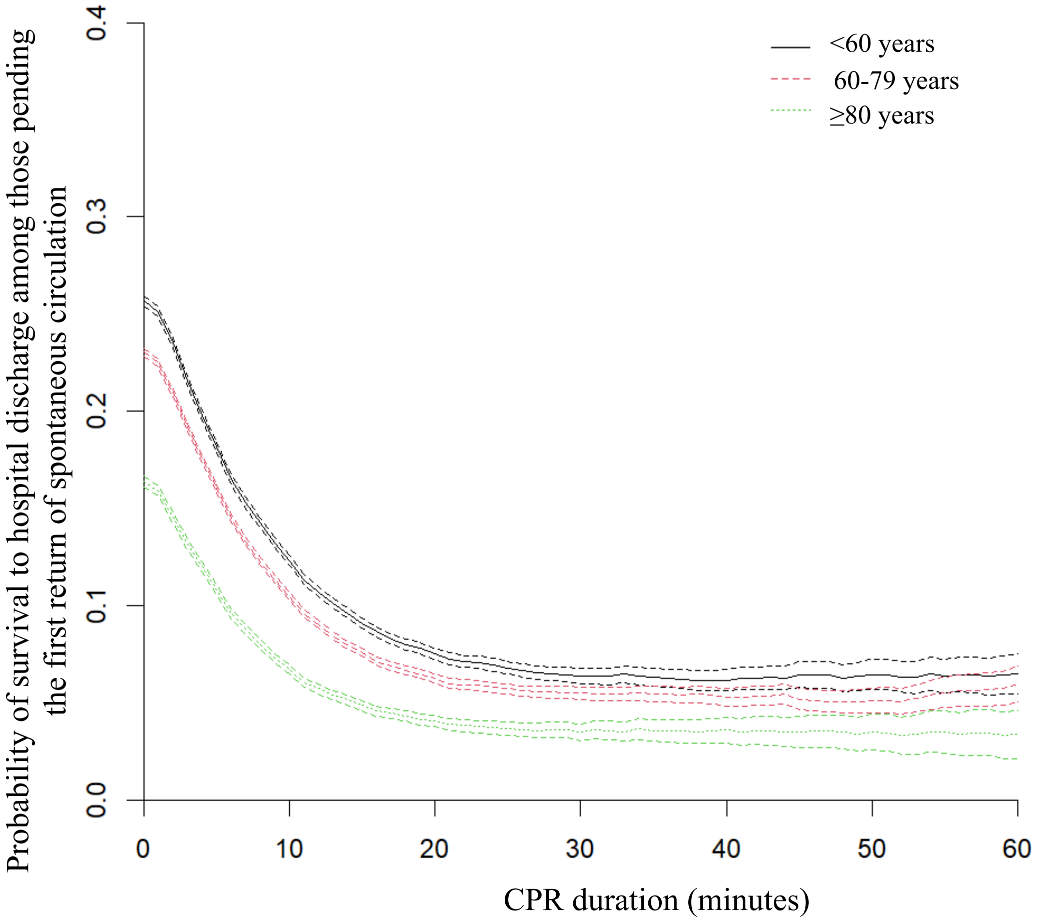

		CPR duration, minutes						
		1	10	20	30	40	50	60
Survival to hospital discharge, n / Receiving CPR, n (%)	<60 years	27,845/110,953 (25.1%)	7,314/59,408 (12.3%)	2,319/30,800 (7.5%)	967/15,179 (6.4%)	463/7,512 (6.2%)	260/4,035 (6.4%)	144/2,221 (6.5%)
	60-79 years	37,024/164,780 (22.5%)	9,351/89,260 (10.5%)	2,741/43,731 (6.3%)	1,098/19,994 (5.5%)	494/9,345 (5.3%)	237/4,666 (5.1%)	150/2,507 (6.0%)
	≥80 years	10,776/67,848 (15.9%)	2,609/38,897 (6.7%)	709/17,418 (4.1%)	242/6,942 (3.5%)	109/3,032 (3.6%)	54/1,539 (3.5%)	26/777 (3.3%)

Supplemental Figure B3

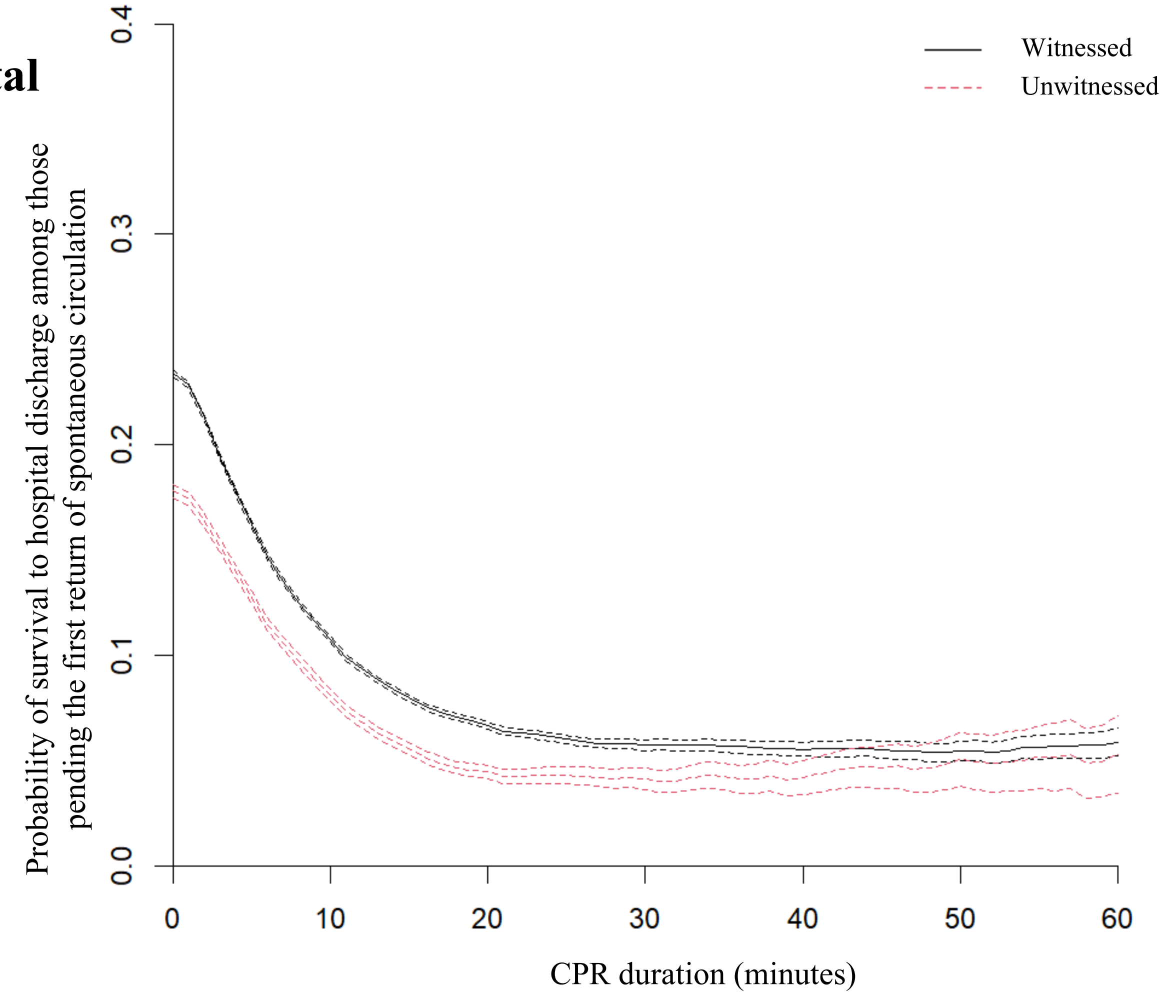

		CPR duration, minutes						
		1	10	20	30	40	50	60
Survival to hospital discharge, n / Receiving CPR, n (%)	Witnessed	66,812/292,894 (22.8%)	16,550/153,884 (10.8%)	5,056/75,971 (6.7%)	2,054/35,971 (5.7%)	961/17,374 (5.5%)	494/9,108 (5.4%)	290/4,935 (5.9%)
	Unwitnessed	8,833/50,687 (17.4%)	2,724/33,681 (8.1%)	713/15,978 (4.5%)	253/6,144 (4.1%)	105/2,515 (4.2%)	57/1,132 (5.0%)	30/570 (5.3%)

Supplemental Figure B4

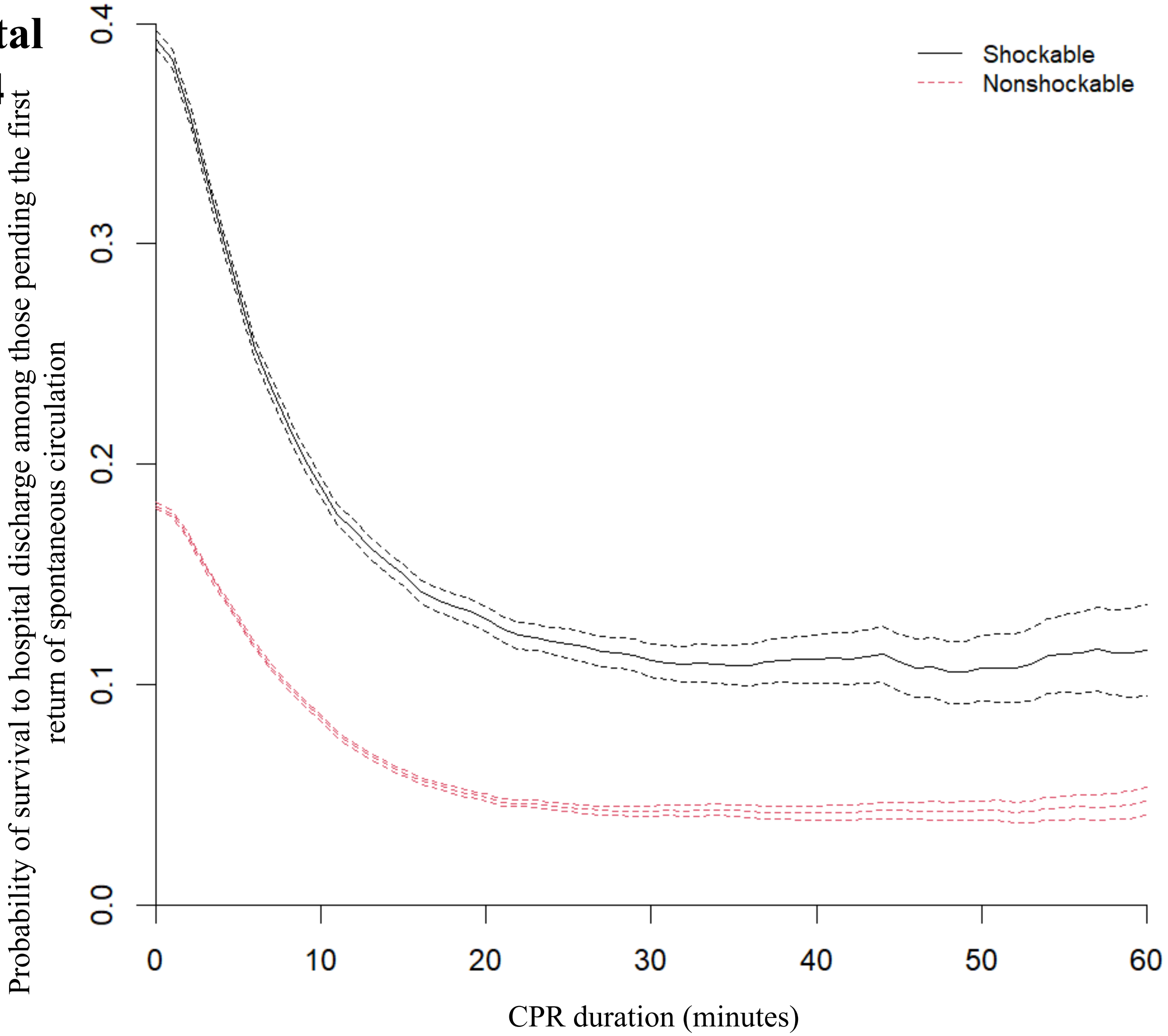

		CPR duration, minutes						
		1	10	20	30	40	50	60
Survival to hospital discharge, n / Receiving CPR, n (%)	Shockable	21,091/54,937 (38.4%)	5,031/26,544 (19.0%)	1,790/13,784 (13.0%)	742/6,704 (11.1%)	355/3,190 (11.1%)	179/1,665 (10.8%)	104/900 (11.6%)
	Nonshockable	46,948/264,596 (17.7%)	12,753/150,328 (8.5%)	3,542/72,852 (4.9%)	1,381/32,720 (4.2%)	636/15,294 (4.2%)	335/7,838 (4.3%)	197/4,201 (4.7%)

Supplemental Figure B5

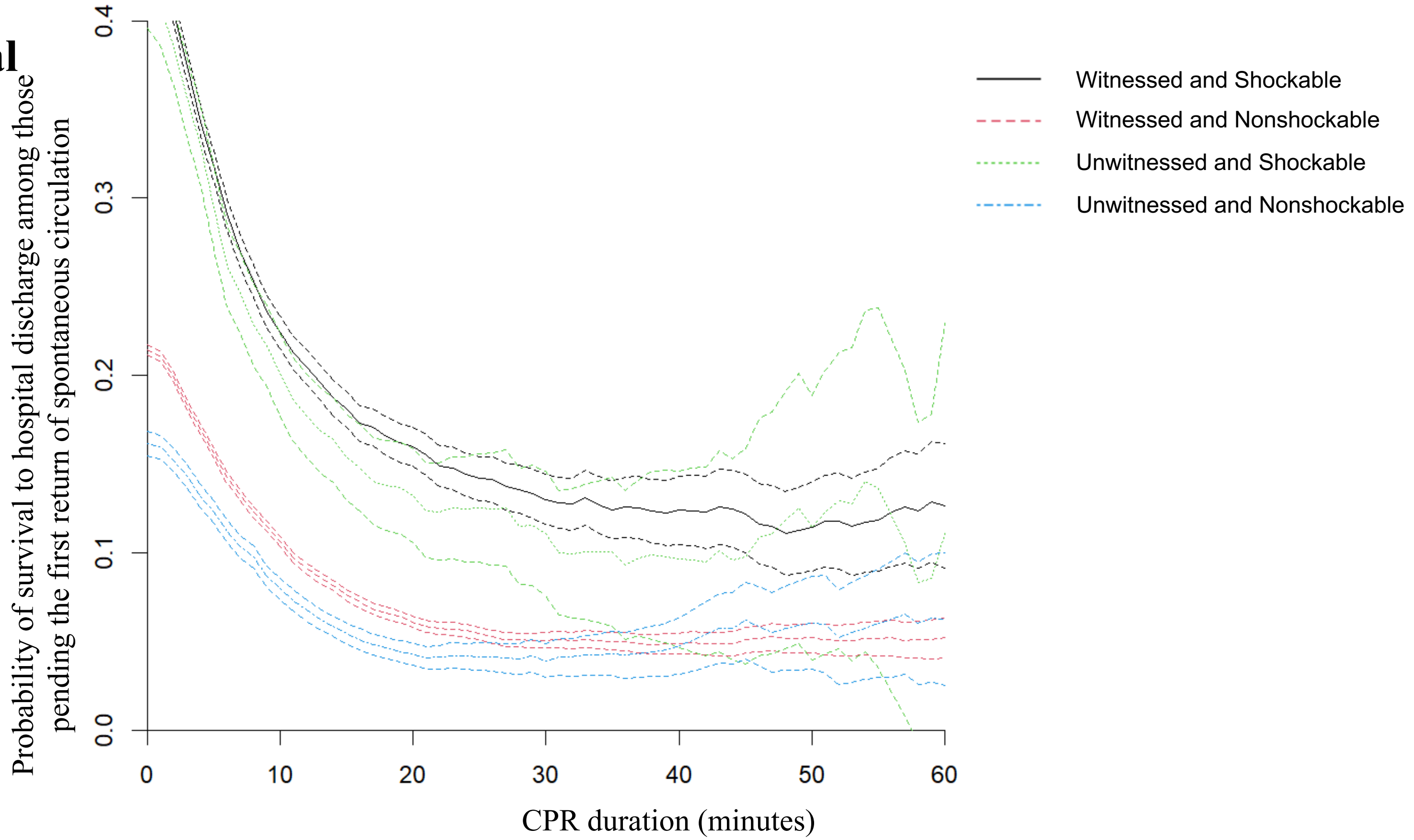

		CPR duration, minutes						
		1	10	20	30	40	50	60
Survival to hospital discharge, n / Receiving CPR, n (%)	Witnessed shockable	6,859/15,964 (43.0%)	1,698/7,573 (22.4%)	661/4,138 (16.0%)	287/2,208 (13.0%)	138/1,113 (12.4%)	71/620 (11.5%)	44/348 (12.6%)
	Witnessed nonshockaable	15,578/74,036 (21.0%)	4,207/39,591 (10.6%)	1,239/20,314 (6.1%)	511/10,113 (5.1%)	246/5,047 (4.9%)	143/2,751 (5.2%)	79/1,522 (5.2%)
	Unwitnessed shockable	823/2,022 (40.7%)	228/1,137 (20.1%)	84/636 (13.2%)	34/307 (11.1%)	13/135 (9.6%)	8/70 (11.4%)	3/27 (11.1%)
	Unwitnessed nonshockable	1,792/11,231 (16.0%)	622/7,810 (8.0%)	173/4,011 (4.3%)	63/1,615 (3.9%)	33/696 (4.7%)	19/315 (6.0%)	10/160 (6.3%)

Supplemental Figure B6

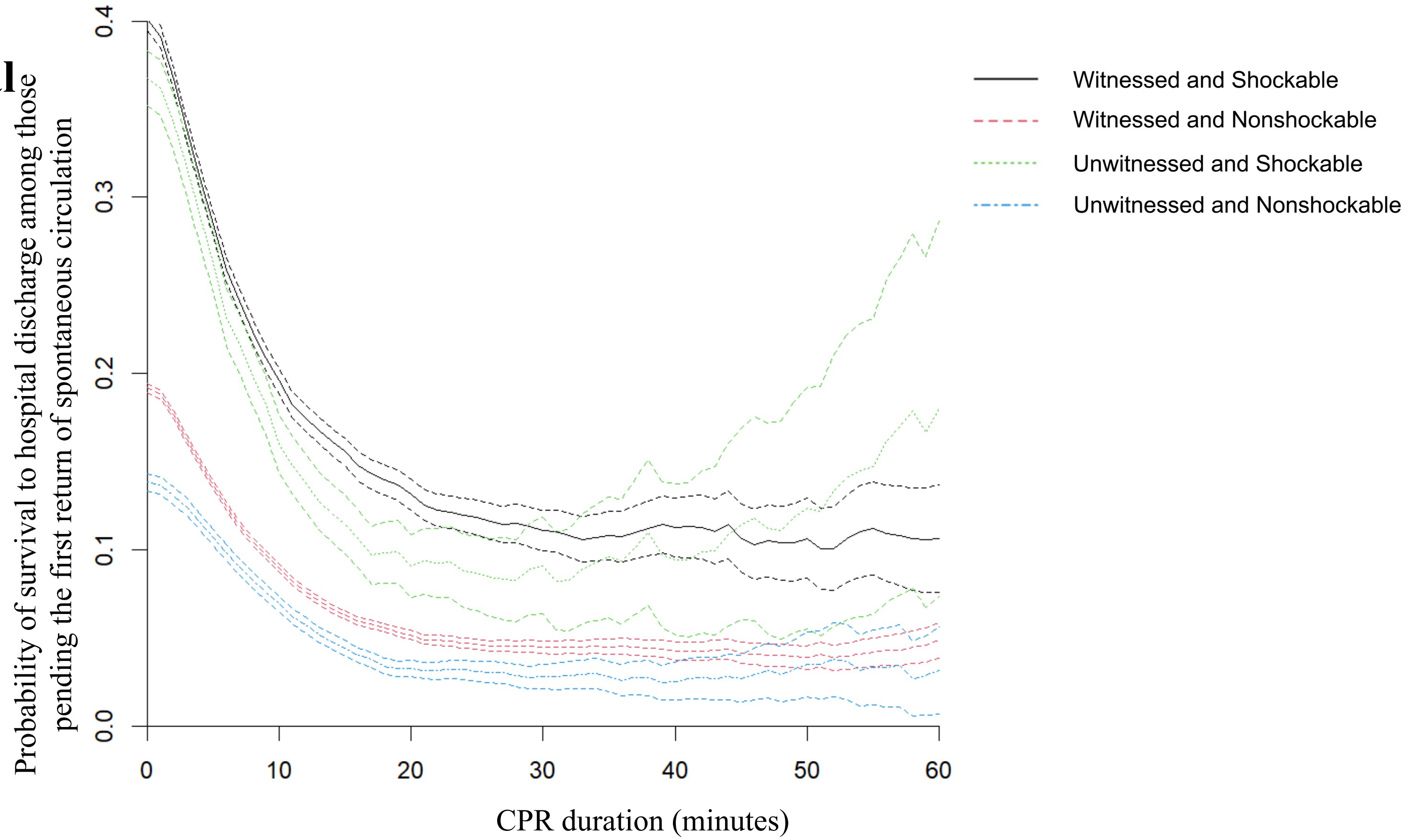

		CPR duration, minutes						
		1	10	20	30	40	50	60
Survival to hospital discharge, n / Receiving CPR, n (%)	Witnessed shockable	9,373/23,966 (39.1%)	2,175/11,108 (19.6%)	754/5,743 (13.1%)	316/2,857 (11.1%)	155/1,378 (11.2%)	74/695 (10.6%)	41/386 (10.6%)
	Witnessed nonshockaable	20,178/107,490 (18.8%)	5,254/58,534 (9.0%)	1,475/28,522 (5.2%)	589/13,225 (4.5%)	266/6,264 (4.2%)	123/3,176 (3.9%)	83/1,710 (4.9%)
	Unwitnessed shockable	1,303/3,599 (36.2%)	311/1,944 (16.0%)	92/1,013 (9.1%)	39/429 (9.1%)	17/180 (9.4%)	11/89 (12.4%)	9/50 (18.0%)
	Unwitnessed nonshockable	2,489/18,217 (13.7%)	869/12,560 (6.9%)	191/5,890 (3.2%)	62/2,216 (2.8%)	22/867 (2.5%)	13/373 (3.5%)	6/190 (3.2%)

Supplemental Figure B7

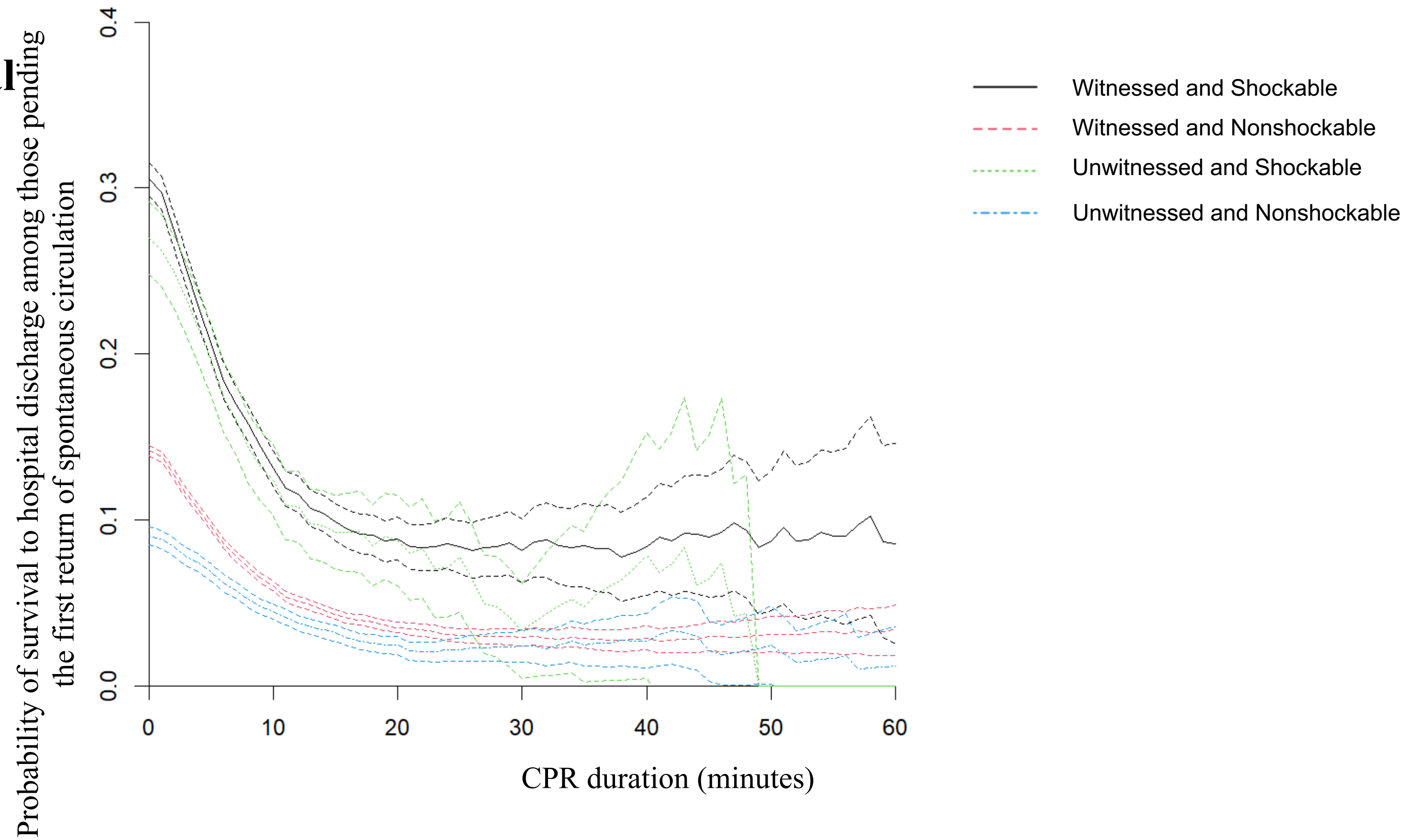

		CPR duration, minutes						
		1	10	20	30	40	50	60
Survival to hospital discharge, n / Receiving CPR, n (%)	Witnessed shockable	2,319/7,807 (29.7%)	503/3,847 (13.1%)	163/1,842 (8.8%)	61/751 (8.1%)	28/333 (8.4%)	15/172 (8.7%)	7/82 (8.5%)
	Witnessed nonshockaable	6,026/43,591 (13.8%)	1,482/24,679 (6.0%)	391/11,123 (3.5%)	133/4,585 (2.9%)	59/2,050 (2.9%)	33/1,060 (3.1%)	18/536 (3.4%)
	Unwitnessed shockable	414/1,579 (26.2%)	116/935 (12.4%)	36/412 (8.7%)	5/152 (3.3%)	4/51 (7.8%)	0/19 (0%)	0/7 (0%)
	Unwitnessed nonshockable	885/10,031 (8.8%)	319/7,154 (4.5%)	73/2,992 (2.4%)	23/966 (2.4%)	10/370 (2.7%)	4/163 (2.5%)	1/83 (1.2%)

Supplemental Figure C1

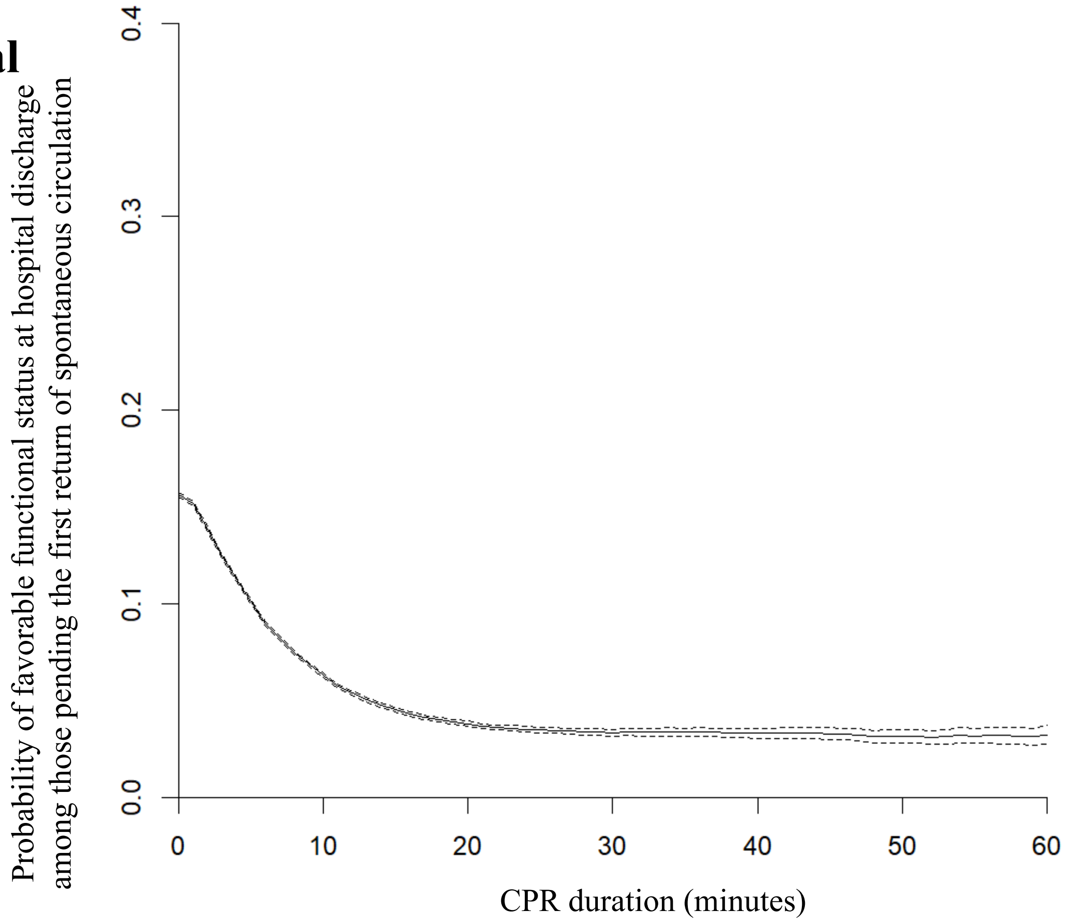

	CPR duration, minutes						
	1	10	20	30	40	50	60
Favorable functional status at hospital discharge, n / Receiving CPR, n (%)	49,769/328,486 (15.2%)	11,576/183,311 (6.3%)	3,444/90,629 (3.8%)	1,379/41,583 (3.3%)	646/19,645 (3.3%)	320/10,105 (3.2%)	175/5,419 (3.2%)

Supplemental Figure C2

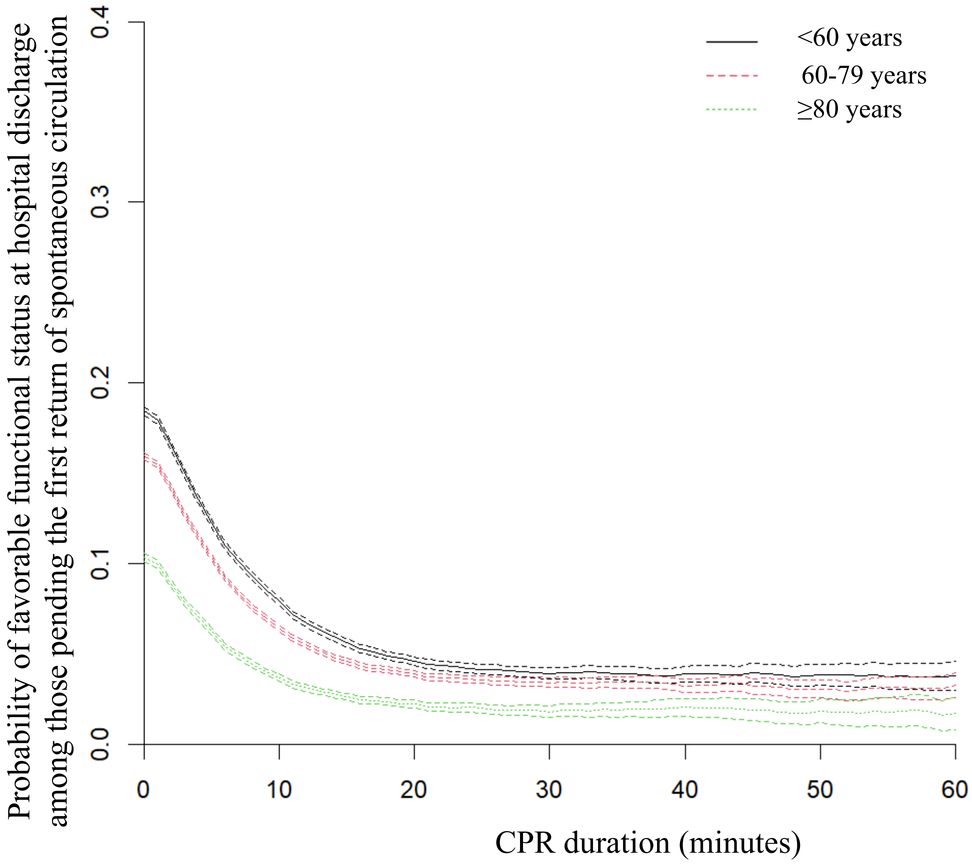

		CPR duration, minutes						
		1	10	20	30	40	50	60
Favorable functional status at hospital discharge, n / Receiving CPR, n (%)	<60 years	18,936/105,523 (17.9%)	4,574/57,854 (7.9%)	1,390/30,262 (4.6%)	585/14,952 (3.9%)	287/7,404 (3.9%)	152/3,969 (3.8%)	82/2,183 (3.8%)
	60-79 years	24,319/157,389 (15.5%)	5,593/87,167 (6.4%)	1,671/43,115 (3.9%)	672/19,751 (3.4%)	298/9,232 (3.2%)	140/4,609 (3.0%)	80/2,465 (3.2%)
	≥80 years	6,514/65,574 (9.9%)	1,409/38,290 (3.7%)	383/17,252 (2.2%)	122/6,880 (1.8%)	61/3,009 (2.0%)	28/1,527 (1.8%)	13/771 (1.7%)

Supplemental Figure C3

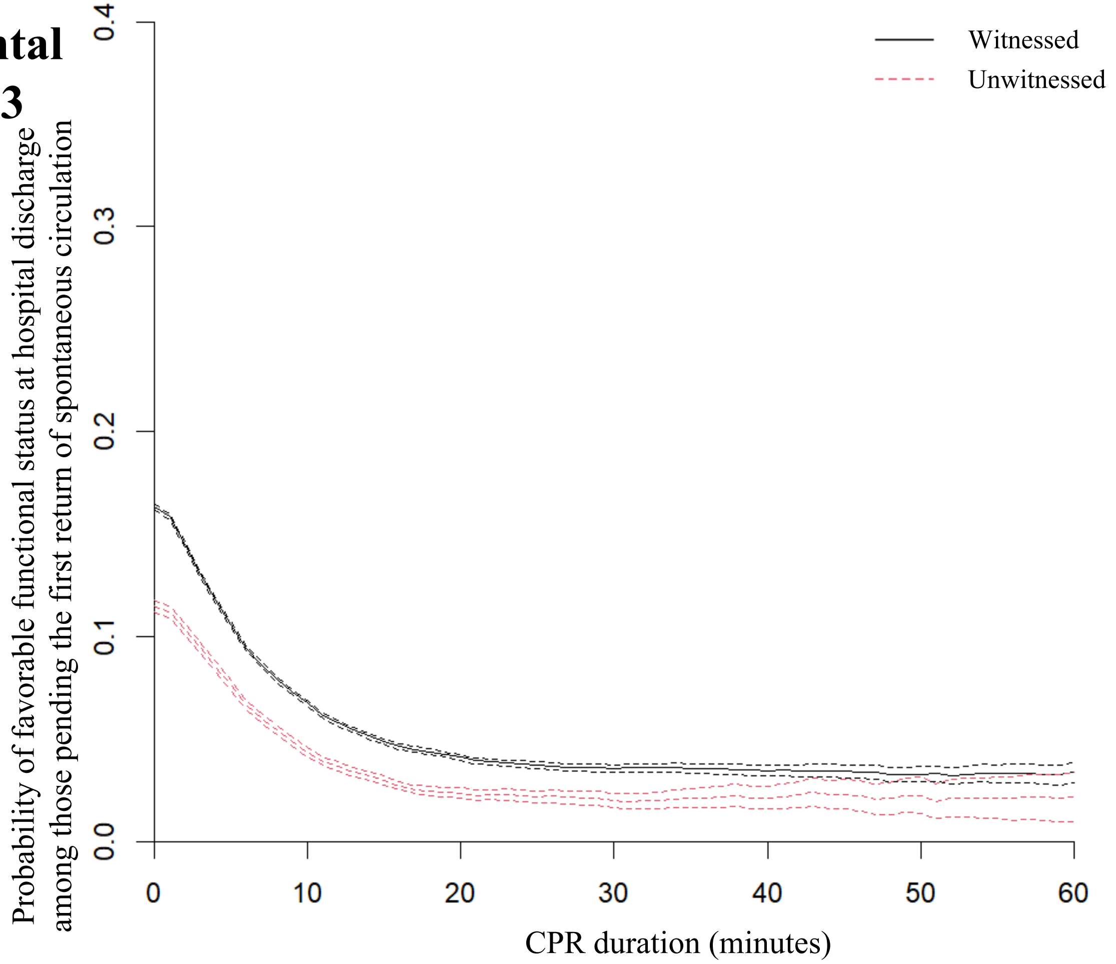

		CPR duration, minutes						
		1	10	20	30	40	50	60
Favorable functional status at hospital discharge, n / Receiving CPR, n (%)	Witnessed	44,294/279,681 (15.8%)	10,133/150,297 (6.7%)	3,071/74,836 (4.1%)	1,257/35,508 (3.5%)	593/17,157 (3.5%)	295/8,992 (3.3%)	163/4,861 (3.4%)
	Unwitnessed	5,475/48,805 (11.2%)	1,443/33,014 (4.4%)	373/15,793 (2.4%)	122/6,075 (2.0%)	53/2,488 (2.1%)	25/1,113 (2.2%)	12/558 (2.2%)

Supplemental Figure C4

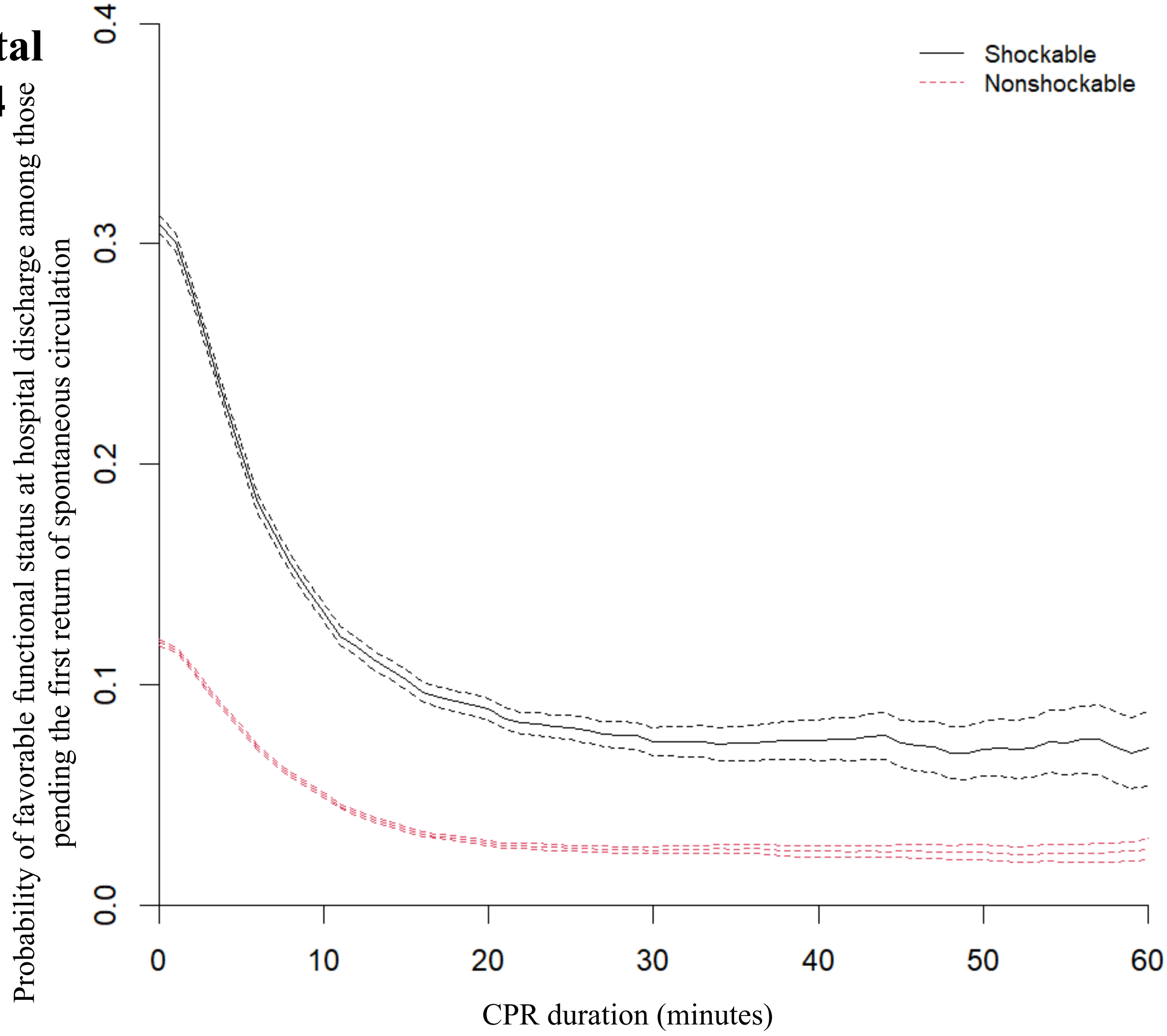

		CPR duration, minutes						
		1	10	20	30	40	50	60
Favorable functional status at hospital discharge, n / Receiving CPR, n (%)	Shockable	15,371/51,132 (30.1%)	3,386/25,493 (13.3%)	1,191/13,400 (8.9%)	482/6,536 (7.4%)	232/3,113 (7.5%)	115/1,624 (7.1%)	62/875 (7.1%)
	Nonshockable	29,641/255,201 (11.6%)	7,367/14,577 (5.0%)	2,015/72,052 (2.8%)	804/32,420 (2.5%)	368/15,147 (2.4%)	185/7,754 (2.4%)	105/4,146 (2.5%)

Supplemental Figure C5

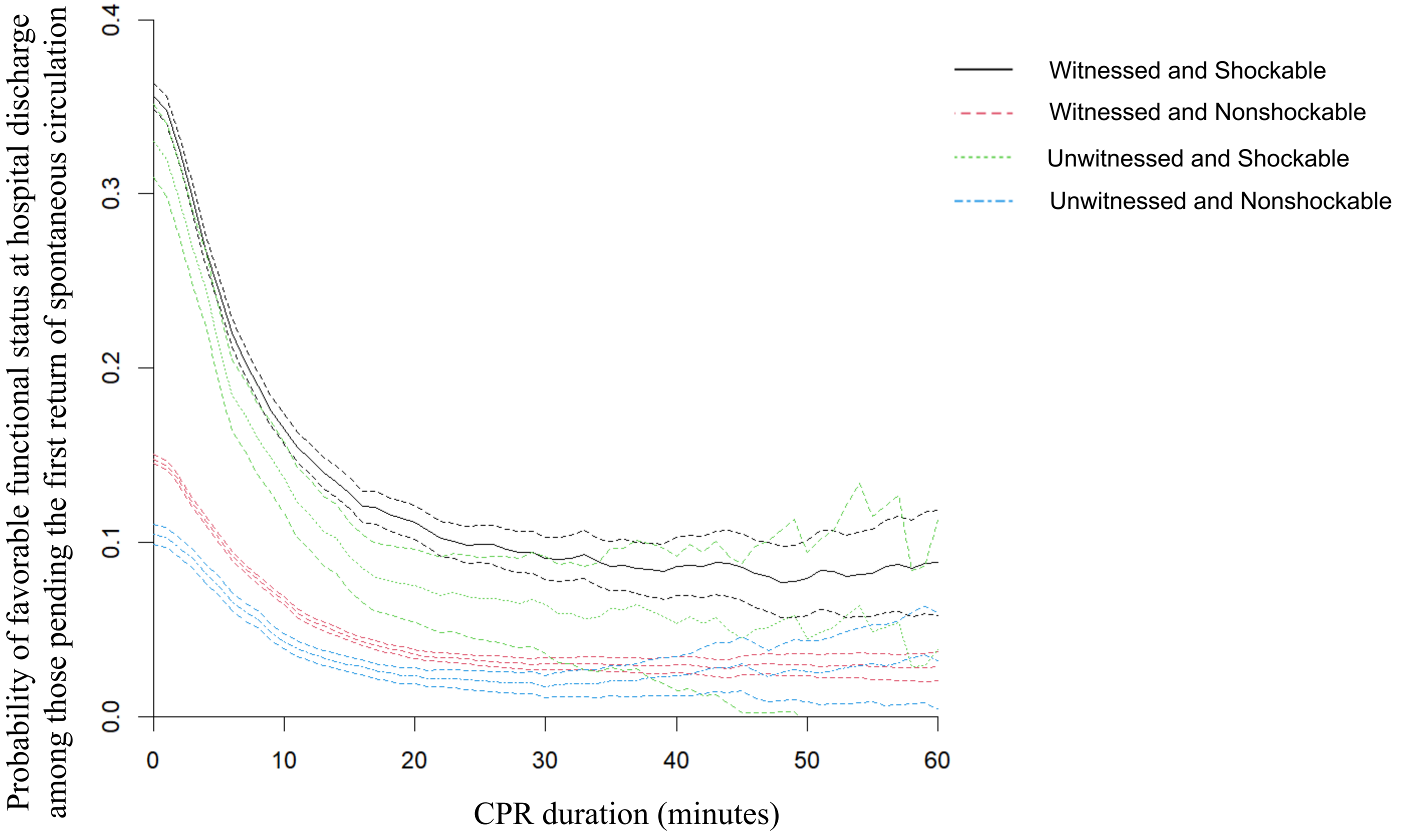

		CPR duration, minutes						
		1	10	20	30	40	50	60
Favorable functional status at hospital discharge, n / Receiving CPR, n (%)	Witnessed shockable	5,139/14,761 (34.8%)	1,195/7,246 (16.5%)	446/3,999 (11.2%)	195/2,146 (9.1%)	93/1,082 (8.6%)	48/604 (7.9%)	30/340 (8.8%)
	Witnessed nonshockaable	10,248/71,002 (14.4%)	2,577/38,723 (6.7%)	716/20,032 (3.6%)	303/9,998 (3.0%)	149/4,990 (3.0%)	81/2,714 (3.0%)	43/1,499 (2.9%)
	Unwitnessed shockable	599/1,874 (32.0%)	149/1,088 (13.7%)	46/612 (7.5%)	19/297 (6.4%)	7/131 (5.3%)	3/67 (4.5%)	1/26 (3.8%)
	Unwitnessed nonshockable	1,119/10,861 (10.3%)	331/7,665 (4.3%)	92/3,967 (2.3%)	27/1,598 (1.7%)	16/688 (2.3%)	8/309 (2.6%)	5/156 (3.2%)

Supplemental Figure C6

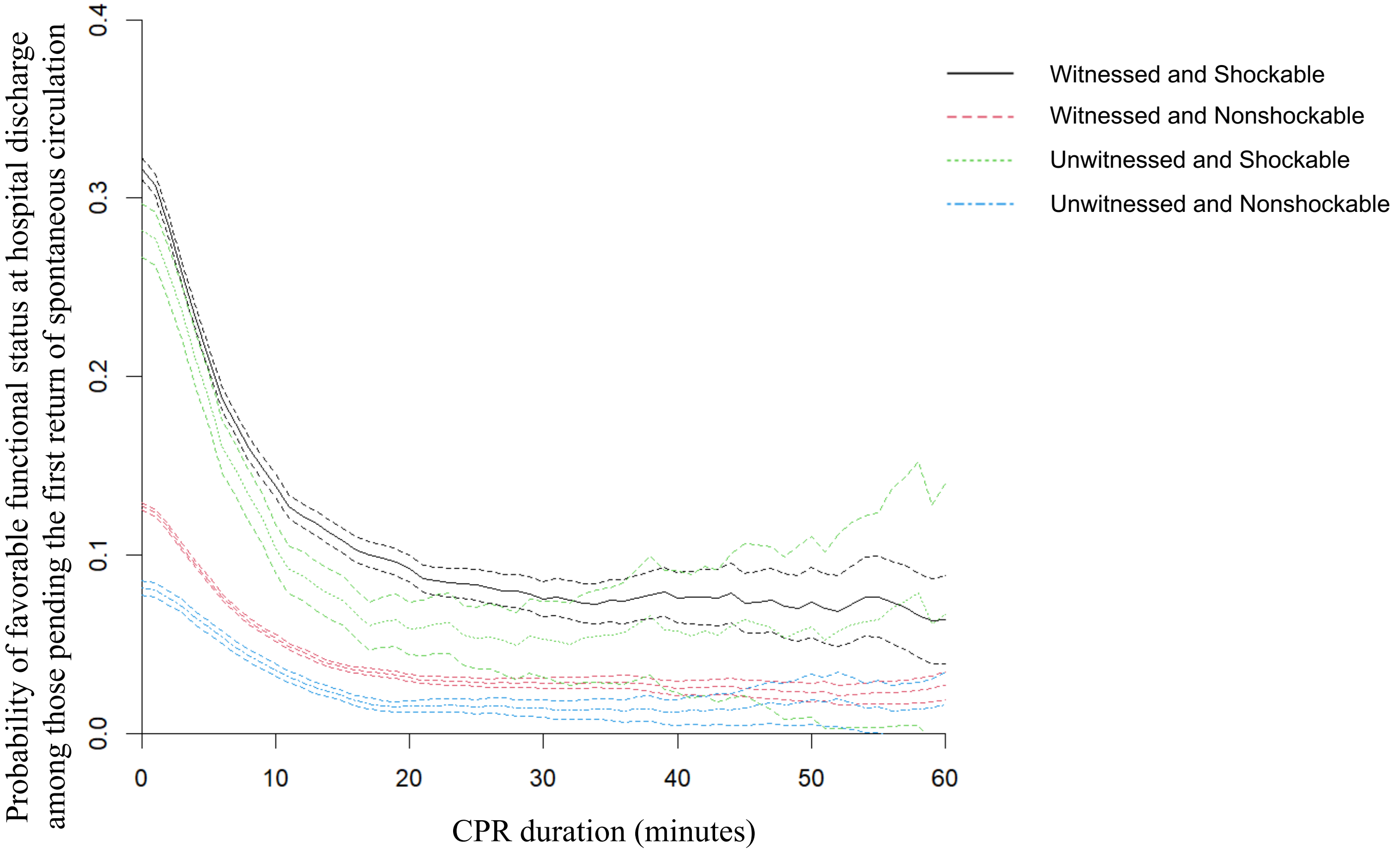

		CPR duration, minutes						
		1	10	20	30	40	50	60
Favorable functional status at hospital discharge, n / Receiving CPR, n (%)	Witnessed shockable	6,840/22,265 (30.7%)	1,476/10,644 (13.9%)	517/5,583 (9.3%)	210/2,788 (7.5%)	102/1,345 (7.6%)	50/681 (7.3%)	24/377 (6.4%)
	Witnessed nonshockaable	12,828/103,501 (12.4%)	3,076/57,415 (5.4%)	885/28,211 (3.1%)	366/13,109 (2.8%)	156/6,205 (2.5%)	71/3,148 (2.3%)	45/1,688 (2.7%)
	Unwitnessed shockable	931/3,360 (27.7%)	194/1,873 (10.4%)	58/989 (5.9%)	22/417 (5.3%)	10/175 (5.7%)	5/84 (6.0%)	3/45 (6.7%)
	Unwitnessed nonshockable	1,421/17,660 (8.0%)	439/12,351 (3.6%)	88/5,844 (1.5%)	31/2,203 (1.4%)	10/860 (1.2%)	7/368 (1.9%)	3/188 (1.6%)

Supplemental Figure C7

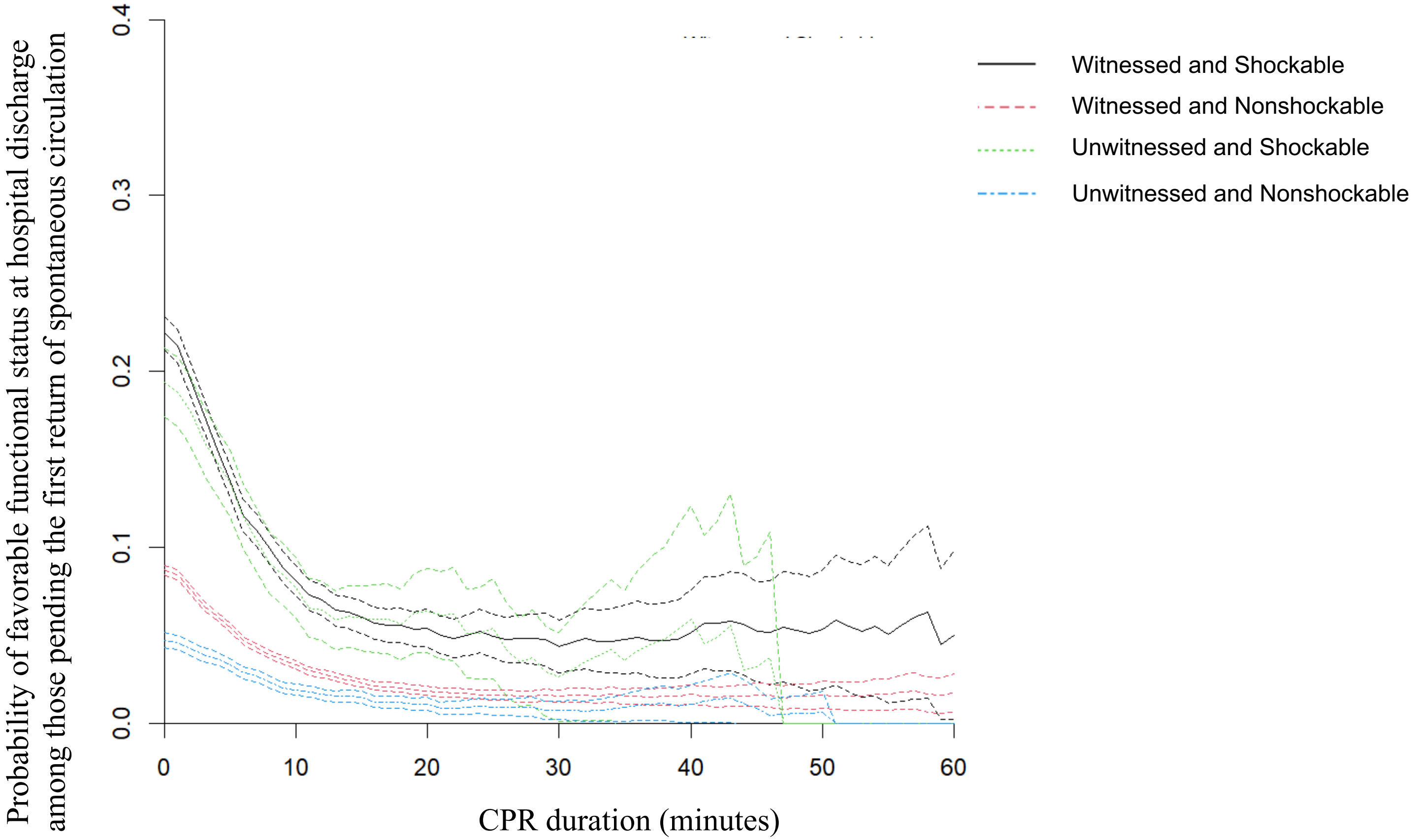

		CPR duration, minutes						
		1	10	20	30	40	50	60
Favorable functional status at hospital discharge, n / Receiving CPR, n (%)	Witnessed shockable	1,579/7,368 (21.4%)	302/3,731 (8.1%)	98/1,810 (5.4%)	32/736 (4.3%)	17/329 (5.2%)	9/169 (5.3%)	4/80 (5.0%)
	Witnessed nonshockaable	3,574/42,331 (8.4%)	810/24,345 (3.3%)	202/11,024 (1.8%)	70/4,552 (1.5%)	33/2,035 (1.6%)	17/1,052 (1.6%)	9/532 (1.7%)
	Unwitnessed shockable	283/1,504 (18.8%)	70/911 (7.7%)	26/407 (6.4%)	4/152 (2.6%)	3/51 (5.9%)	0/19 (0%)	0/7 (0%)
	Unwitnessed nonshockable	451/9,846 (4.6%)	134/7,078 (1.9%)	32/2,974 (1.1%)	7/960 (0.7%)	4/369 (1.1%)	1/163 (0.6%)	0/83 (0%)

Supplemental Figure D1

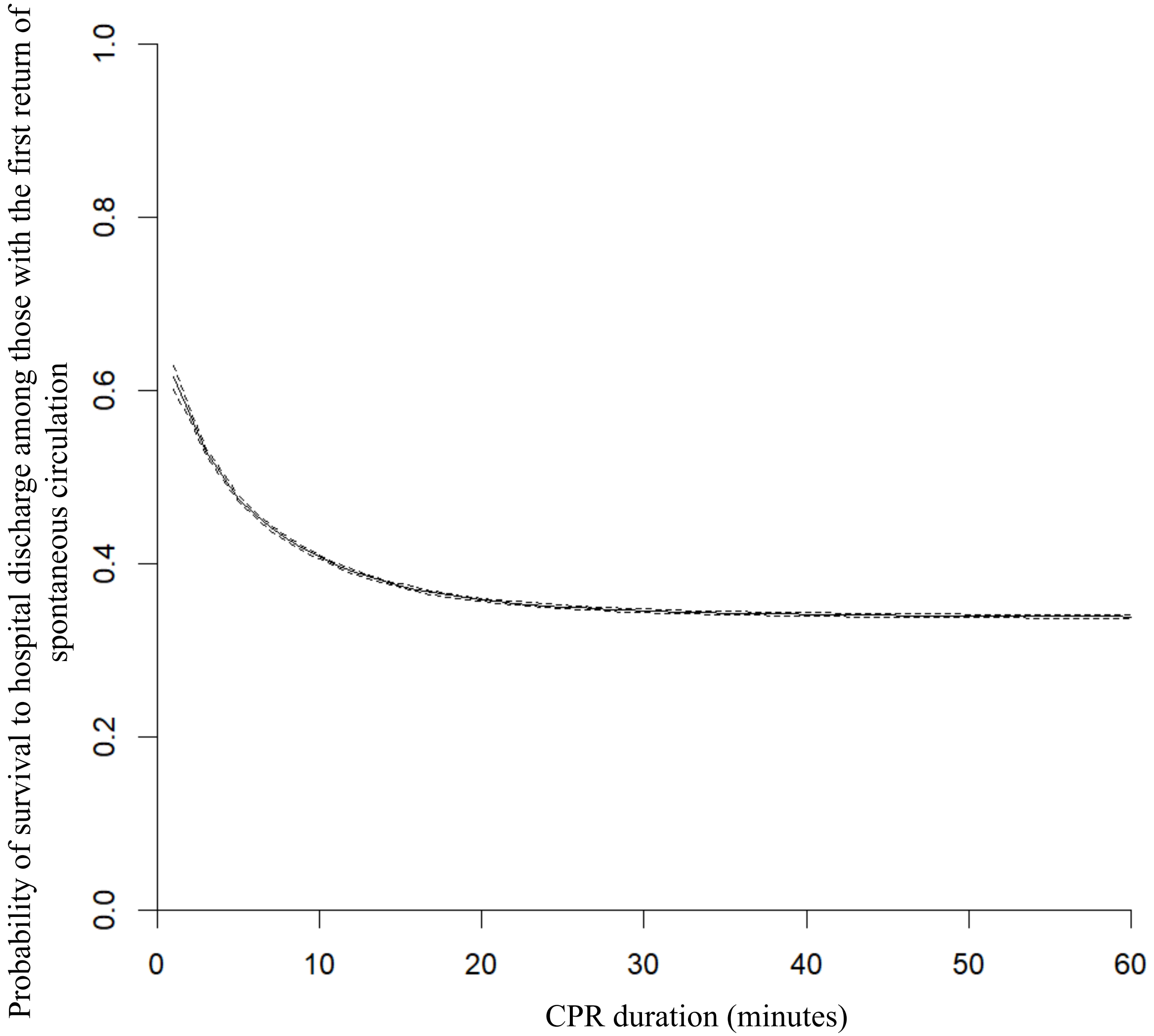

	CPR duration, minutes						
	1	10	20	30	40	50	60
Survival to hospital discharge, n / Achieving ROSC, n (%)	3,154/5,130 (61.5%)	59,525/146,101 (40.7%)	73,030/204,135 (35.8%)	76,492/221,553 (34.5%)	77,733/227,954 (34.1%)	78,248/230,705 (33.9%)	78,479/232,016 (33.8%)

Supplemental Figure D2

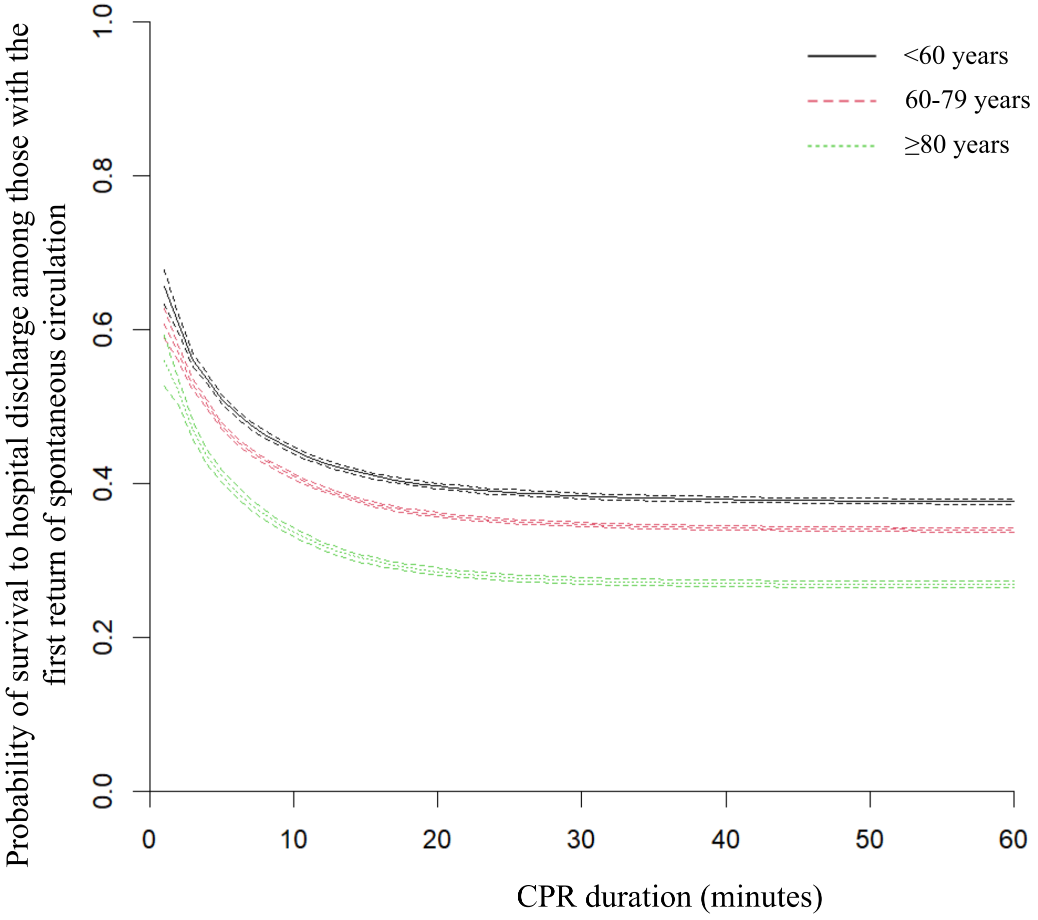

		CPR duration, minutes						
		1	10	20	30	40	50	60
Survival to hospital discharge, n / Achieving ROSC, n (%)	<60 years	1,103/1,684 (65.5%)	21,634/48,869 (44.3%)	26,629/67,221 (39.6%)	27,981/72,967 (38.3%)	28,485/75,208 (37.9%)	28,688/76,157 (37.7%)	28,804/76,658 (37.6%)
	60-79 years	1,566/2,579 (60.7%)	29,239/71,513 (40.9%)	35,849/99,894 (35.9%)	37,492/108,259 (34.6%)	38,096/111,412 (34.2%)	38,353/112,800 (34.0%)	38,440/113,421 (33.9%)
	≥80 years	485/867 (55.9%)	8,652/25,719 (33.6%)	10,552/37,020 (28.5%)	11,019/40,327 (27.3%)	11,152/41,334 (27.0%)	11,207/41,748 (26.8%)	11,235/41,937 (26.8%)

Supplemental Figure D3

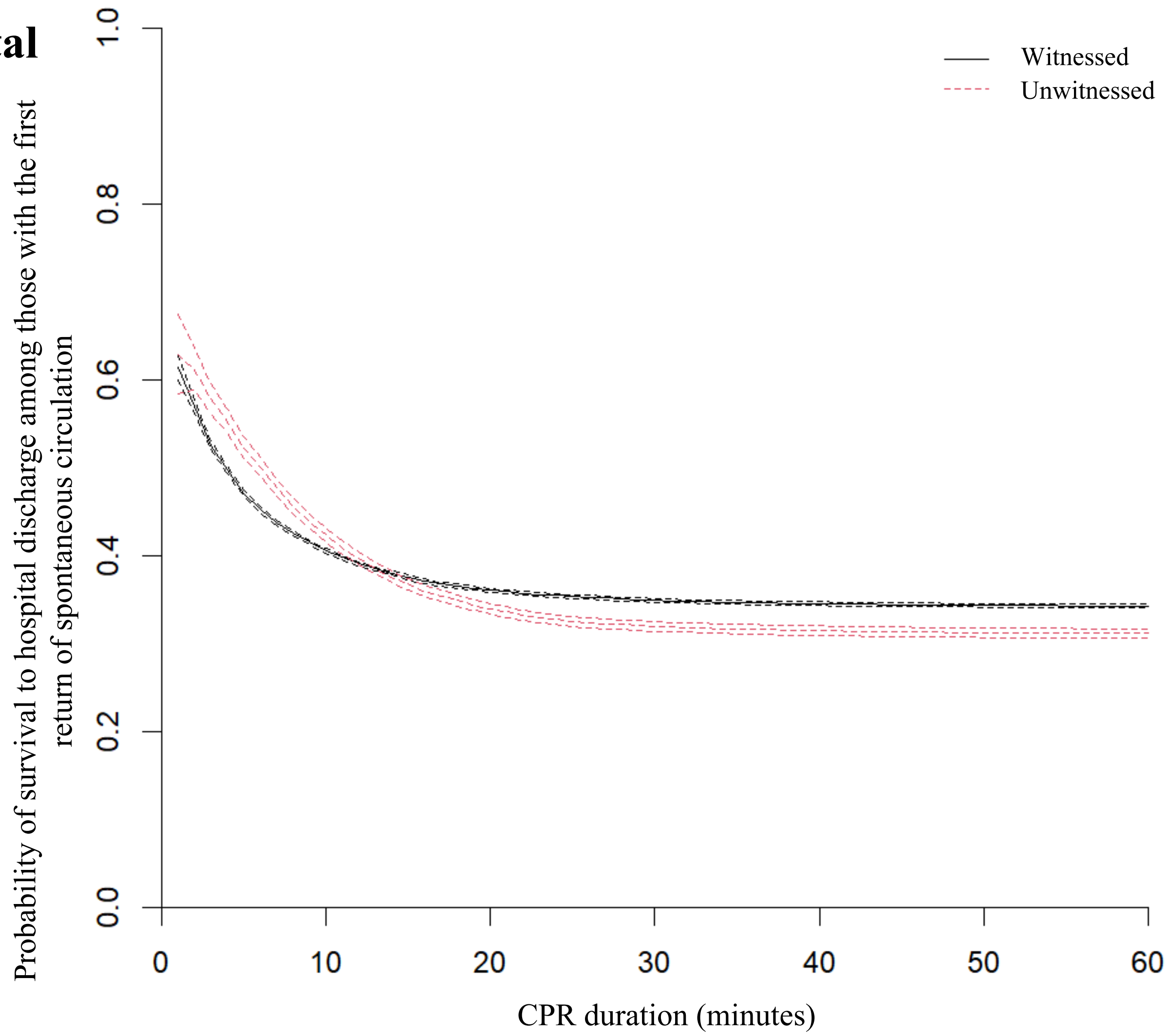

		CPR duration, minutes						
		1	10	20	30	40	50	60
Survival to hospital discharge, n / Achieving ROSC , n (%)	Witnessed	2,880/4,694 (61.4%)	53,142/131,035 (40.6%)	64,636/179,394 (36.0%)	67,638/193,802 (34.9%)	68,731/199,314 (34.5%)	69,198/201,691 (34.3%)	69,402/202,837 (34.2%)
	Unwitnessed	274/436 (62.8%)	6,383/15,066 (42.4%)	8,394/24,741 (33.9%)	8,854/27,751 (31.9%)	9,002/28,640 (31.4%)	9,050/29,014 (31.2%)	9,077/29,179 (31.1%)

Supplemental Figure D4

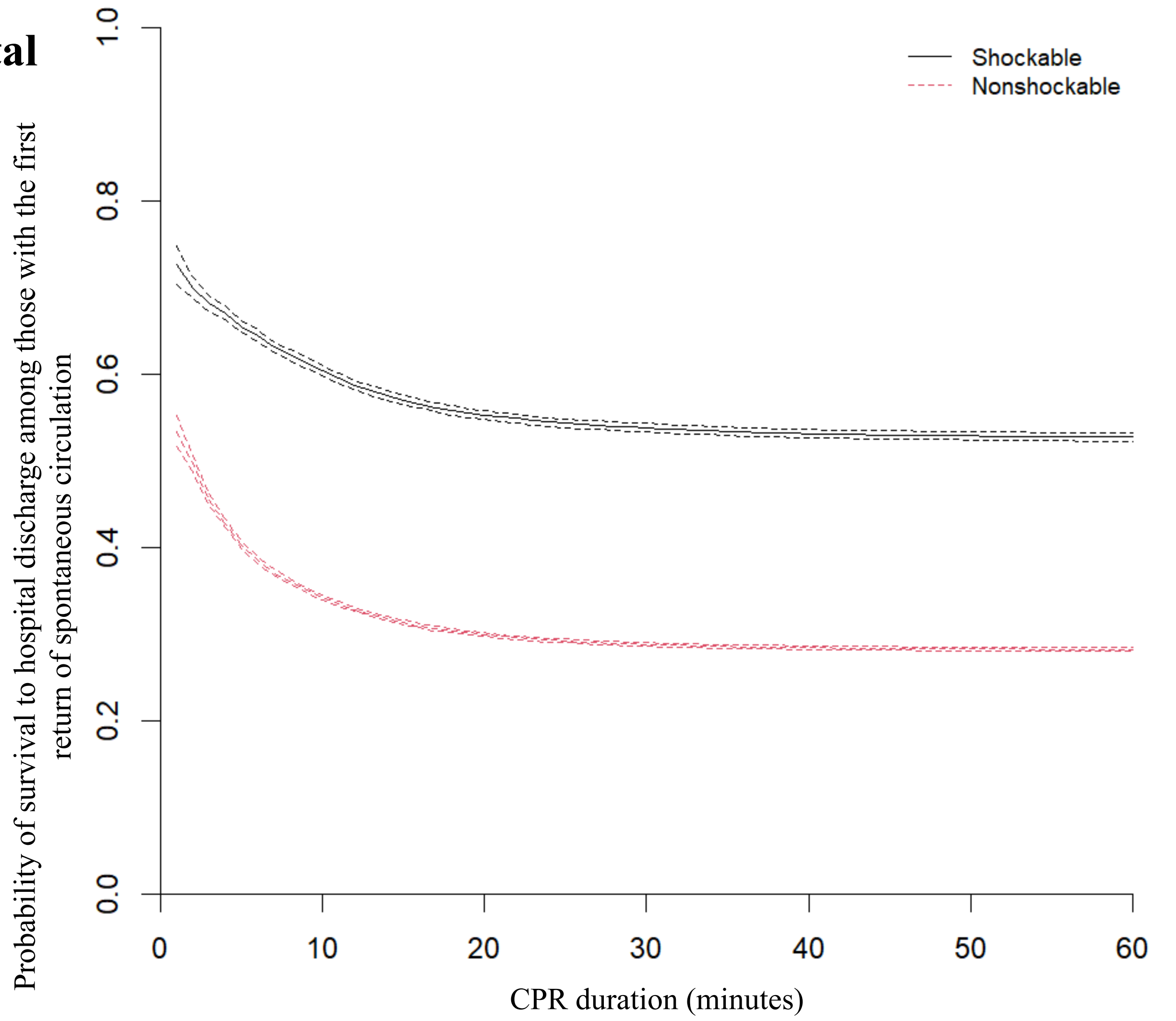

		CPR duration, minutes						
		1	10	20	30	40	50	60
Survival to hospital discharge, n / Achieving ROSC, n (%)	Shockable	1076/1483 (72.6%)	17136/28369 (60.4%)	20377/36908 (55.2%)	21425/39830 (53.8%)	21812/41085 (53.1%)	21988/41621 (52.8%)	22063/41876 (52.7%)
	Nonshockable	1536/2877 (53.4%)	35731/104460 (34.2%)	44942/150324 (29.9%)	47103/163727 (28.8%)	47848/168453 (28.4%)	48149/170482 (28.2%)	48287/171440 (28.2%)

Supplemental Figure D5

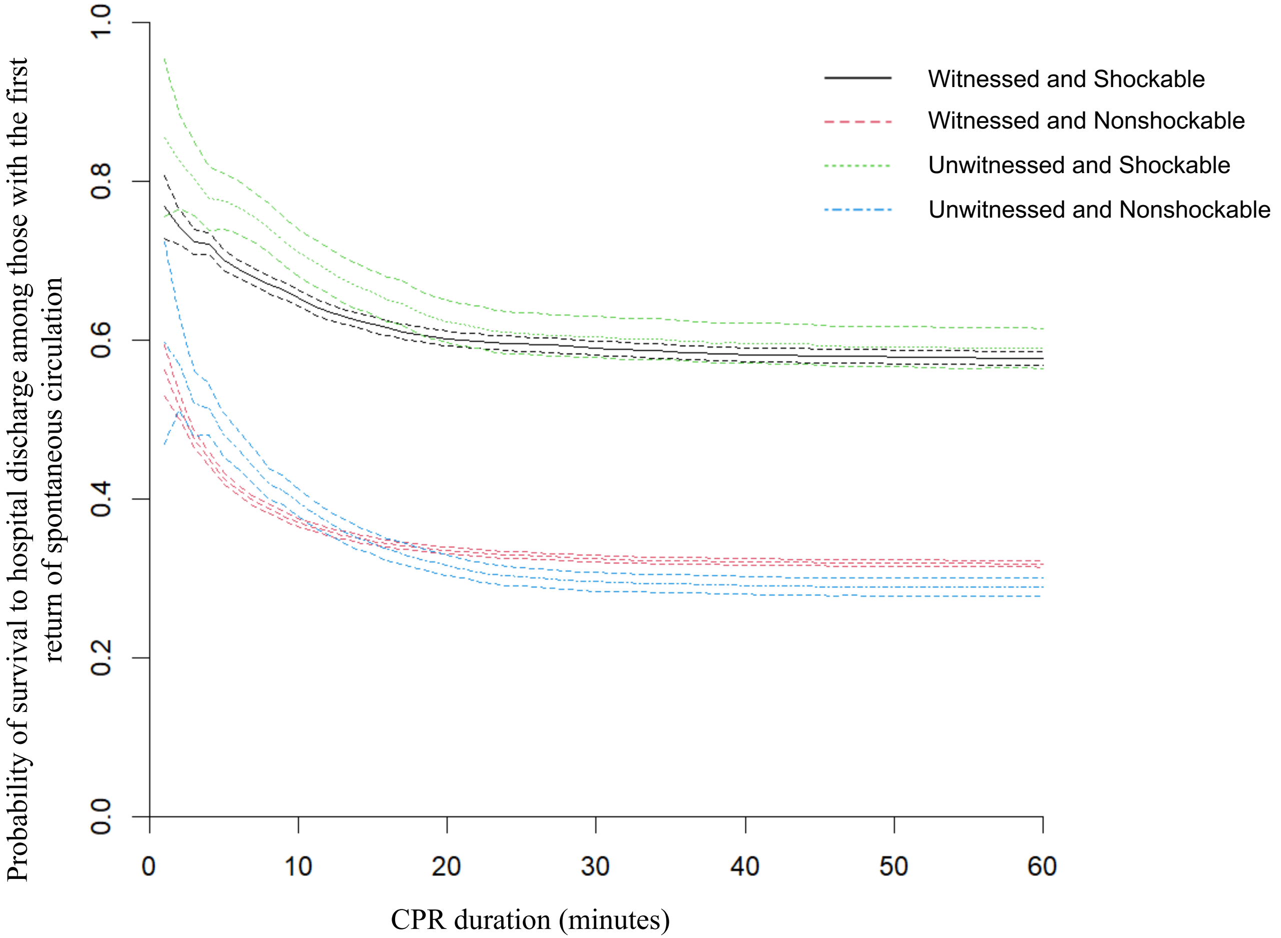

		CPR duration, minutes						
		1	10	20	30	40	50	60
Survival to hospital discharge, n / Achieving ROSC, n (%)	Witnessed shockable	326/425 (76.7%)	5487/8411 (65.2%)	6524/10848 (60.1%)	6898/11696 (59.0%)	7047/12128 (58.1%)	7114/12302 (57.8%)	7141/12390 (57.6%)
	Witnessed nonshockaable	507/903 (56.1%)	11878/32083 (37.0%)	14846/44324 (33.5%)	15574/48010 (32.4%)	15839/49425 (32.0%)	15942/50034 (31.9%)	16006/50357 (31.8%)
	Unwitnessed shockable	41/48 (85.4%)	636/896 (71.0%)	780/1252 (62.3%)	830/1375 (60.4%)	851/1429 (59.6%)	856/1448 (59.1%)	861/1462 (58.9%)
	Unwitnessed nonshockable	34/57 (59.6%)	1204/3044 (39.6%)	1653/5231 (31.6%)	1763/5973 (29.5%)	1793/6168 (29.1%)	1807/6257 (28.9%)	1816/6296 (28.8%)

Supplemental Figure D6

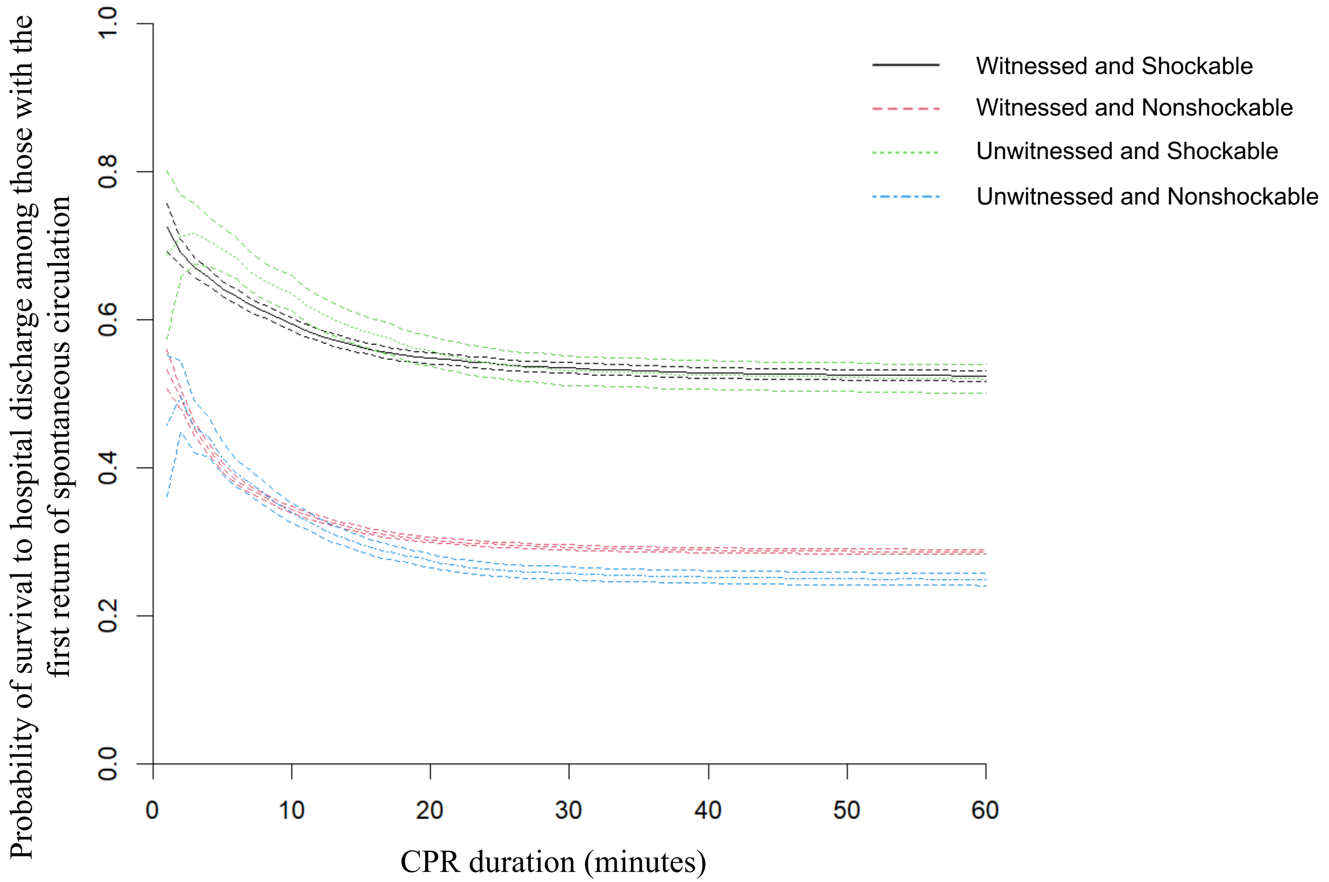

		CPR duration, minutes						
		1	10	20	30	40	50	60
Survival to hospital discharge, n / Achieving ROSC, n (%)	Witnessed shockable	520/718 (72.4%)	7718/13012 (59.3%)	9139/16701 (54.7%)	9577/17926 (53.4%)	9738/18458 (52.8%)	9819/18705 (52.5%)	9852/18814 (52.4%)
	Witnessed nonshockaable	699/1313 (53.2%)	15623/45544 (34.3%)	19402/64214 (30.2%)	20288/69521 (29.2%)	20611/71538 (28.8)	20754/72417 (28.7%)	20794/72814 (28.6%)
	Unwitnessed shockable	44/64 (68.8%)	1036/1629 (63.6%)	1255/2250 (55.8%)	1308/2465 (53.1%)	1330/2535 (52.5%)	1336/2560 (52.2%)	1338/2576 (51.9%)
	Unwitnessed nonshockable	47/103 (45.6%)	1667/4924 (33.9%)	2345/8569 (27.4%)	2474/9639 (25.7%)	2514/9979 (25.2%)	2523/10116 (24.9%)	2530/10171 (24.9%)

Supplemental Figure D7

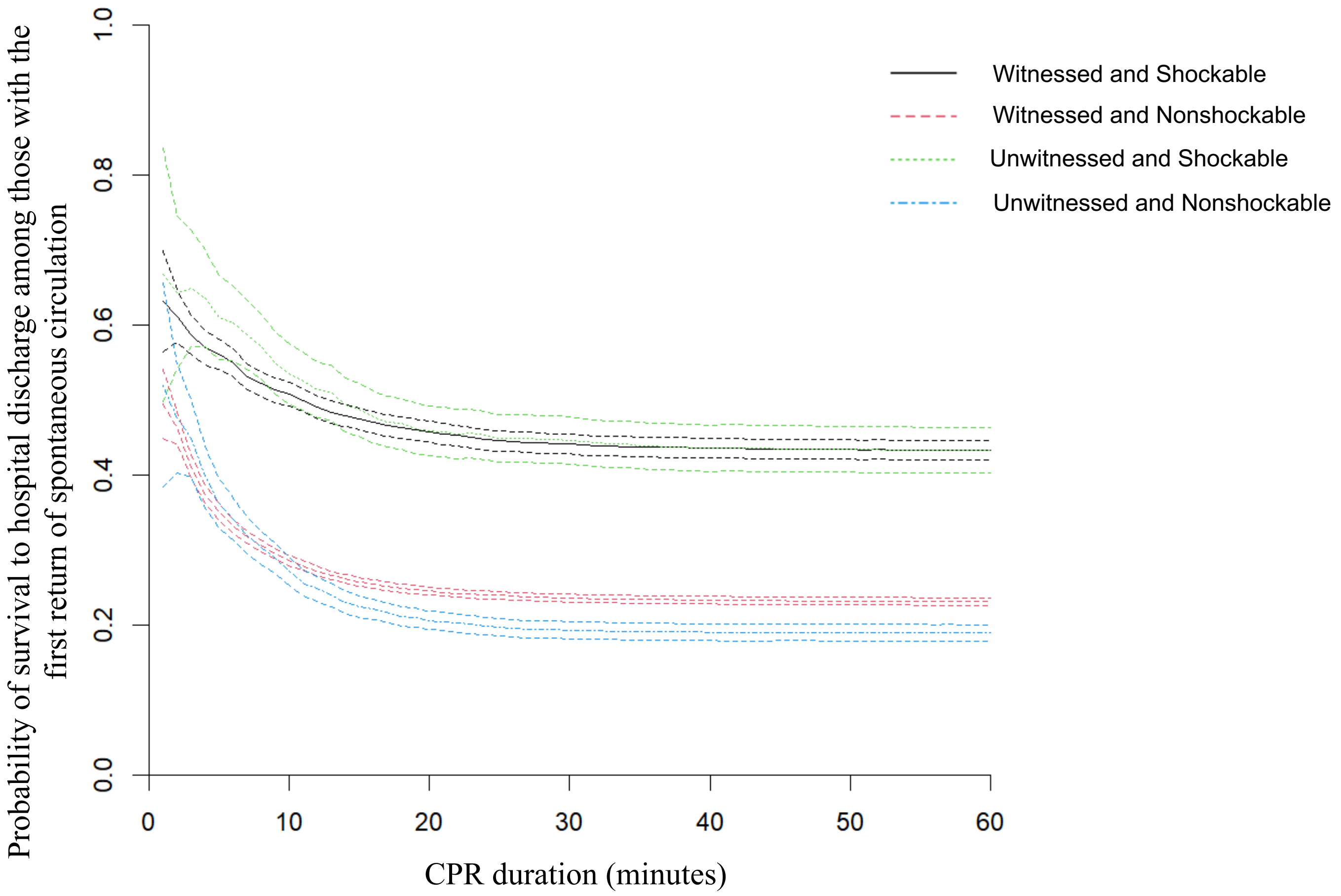

		**CPR duration, minutes**						
		1	10	20	30	40	50	60
Survival to hospital discharge, n / Achieving ROSC, n (%)	Witnessed shockable	125/198 (63.1%)	1941/3826 (50.7%)	2281/4988 (45.7%)	2383/5406 (44.1%)	2416/5547 (43.6%)	2429/5606 (43.3%)	2437/5630 (43.3%)
	Witnessed nonshockaable	222/449 (49.4%)	4766/16680 (28.6%)	5857/23906 (24.5%)	6115/25962 (23.6%)	6189/26597 (23.3%)	6215/26858 (23.1%)	6230/26979 (23.1%)
	Unwitnessed shockable	20/30 (66.7%)	318/595 (53.4%)	398/869 (45.8%)	429/962 (44.6%)	430/988 (43.5%)	434/1000 (43.4%)	434/1004 (43.2%)
	Unwitnessed nonshockable	27/52 (51.9%)	593/2185 (27.1%)	839/4080 (20.6%)	889/4622 (19.2%)	902/4746 (19.0%)	908/4800 (18.9%)	911/4823 (18.9%)

Supplemental Figure E1

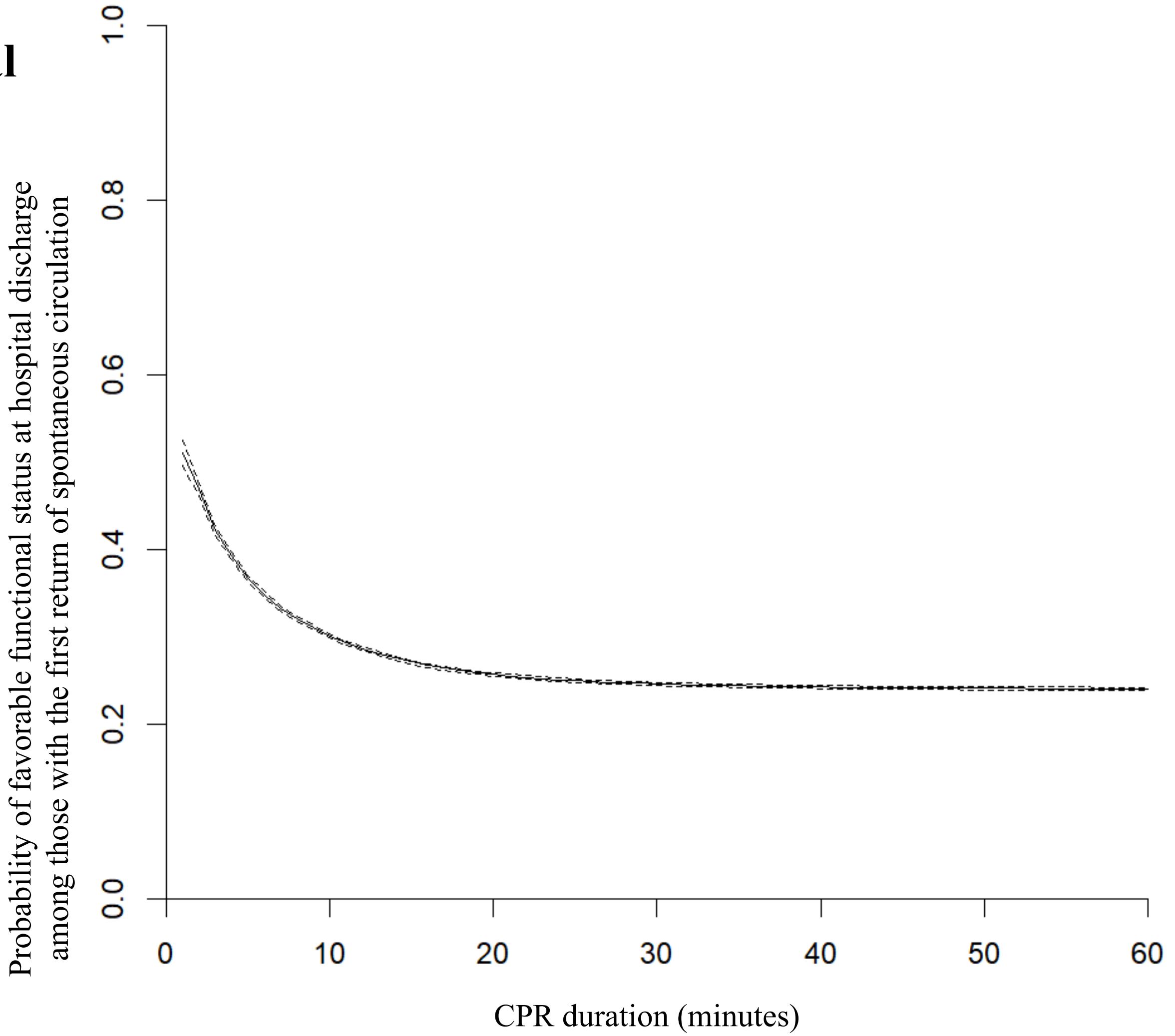

	**CPR duration, minutes**						
	1	10	20	30	40	50	60
Favorable functional status at hospital discharge, n / Achieving ROSC, n (%)	2335/4580 (51.0%)	40528/134710 (30.1%)	48660/189810 (25.6%)	50725/206440 (24.6%)	51458/212553 (24.2%)	51784/215195 (24.1%)	51929/216457 (24.0%)

Supplemental Figure E2

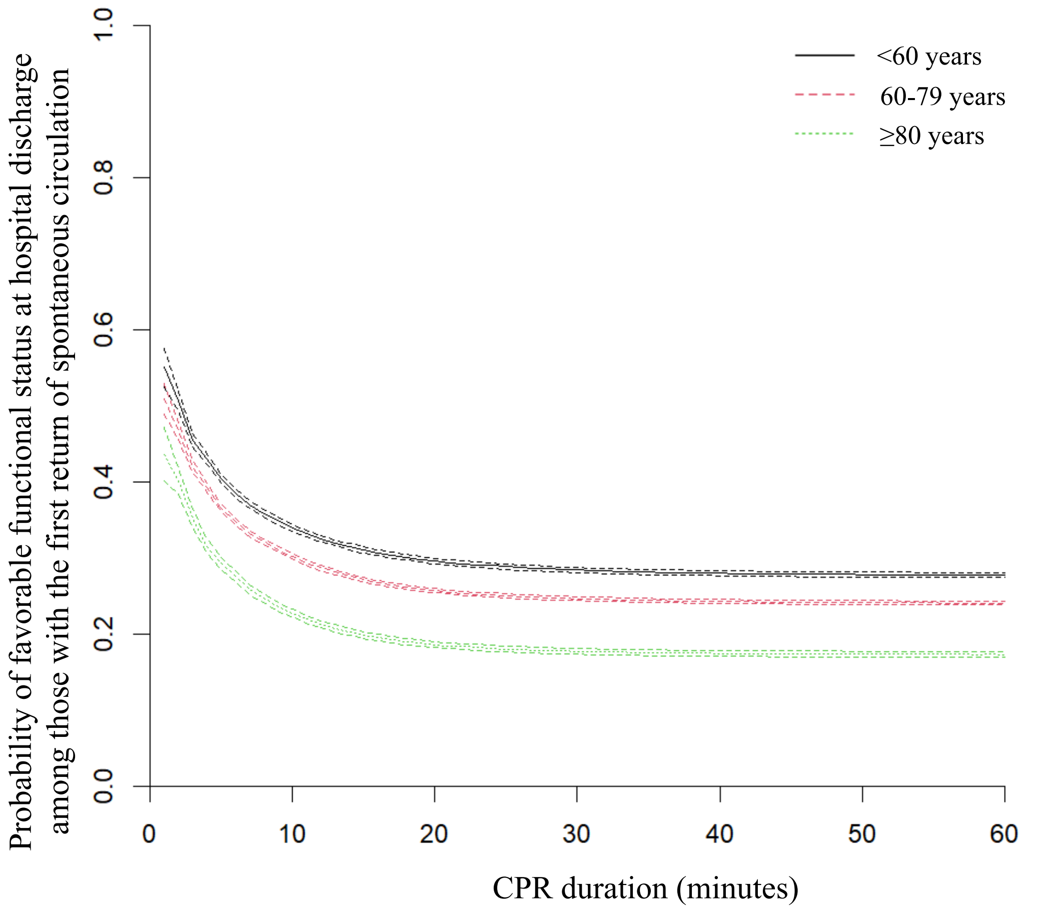

		CPR duration, minutes						
		1	10	20	30	40	50	60
Favorable functional status at hospital discharge, n / Achieving ROSC, n (%)	<60 years	827/1504 (55.0%)	15189/44813 (33.9%)	18373/62149 (29.6%)	19178/67584 (28.4%)	19476/69706 (27.9%)	19611/70613 (27.8%)	19681/71086 (27.7%)
	60-79 years	1172/2305 (50.8%)	19898/65941 (30.2%)	23820/92845 (25.7%)	24819/100837 (24.6%)	25193/103860 (24.3%)	25351/105192 (24.1%)	25411/105798 (24.0%)
	≥80 years	336/771 (43.6%)	5441/23956 (22.7%)	6467/34816 (18.6%)	6728/38019 (17.7%)	6789/38987 (17.4%)	6822/39390 (17.3%)	6837/39573 (17.3%)

Supplemental Figure E3

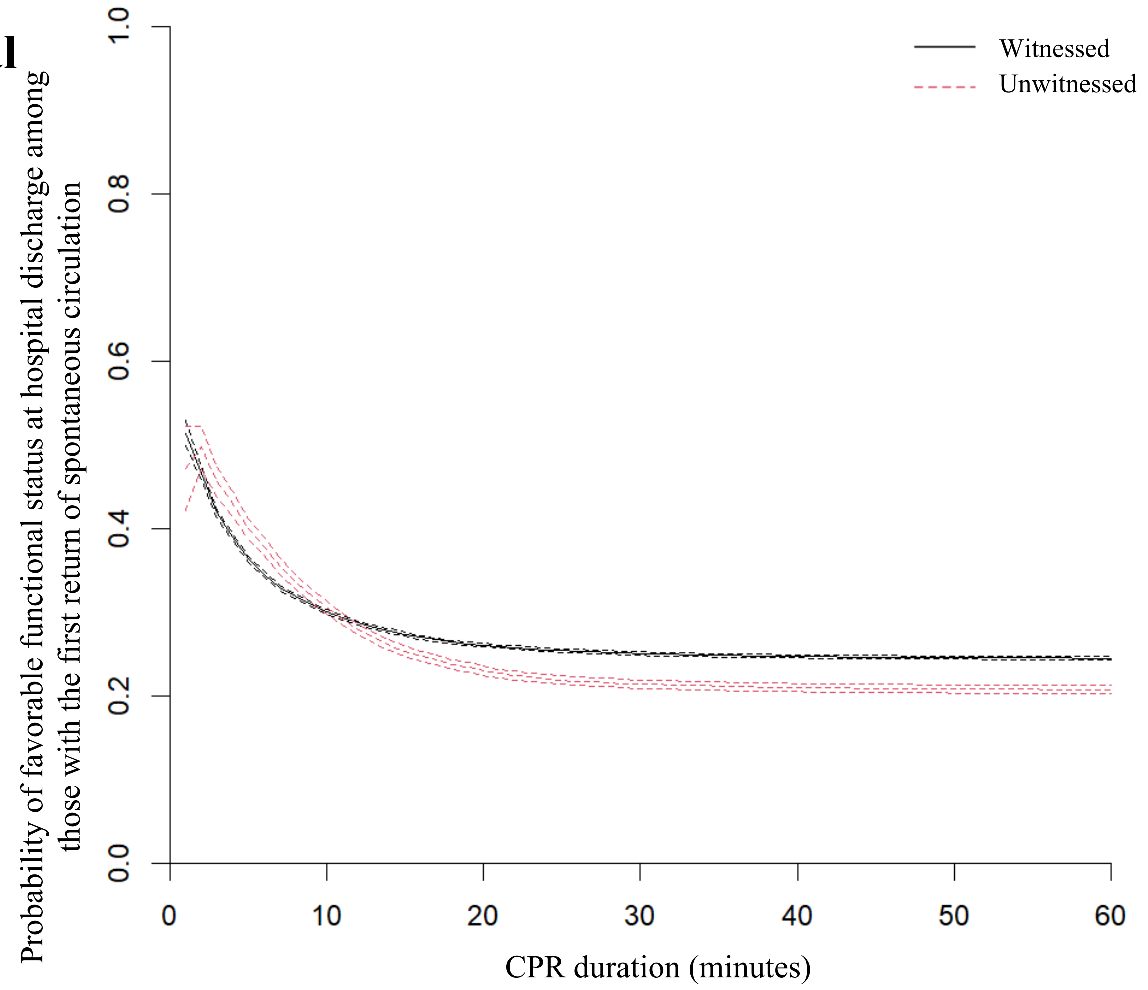

		CPR duration, minutes						
		1	10	20	30	40	50	60
Favorable functional status at hospital discharge, n / Achieving ROSC , n (%)	Witnessed	2155/4198 (51.3%)	36316/120913 (30.0%)	43378/166820 (26.0%)	45192/180556 (25.0%)	45856/185822 (24.7%)	46154/188098 (24.5%)	46286/189202 (24.5%)
	Unwitnessed	180/382 (47.1%)	4212/13797 (30.5%)	5282/22990 (23.0%)	5533/25884 (21.4%)	5602/26731 (21.0%)	5630/27097 (20.8%)	5643/27255 (20.7%)

Supplemental Figure E4

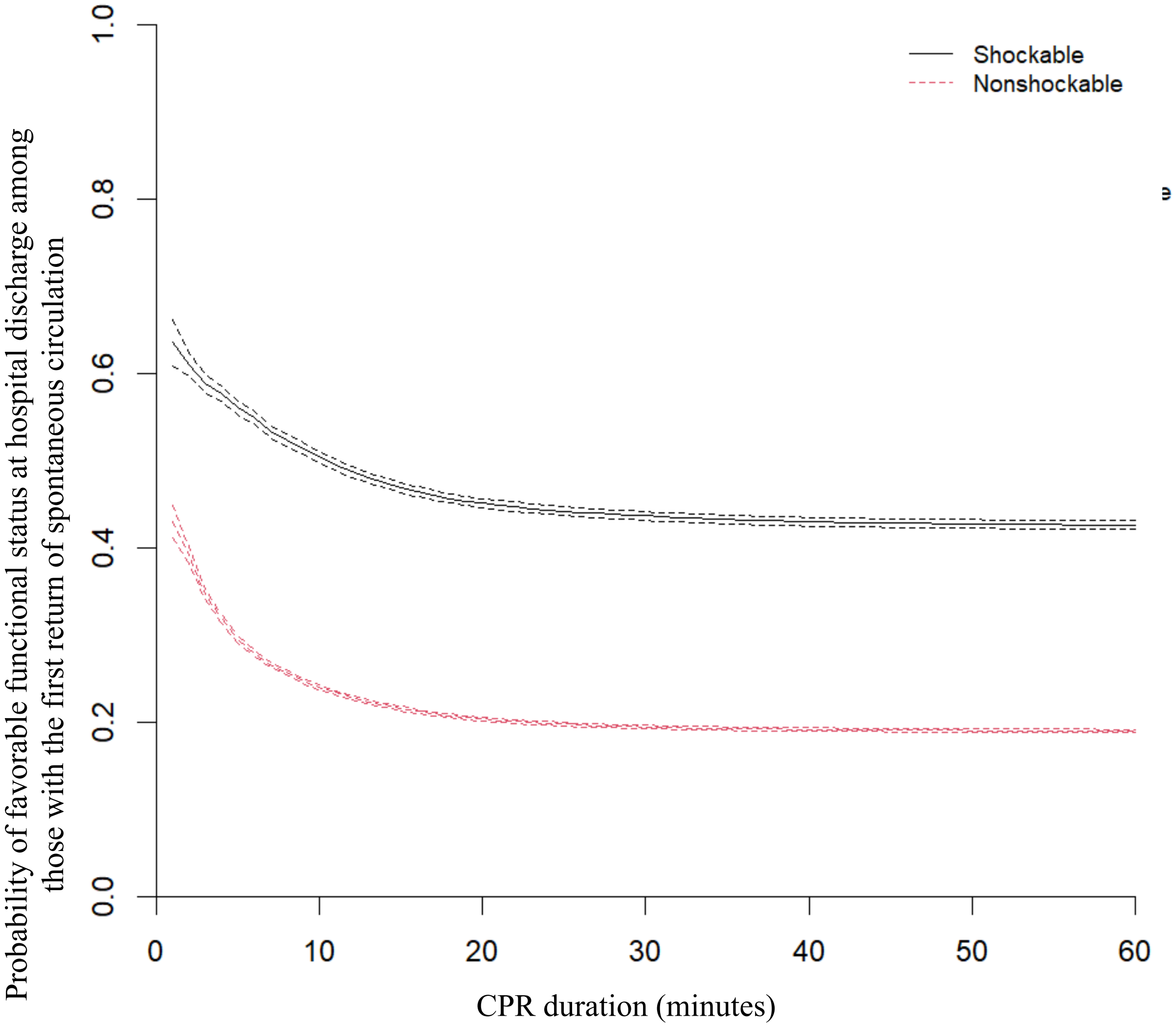

		CPR duration, minutes						
		1	10	20	30	40	50	60
Favorable functional status at hospital discharge, n / Achieving ROSC, n (%)	Shockable	831/1309 (63.5%)	12816/25441 (50.4%)	15011/33313 (45.1%)	15720/36019 (43.6%)	15970/37183 (42.9%)	16087/37683 (42.7%)	16140/37922 (42.6%)
	Nonshockable	1128/2624 (43.0%)	23402/97563 (24.0%)	28754/141476 (20.3%)	29965/154379 (19.4%)	30401/158952 (19.1%)	30584/160918 (19.0%)	30664/161847 (18.9%)

Supplemental Figure E5

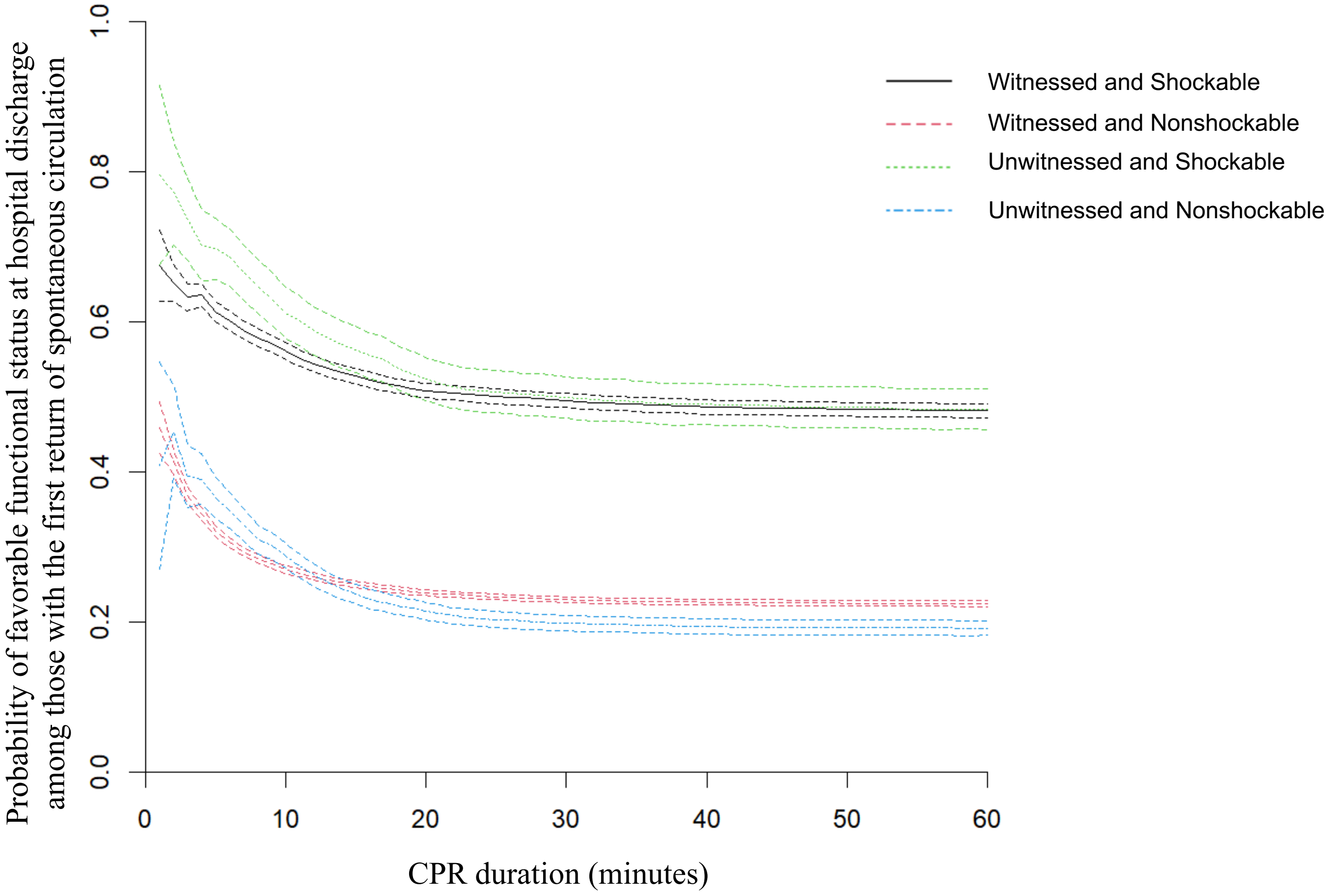

		CPR duration, minutes						
		1	10	20	30	40	50	60
Favorable functional status at hospital discharge, n / Achieving ROSC, n (%)	Witnessed shockable	250/371 (67.4%)	4194/7481 (56.1%)	4943/9730 (50.8%)	5194/10501 (49.5%)	5296/10902 (48.6%)	5341/11061 (48.3%)	5359/11141 (48.1%)
	Witnessed nonshockaable	380/829 (45.8%)	8051/29843 (27.0%)	9912/41498 (23.9%)	10325/45017 (22.9%)	10479/46374 (22.6%)	10547/46963 (22.5%)	10585/47272 (22.4%)
	Unwitnessed shockable	35/44 (79.5%)	485/793 (61.1%)	588/1124 (52.3%)	615/1233 (49.9%)	627/1281 (48.9%)	631/1299 (48.6%)	633/1311 (48.3%)
	Unwitnessed nonshockable	20/49 (40.8%)	808/2811 (28.7%)	1047/4897 (21.4%)	1112/5612 (19.8%)	1123/5798 (19.4%)	1131/5885 (19.2%)	1134/5922 (19.1%)

Supplemental Figure E6

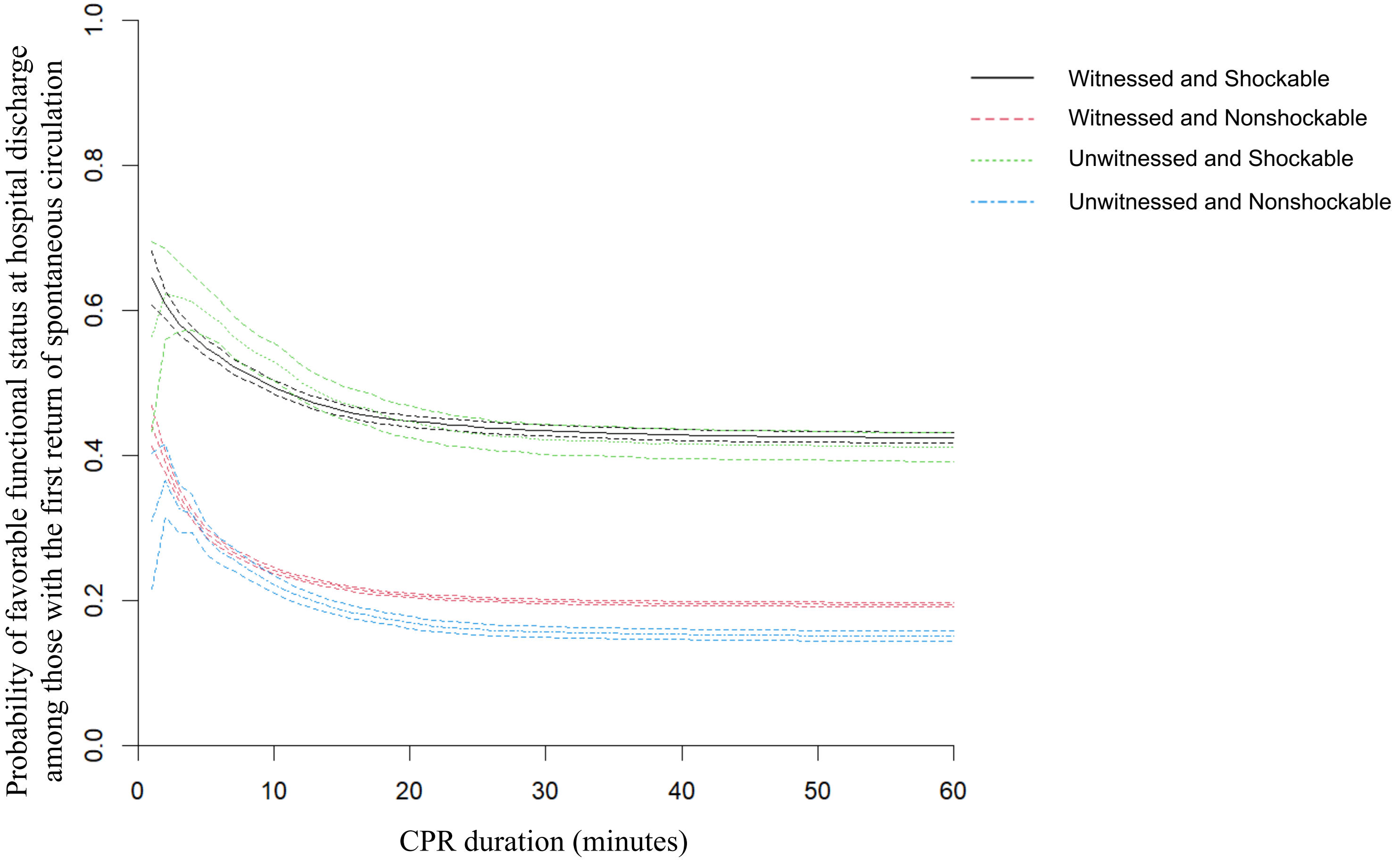

		CPR duration, minutes						
		1	10	20	30	40	50	60
Favorable functional status at hospital discharge, n / Achieving ROSC, n (%)	Witnessed shockable	405/629 (64.4%)	5769/11686 (49.4%)	6728/15071 (44.6%)	7035/16205 (43.4%)	7143/16701 (42.8%)	7195/16929 (42.5%)	7221/17033 (42.4%)
	Witnessed nonshockaable	531/1204 (44.1%)	10283/42565 (24.2%)	12474/60427 (20.6%)	12993/65539 (19.8%)	13203/67499 (19.6%)	13288/68347 (19.4%)	13314/68738 (19.4%)
	Unwitnessed shockable	31/55 (56.4%)	768/1452 (52.9%)	904/2026 (44.6%)	940/2229 (42.2%)	952/2292 (41.5%)	957/2317 (41.3%)	959/2333 (41.1%)
	Unwitnessed nonshockable	29/94 (30.9%)	1011/4567 (22.1%)	1362/8049 (16.9%)	1419/9086 (15.6%)	1440/9420 (15.3%)	1443/9555 (15.1%)	1447/9607 (15.1%)

Supplemental Figure E7

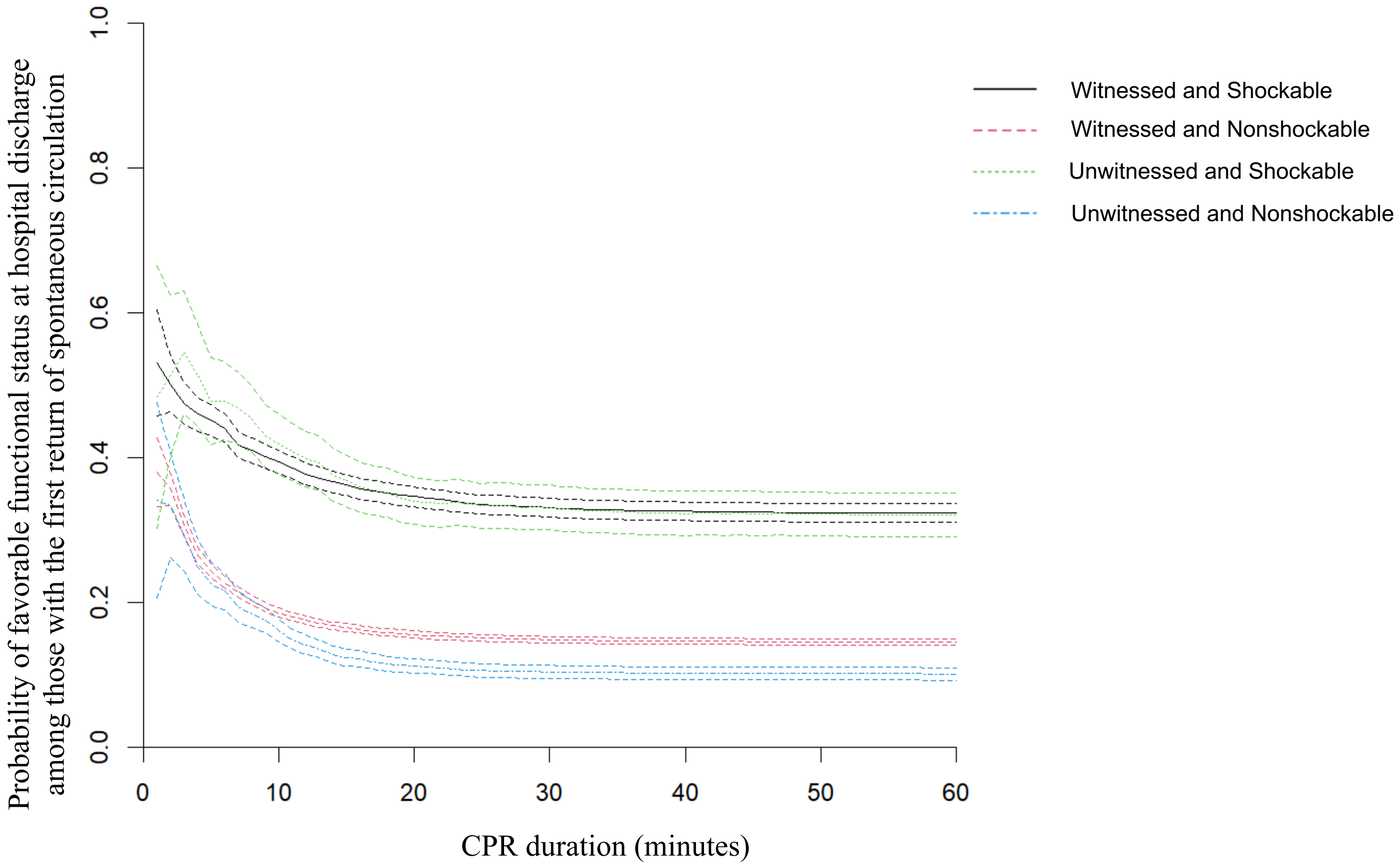

		CPR duration, minutes						
		1	10	20	30	40	50	60
Favorable functional status at hospital discharge, n / Achieving ROSC, n (%)	Witnessed shockable	96/181 (53.0%)	1373/3486 (39.4%)	1577/4564 (34.6%)	1643/4965 (33.1%)	1658/5095 (32.5%)	1666/5153 (32.3%)	1671/5176 (32.3%)
	Witnessed nonshockaable	152/401 (37.9%)	2916/15706 (18.6%)	3524/22697 (15.5%)	3656/24687 (14.8%)	3693/25304 (14.6%)	3709/25558 (14.5%)	3717/25675 (14.5%)
	Unwitnessed shockable	14/29 (48.3%)	227/543 (41.8%)	271/798 (34.0%)	293/886 (33.1%)	294/912 (32.2%)	297/924 (32.1%)	297/928 (32.0%)
	Unwitnessed nonshockable	16/47 (34.0%)	333/2071 (16.1%)	435/3908 (11.1%)	460/4438 (10.4%)	463/4557 (10.2%)	466/4610 (10.1%)	467/4633 (10.1%)

Supplemental Figure F1

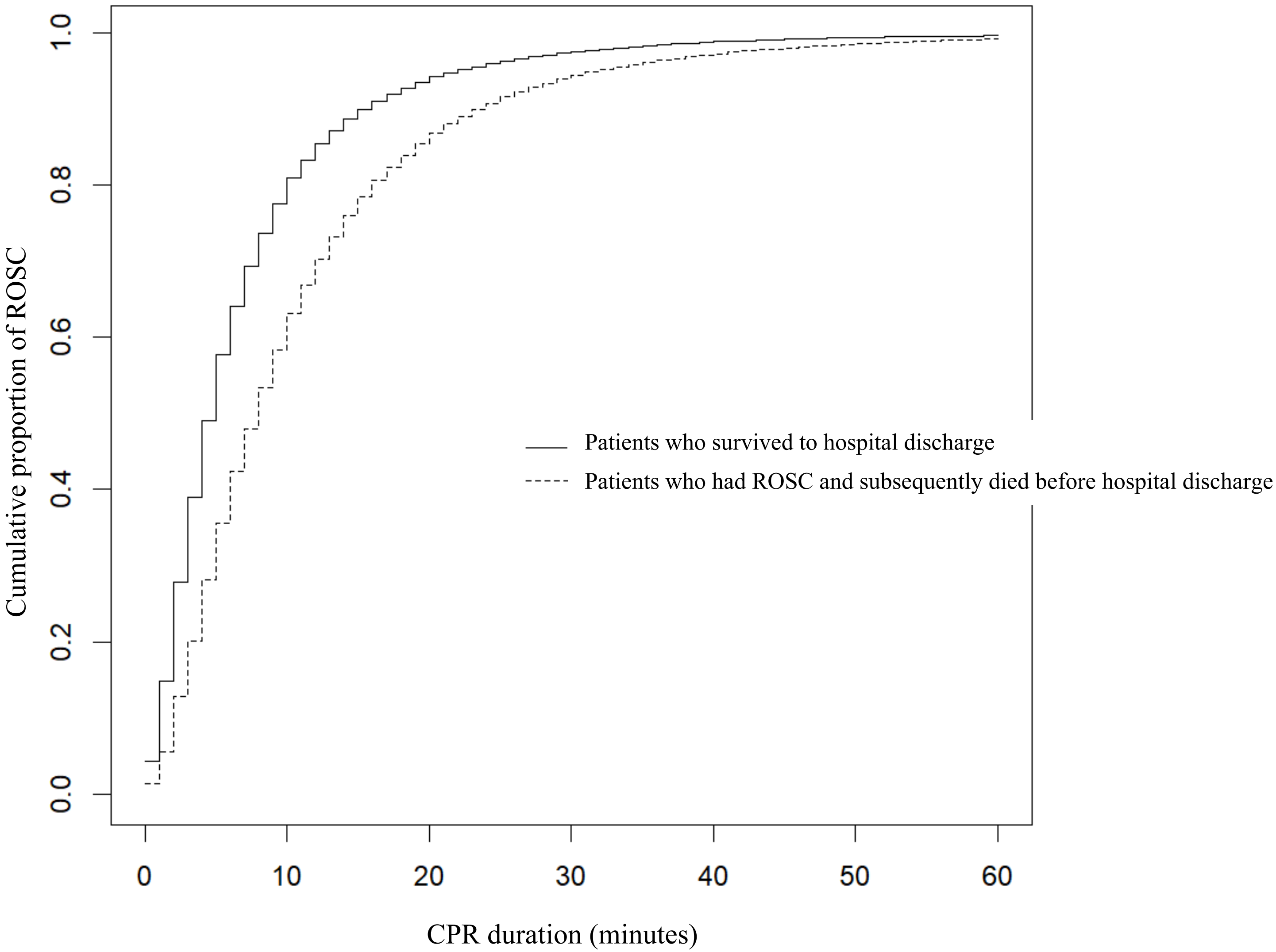

	Percentile CPR duration, minutes (95% CI)	
	95th percentile	99th percentile
Patients who survived to hospital discharge	22 (22-23)	43 (42-44)
Patients who had ROSC and subsequently died before hospital discharge	32 (32-32)	57 (56-58)

Supplemental Figure F2

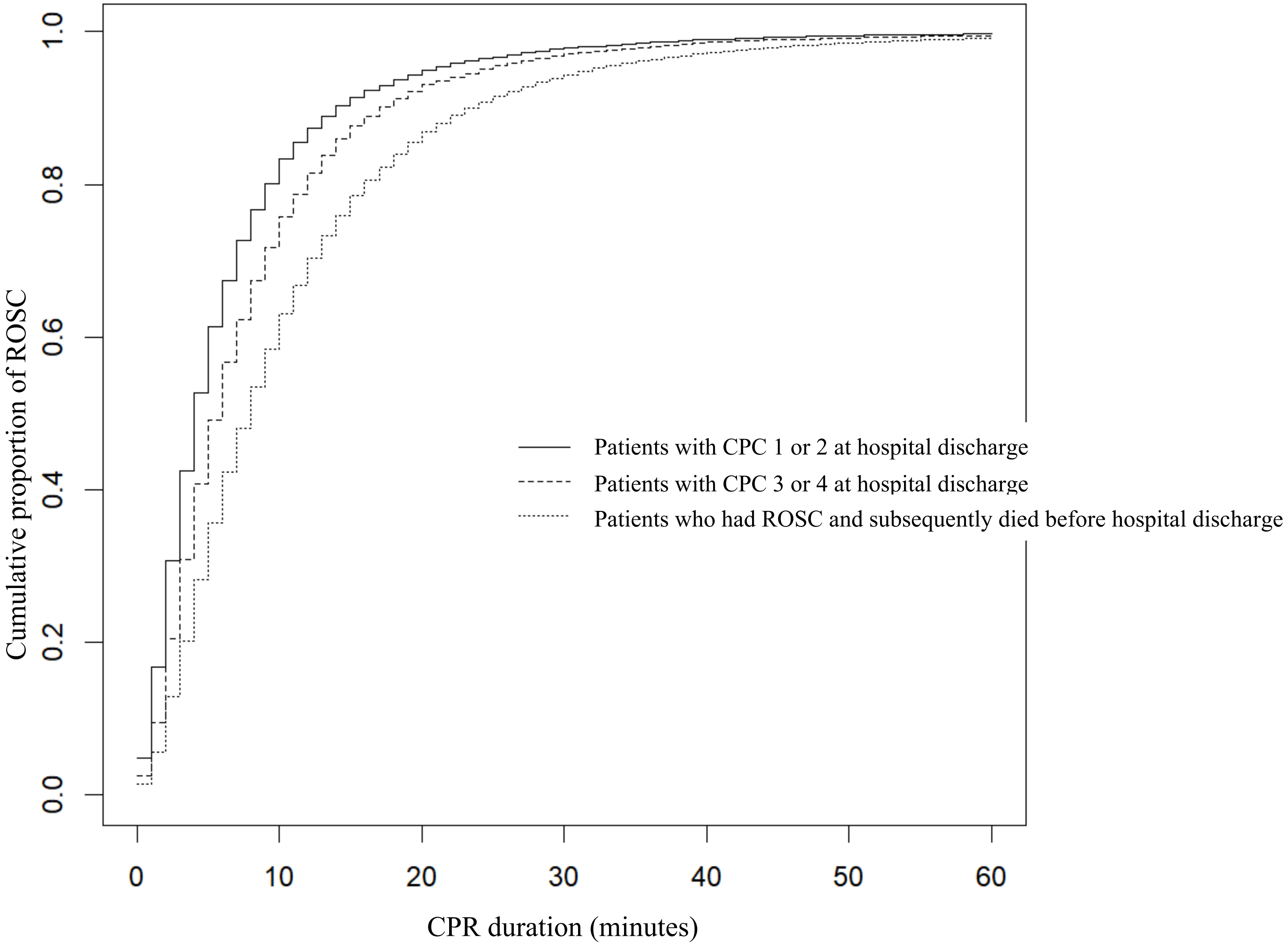

	Percentile CPR duration, minutes (95% CI)	
	95th percentile	99th percentile
Patients with CPC 1 or 2 at hospital discharge	21 (20-21)	41 (39-43)
Patients with CPC 3 or 4 at hospital discharge	24 (24-25)	47 (43-51)
Patients who had ROSC and subsequently died before hospital discharge	32 (32-32)	57 (56-58)

Supplemental Figure G

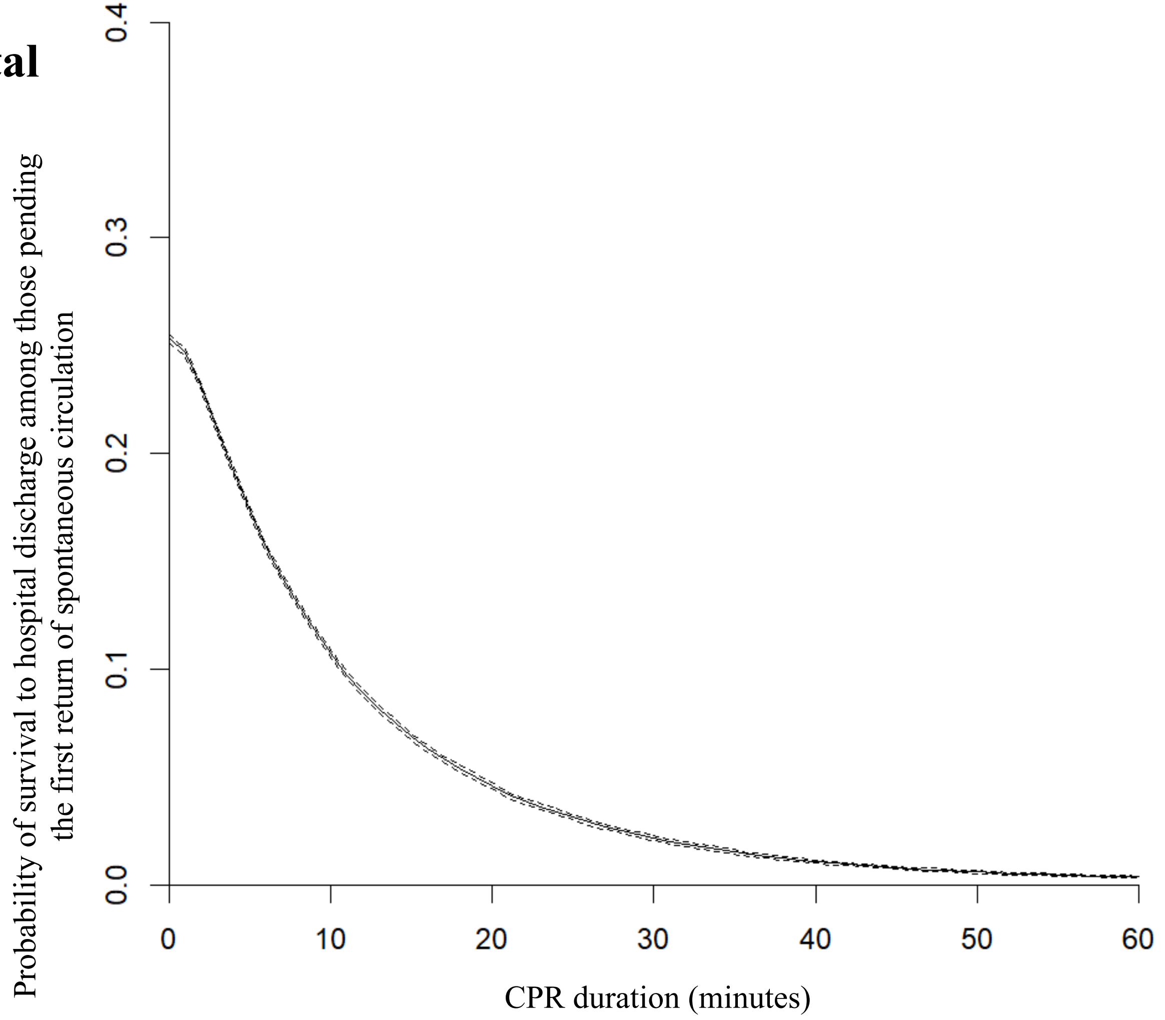

	CPR duration, minutes						
	1	10	20	30	40	50	60
Survival to hospital discharge, n / Receiving CPR, n (%)	50,900/206,240 (24.7%)	11,961/111,219 (10.8%)	3,494/76,067 (4.6%)	1,432/65,701 (2.2%)	663/61,802 (1.1%)	352/60,113 (0.6%)	210/59,293 (0.4%)

Supplemental Figure H

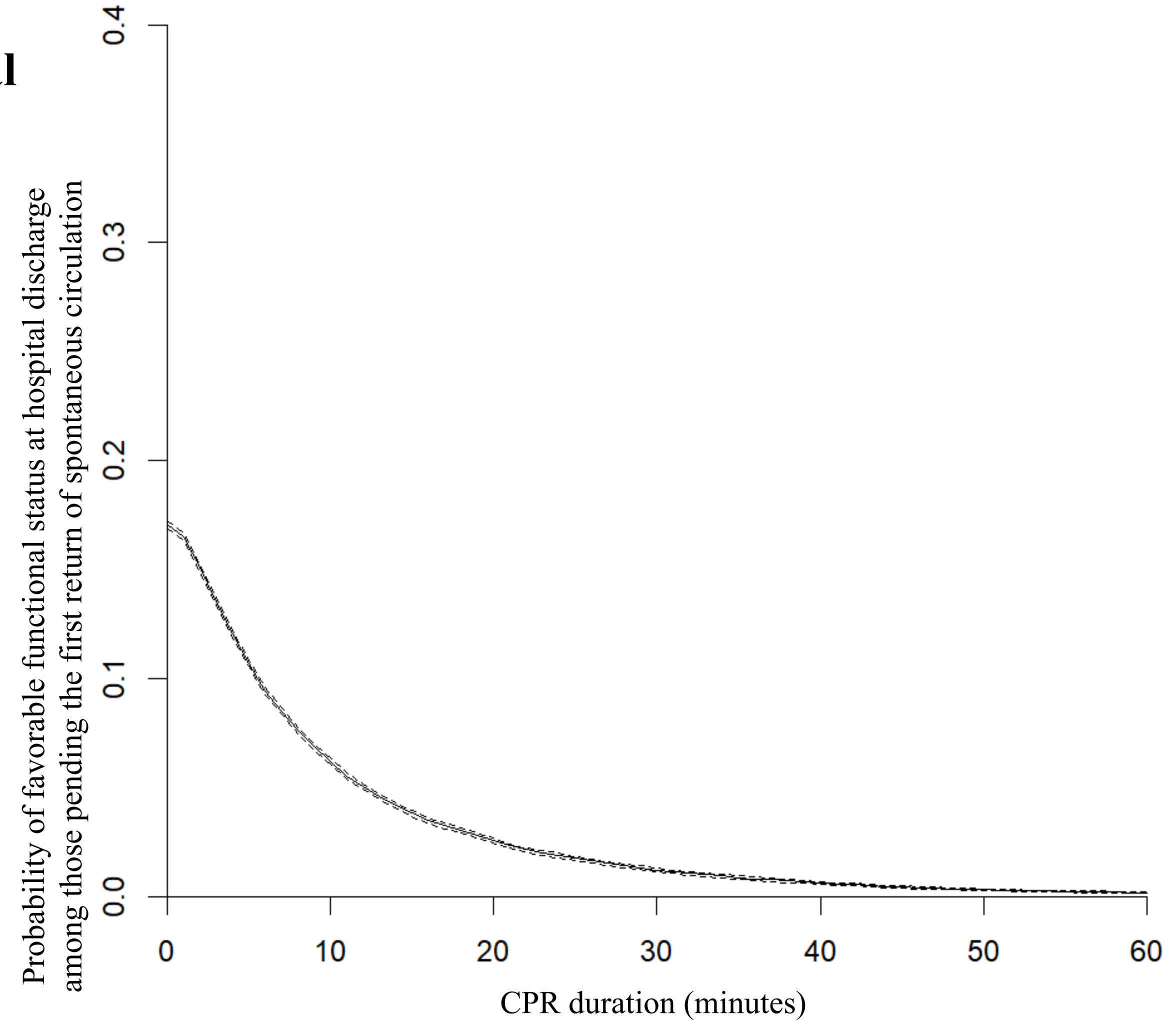

	CPR duration, minutes						
	1	10	20	30	40	50	60
Favorable functional status at hospital discharge, n / Receiving CPR, n (%)	32,078/194,267 (16.5%)	6,689/107,944 (6.2%)	1,916/75,049 (2.6%)	788/65,288 (1.2%)	368/61,614 (0.6%)	182/60,005 (0.3%)	103/59,224 (0.2%)

Supplemental Figure I

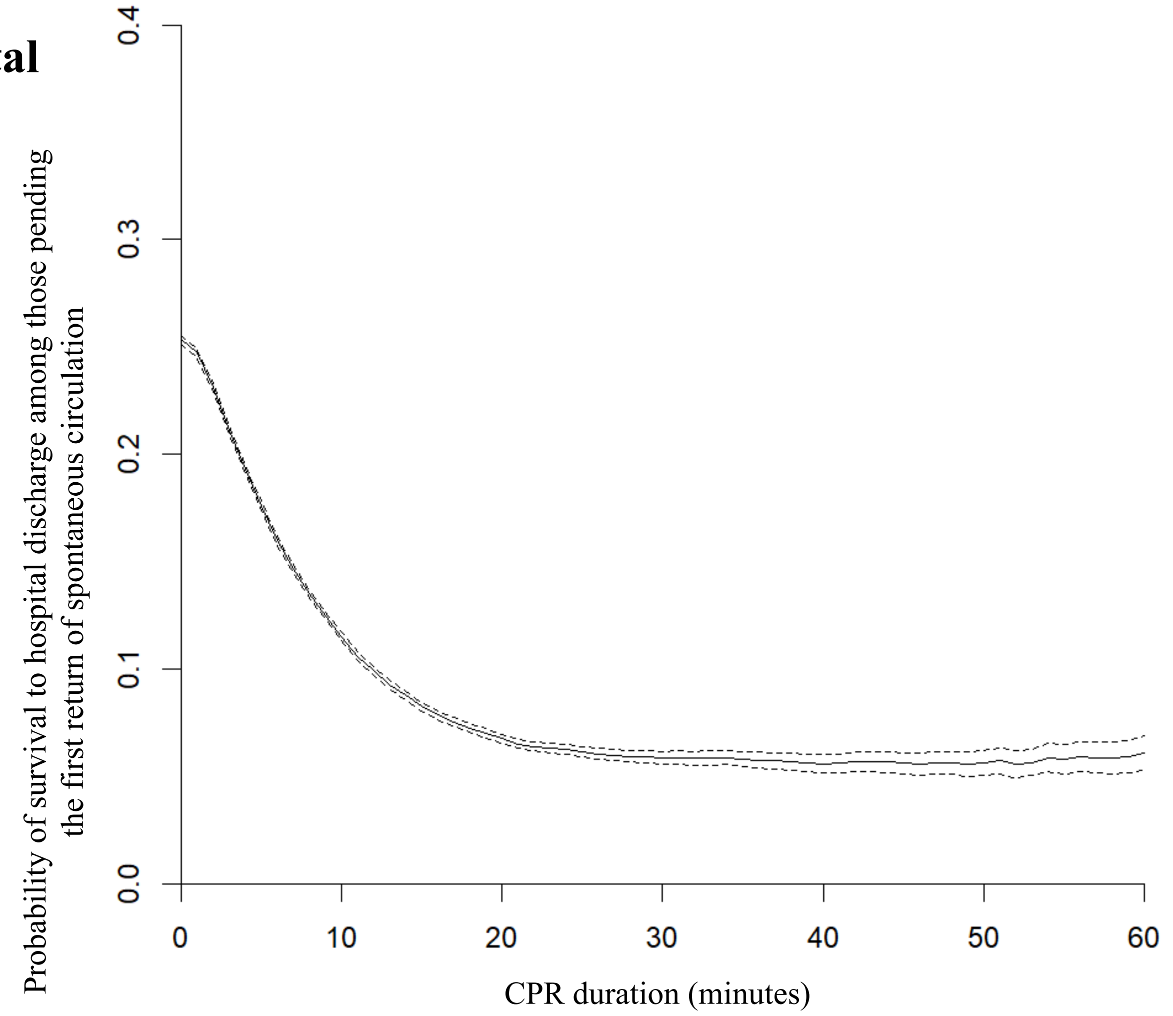

	CPR duration, minutes						
	1	10	20	30	40	50	60
Survival to hospital discharge, n / Receiving CPR, n (%)	50900/206089 (24.7%)	11961/104013 (11.5%)	3494/51758 (6.8%)	1432/24434 (5.9%)	663/11871 (5.6%)	352/6242 (5.6%)	210/3451 (6.1%)

Supplemental Figure J

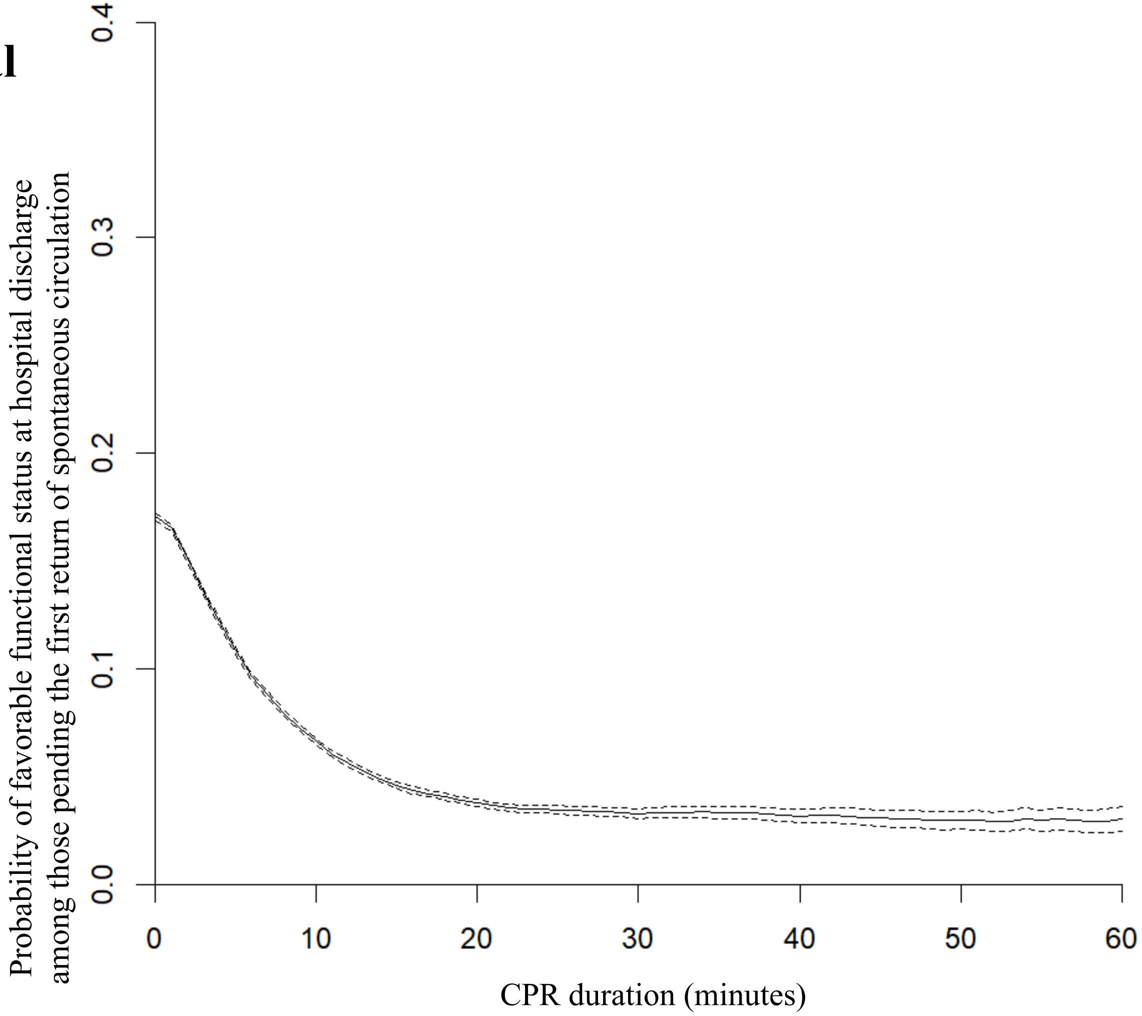

	CPR duration, minutes						
	1	10	20	30	40	50	60
Favorable functional status at hospital discharge, n / Receiving CPR, n (%)	32078/194116 (16.5%)	6689/100738 (6.6%)	1916/50740 (3.8%)	788/24021 (3.3%)	368/11683 (3.1%)	182/6134 (3.0%)	103/3382 (3.0%)

Supplemental Figure K

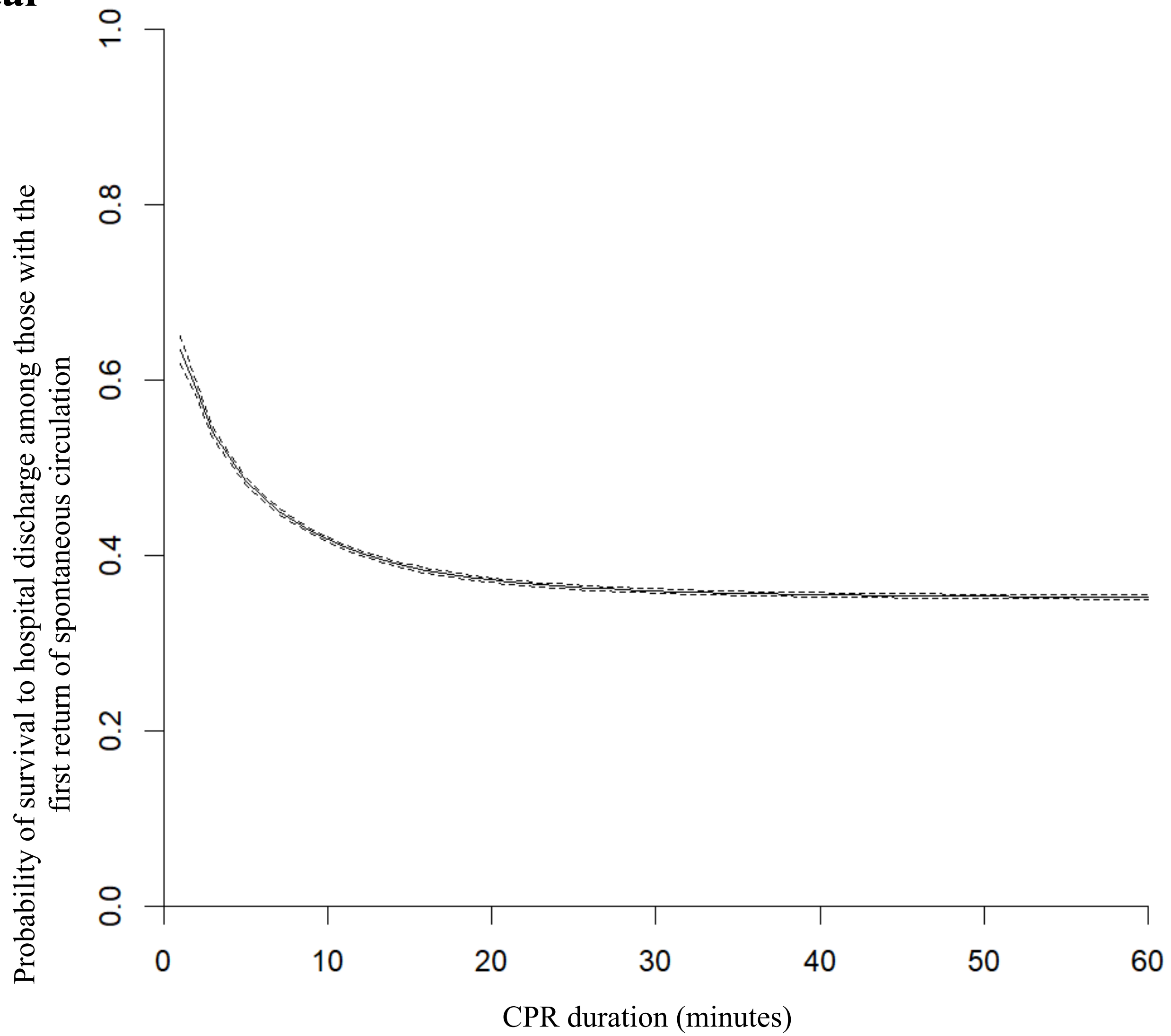

	CPR duration, minutes						
	1	10	20	30	40	50	60
Survival to hospital discharge, n / Achieving ROSC, n (%)	2272/3587 (63.3%)	41211/98608 (41.8%)	49678/133760 (37.1%)	51740/144126 (35.9%)	52509/148025 (35.5%)	52820/149714 (35.3%)	52962/150534 (35.2%)

Supplemental Figure L

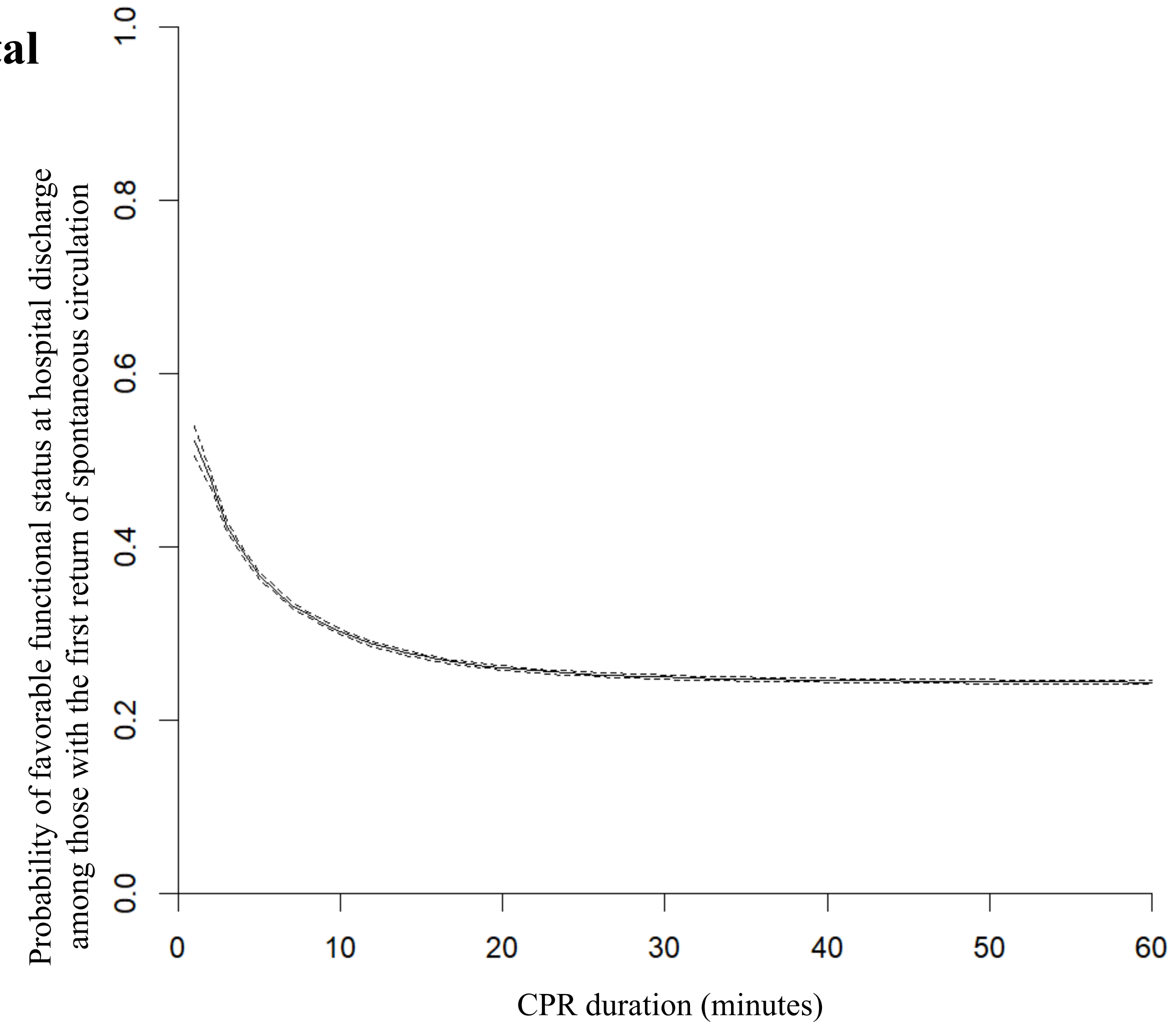

	CPR duration, minutes						
	1	10	20	30	40	50	60
Favorable functional status at hospital discharge, n / Achieving ROSC, n (%)	1627/3118 (52.2%)	27016/89441 (30.2%)	31789/122336 (26.0%)	32917/132097 (24.9%)	33337/135771 (24.6%)	33523/137380 (24.4%)	33602/138161 (24.3%)
